# A revised radiocarbon calibration curve 350–250 BCE impacts high-precision dating of the Kyrenia Ship

**DOI:** 10.1371/journal.pone.0302645

**Published:** 2024-06-26

**Authors:** Sturt W. Manning, Brita Lorentzen, Martin Bridge, Michael W. Dee, John Southon, Madeleine Wenger

**Affiliations:** 1 Cornell Tree-Ring Laboratory, Department of Classics, and Cornell Institute of Archaeology and Material Studies, Cornell University, Ithaca, NY, United States of America; 2 The Cyprus Institute, Nicosia, Cyprus; 3 Department of Anthropology, University of Georgia, Athens, GA, United States of America; 4 Oxford Dendrochronology Laboratory, Mapledurham, United Kingdom; 5 Centre for Isotope Research, Faculty of Science and Engineering, University of Groningen, Groningen, The Netherlands; 6 Department of Earth System Science, University of California, Irvine, CA, United States of America; Israel Antiquities Authority, ISRAEL

## Abstract

The Kyrenia Ship, found off the north coast of Cyprus, is a key vessel in the history of scientific underwater excavations and in the history of Greek shipbuilding. The first volume of the site’s final publication appeared in 2023 and provides detailed archaeological information tightly constraining the dating of the ship. A very specific date range is proposed: ca. 294–290 BCE, but is based on a less than certain reading of one coin recovered from the ship. While there is clear benefit to finding high-precision dates for the Kyrenia Ship and its rich assemblage using independent scientific dating (combined with Bayesian chronological modeling), efforts to do so proved more challenging and complex than initially anticipated. Strikingly, extensive radiocarbon dating on both wooden materials from the ship and on short-lived contents from the final use of the ship fail to offer dates using the IntCal20 calibration curve—the current Northern Hemisphere radiocarbon calibration curve at the time of writing—that correspond with the archaeological constraints. The issue rests with a segment of IntCal20 ca. 350–250 BCE reliant on legacy pre-AMS radiocarbon data. We therefore measured new known-age tree-ring samples 350–250 BCE, and, integrating another series of new known-age tree-ring data, we obtained a redefined and more accurate calibration record for the period 433–250 BCE. These new data permit a satisfactory dating solution for the ship and may even indicate a date that is a (very) few years more recent than current estimations. These new data in addition confirm and only very slightly modify the dating recently published for the Mazotos ship, another Greek merchant ship from the southern coast of Cyprus. Our work further investigated whether ship wood samples impregnated with a common preservative, polyethylene glycol (PEG), can be cleaned successfully, including a known-age test.

## Introduction

The Hellenistic period Kyrenia Ship, which was found, and then excavated off the northern coast of Cyprus in 1967–1969 [1–3], provides key evidence in studying the evolution of Greek shipbuilding traditions and Mediterranean maritime commercial exchange [4, 5]. Originally dated from a single radiocarbon (^14^C) measurement rather approximately 288±62 BCE [2, 3], Katzev and Swiny’s recent publication [1] reports on the ship’s dating as of 2023.

Research on 7 recovered coins and plentiful ceramics, in particular amphora stamps, from the shipwreck led to the assessment of a date for the last voyage and ultimate wrecking around 294–290 BCE. The specificity of this date is conspicuous. A single coin (C7) is key [6]; Keen argues for a *terminus post quem* (TPQ) for this coin of 294 BCE ([6] at p.390). Otherwise, the ceramics and other coins (and/or choosing a potential earlier minimum TPQ dating for C7 at 310–306 BCE) collectively only define a likely possible date range for the final voyage from the late 4^th^ century (e.g. after ca. 325/320 BCE) to the early 3^rd^ century BC (e.g. before 280/270 BCE), quantified as likely about 310–290 BCE ([6, 7] at pp.142-145, [8] at pp.272-274).

The coins are all “in a state of severe deterioration, making precise attributions impossible” ([6] at p.389). The only ‘hard’ evidence, versus sets of assumptions, revolves around the general types that can be “cautiously ascertained for all but a single coin” ([6] at p.389). Five of the coins belong to two types associated with Alexander, indicating a TPQ of 336 BCE for one group and a TPQ of 334 BCE for the other group ([6] at pp.389-390). Coin C7 is a Ptolemaic type and therefore should set a minimum TPQ of 310–306 BCE (as may a round sterling silver object, Pb11/C8: [6] at p.389). However, in this case there are additional observations that likely indicate a later date from/after 294 BCE with a suggested range of 294–290 BCE ([6] at pp.390-391). The independent information from the study of the Rhodian amphora stamps might also be argued to indicate a minimum TPQ a few years after 305 BCE and a date likely in the mid to late 290s BCE, but there are also a number of uncertainties involved (see [7] at pp.142-145).

Coin C7 is thus the critical evidence. However, it is stated of the key C7 coin that “unfortunately, reading this coin is extremely difficult given the state of deterioration and this attribution must be regarded with caution” ([6] at p.290, Fig 6.1.7). Keen ([6] at p.391) even ventures that “if the coin [C7] is disregarded as a data-point due to the difficulties of attribution, a date of deposition around 300 BCE could be suggested…”. Thus a considerable chronological weight rests upon this one less-than-certain coin (C7), and the high-precision dating of the well-known Kyrenia Ship and its rich assemblage could therefore benefit from additional independent dating. An independent date would in turn usefully inform discussions on ceramic types/occurrences and especially attempts to link attested epigraphic information (names of Rhodian priests).

We therefore sought to offer an independent assessment of the likely dating of the Kyrenia Ship and its last voyage using available tree-ring and ^14^C evidence as a high-resolution dating test-case and to test, clarify, and compare with the archaeological dating of the ship and its contents. Based on the extensive archaeological material and its assessment, the total possible dating window for the final voyage and wrecking of the ship may be very conservatively limited. A (minimum) TPQ of 334 BCE may be applied with reasonable confidence from the coin evidence (see above). It is further likely that a TPQ of ca. 310 BCE is plausible (see above) and perhaps 294 BCE, but this is less certain.

In reverse, it is clear the ship’s contents, coins and ceramics, date no later than the earlier 3^rd^ century BCE. The focus of work in [1] was to demonstrate that the Kyrenia Ship contents indicated that the shipwreck was not late 4^th^ century BCE (as often suggested in the past) but instead early 3^rd^ century BCE; hence there was little discussion of possible dates beyond about 290 BCE. Addressing the latest plausible date, we may observe two factors: (i) the extensive Kyrenia Ship cargo shows no signs of the later, shorter, and wider-bodied Rhodian amphora forms with a shorter neck and steeply sloping rim that became prevalent in the 280s to 260s BCE (RhI.2 and RhI.3); and (ii) stamps that appear associated only with these new amphora forms and which are not attested on the taller ‘Kyrenia’ form (RhI.1) are absent in the wreck’s cargo ([9] at p.140 and Fig 2; we thank a reviewer for sharing their expertise on this topic). Hence we might also reasonably impose a very conservative *terminus ante quem* (TAQ) of 270 BCE (from the ceramics, see [8] at p.272).

The research question, therefore, is to check, investigate and clarify the specific suggested dating of 294–290 BCE [1] using tree-ring and ^14^C dating approaches. However, this task revealed that there are important complications here also, which we seek to address in this paper.

First, what might have been straightforward key evidence—a reasonable tree-ring sequence and in turn a tree-ring sequenced ^14^C ‘wiggle-match’ date—is complicated because the relevant wood sample from bow sheathing (KYR-8) (like most of the ship’s preserved timbers) was treated with Polyethylene glycol (PEG), rendering its ^14^C dating a challenge, since PEG introduces much older contaminating petroleum-derived carbon which has long proved difficult to remove—as demonstrated by an initial set of ^14^C dates on this sample. In this paper, we combine into a Bayesian chronological model dates on (i) wood samples which were not treated with PEG (or any other contaminant) (ii) newly cleaned (2022) tree-ring samples of KYR-8 (contrasted with a previous sample set from 2014) which have likely removed nearly all PEG contamination (with this removal strategy tested using known-age archaeological wood samples treated with PEG more than a decade ago), and (iii) short-lived sample material from the shipwreck (no PEG), in order to define the date of the ship’s timbers and the date of the subsequent last voyage.

Successful implementation of our chronological model requires an accurate underlying chronological reference: the Northern Hemisphere ^14^C calibration curve. Our work finds that use of the current Northern Hemisphere IntCal20 ^14^C calibration curve [10] suggests calendar ages for the Kyrenia Ship and wrecking incompatible with the archaeological assessment. However, on examination, we find that IntCal20 is relatively poorly defined for the period from ca. 350–250 BCE with, in particular, a lack of modern AMS ^14^C data informing the dataset. Studies in recent years have illustrated the need to revise the ^14^C calibration curve in several such cases of legacy-only data (e.g. [11–13]). Since the ability to determine calendar age estimates accurately from ^14^C measurements depends on the accuracy and precision of the calibration curve, we therefore measured and report a number of known-age single (annual) tree-ring samples across the interval 431–250 BCE and incorporated also another recently measured set of annual measurements [14] to give altogether a better-defined ^14^C calibration record 433–250 BCE.

Testing the legacy IntCal20 data in this time window turns out to be important. Our new calibration data substantially adjust what would have been the calendar age placement of the Kyrenia Ship ^14^C evidence versus IntCal20. They point to a likely date range that is much nearer to, but not entirely compatible with, the above coin-ceramic-based estimate—indicating the likely chronological range for further detailed investigation and critique. In particular, the new ^14^C evidence might suggest a wrecking date sometime in the 280s BCE. Additionally, the new ^14^C calibration data fine tune, but overall confirm, the earlier 4^th^ century BCE date proposed for another Greek commercial ship, the Mazotos ship found off southern Cyprus [15].

## Materials and methods

Samples employed and first reported in this project were either provided by the directors of the Kyrenia excavation and study project, Susan Womer Katzev and Laina Wylde Swiny (see [1]), or come from materials in the archives of the Cornell Tree Ring Laboratory, or were supplied from materials held by MB from dendrochronological work in the UK.

### Wood and long-lived samples

**Fig 1:**
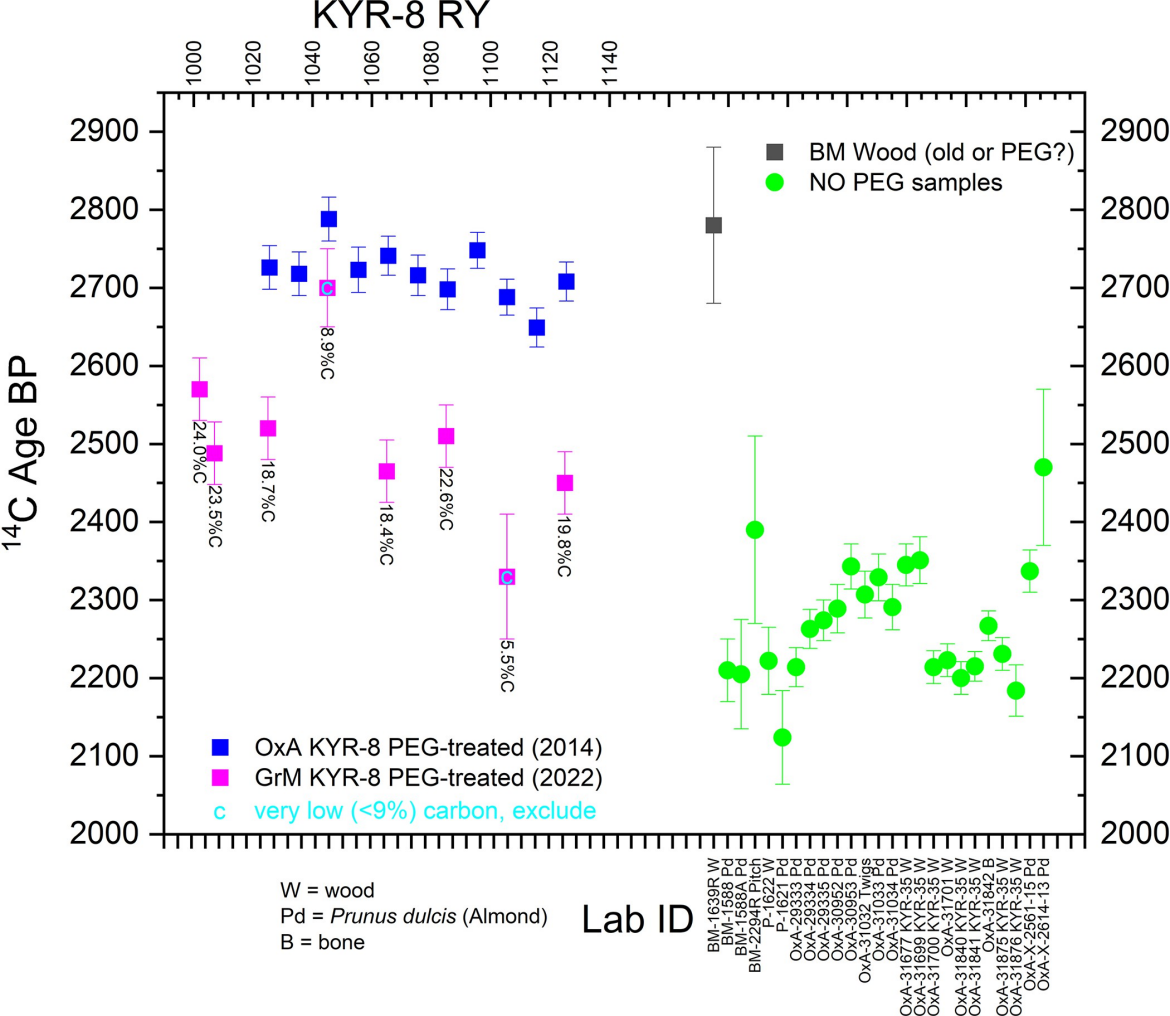
The set of ^14^C dates in Table 1 compared to each other in terms of ^14^C dates expressed in ^14^C years BP. The cleaned GrM versus not entirely cleaned OxA dates on the KYR-8 samples are compared on the left (the two GrM dates with very low percent carbon, less than half of the other samples, are not used in the analysis). The other dates are shown on the right. They fall within a similar age range. The OxA-X dates and the previously run non-AMS ^14^C dates (P-, BM-) are not used in our analysis—see the main text and Table 1. 1σ error bars are shown.

A number of wood samples from the Kyrenia Ship were taken and previously studied by Peter Ian Kuniholm and his team [16]. Despite suggestions to the contrary (e.g. [17] at Fig 1), the tree-ring sequences from these samples do not at present plausibly match (that is crossdate) with any available reliable and dated tree-ring sequences we know of following robust dendrochronological methods. Further, since the relevant datasets employed or mentioned in [17] are neither properly reported, nor is appropriate information provided about the supposed crossdating, it is difficult to accept these claims as substantiated. Basic statistics in particular, and information about the visual similarities supporting strong correlation among the tree-ring series from samples of the same or very similar species from similar growth environments, are missing, leaving an absence of the information expected following standard dendrochronological methods and norms for a plausible and rigorous crossdate (e.g. [18–21]). Hence the present project sought to consider the dating of selected Kyrenia Ship wood elements by ^14^C—including the integration of dendrochronology and ^14^C through so-called ^14^C-wiggle-matching (e.g. [22–24]) and as applied to the case of a shipwreck (e.g. [15]). The aim of analysis of the wood and other long-lived sample material is to estimate a TPQ date estimate for construction or repair of the ship and for this to set a TPQ for the last voyage and wrecking of the ship.

The challenge was to identify suitable wood samples for ^14^C dating. The ship remains as displayed, and most wood elements available, were treated with PEG (the standard conservation strategy for wet wood especially at the relevant time the Kyrenia Ship remains were conserved, see e.g. [25]). Since PEG contains petroleum products—introducing ‘dead’ ^14^C into the wood—any wood treated with PEG is unsuitable for ^14^C dating, since it will yield ^14^C ages that are millennia too old. All PEG contamination needs to be removed from a sample to prevent an erroneous much older ^14^C age. In fact, at least 99% of any dead carbon petroleum product contamination needs to be removed to produce dating results even close to the correct age, as observed for example by [26] at p.727. Despite some claimed successes that PEG and some other conservation treatments can be more or less successfully removed from wood samples for ^14^C dating (e.g. [26, 27]), much experience indicates problems in most past work (for a detailed study, see e.g. [28]). An initial attempt to date wood sample KYR-8 from the Kyrenia Ship in 2014 (see next section, KYR-8, below) provides an illustration of this problem using the range of then standard approaches. Application of a subsequently developed cleaning process (in 2022) has, however, permitted approximate use of this sample.

### KYR-8

KYR-8 is a pine (*Pinus nigra*) sample (M12) from bow sheathing (or repair) of the ship and has a reasonably long tree-ring sequence. The sample contained a total of 136 tree-rings (Relative Years, RY, 999–1034) with rings 1001–1131 measured. There is no extant evidence for outermost tree-rings (including bark or waney edge); thus the final extant tree-ring (RY1134) offers only a TPQ for the felling of the relevant tree and for the construction or a repair of the Kyrenia Ship. Since *Pinus nigra* is a potentially very long-lived tree, and the outer tree-rings can be very narrow, even missing a centimeter, let alone more, from the original cross-section, could easily mean that several decades or even a century or more of time are potentially missing and the ‘post’ in the TPQ could therefore be substantial. In an initial investigation (^14^C dates measured in 2014), 10-year samples were cut on 11 contiguous samples from RY1021-1030 to RY1121-1130 and ^14^C dated at the Oxford Radiocarbon Accelerator Unit, ORAU (Table 1). These form a tree-ring defined time-series of ^14^C ages for the mid-point of each 10-year sample with each 10-years (tree-rings) apart.

**Table 1:** ^14^C dates for the Kyrenia Ship. The gray-shaded data are not used in the analyses reported in this paper. The dates marked * are not employed as they clearly are either contaminated with PEG or are old wood (see main text). The two dates marked § have very low carbon content (<9% C at 8.9% for GrM-30709, and 5.5% for GrM-30714) and GrM-30714 also has a rather different (anomalous) δ^13^C value of -26.69‰ whereas all GrM samples on the same wood lie between -21.22‰ to 22.35‰. Thus these two samples are not used. References are given for ^14^C dates previously published: *R*13 [30] Lawn (1971); *R*24 = [31] Burleigh et al. (1982); *R*29 = [32] Ambers et al. (1987). These dates are not employed in the analysis. The other dates are published here for the first time. X dates from the ORAU are dates not given an OxA code and regarded as potentially suspect because of issues recognized in the pretreatment and dating process. We do not use them in the analysis. It is impossible to distinguish *Pinus brutia* from *Pinus halepensis* based solely on wood anatomy [33], so BM-1639R is best regarded as either *Pinus halepensis* or *Pinus brutia*. Clearly there is something amiss with the δ^13^C measurement reported for P-1621 –unless this is simply a typo (for e.g. -26.48‰). If this stated implausible δ^13^C value was however employed in calculating the reported ^14^C age, then the originally measured ^14^C age was even more recent [34] and so further discordant from the other dates on the almond samples, and this uncertainty over information renders this date suspect. OxA δ^13^C values ±0.3‰; Groningen δ^13^C values ±0.15‰.

Lab ID	Material	δ^13^C‰	^14^C Age BP	Reference
P-1621	Almonds (*Prunus dulcis*)	-6.48	2124±60	[30] at pp.363-364
P-1622	Wood from hull of ship	N/A	2222±43	[30] at pp.363-364
BM-1588	Almond shell (*Prunus dulcis*)	-26.9	2210±40	[31] at pp.239-240
BM-1588A	Almond shells (*Prunus dulcis*)	-26.9	2205±70	[31] at pp.239-240
BM-1639R	Wood (Aleppo pine: *Pinus halepensis*)	-24.7	2780±100*	[31] at pp.239-240 + [29] at p.72
BM-2294R	Pitch	-24.8	2390±120	[32] at p.68 + [29] at p.72
OxA-X-2561-15	Almond shell (*Prunus dulcis*)	-25.49	2337±27	This paper
OxA-X-2614-13	Almond shell (*Prunus dulcis*)	-23.14	2470±100	This paper
OxA-29333	Almond shell (*Prunus dulcis)*	-27.40	2214±25	This paper
OxA-29334	Almond shell (*Prunus dulcis*)	-26.57	2263±25	This paper
OxA-29335	Almond shell (*Prunus dulcis*)	-25.53	2274±26	This paper
OxA-29379	KYR-8 RY1021-1030	-23.71	2726±28*	This paper
OxA-29380	KYR-8 RY1041-1050	-24.02	2788±28*	This paper
OxA-29381	KYR-8 RY1051-1060	-24.08	2723±29*	This paper
OxA-29406	KYR-8 RY1091-1100	-23.84	2748±23*	This paper
OxA-29407	KYR-8 RY1101-1110	-23.95	2688±23*	This paper
OxA-29470	KYR-8 RY1061-1070	-24.02	2741±25*	This paper
OxA-29471	KYR-8 RY1071-1080	-24.26	2716±26*	This paper
OxA-29472	KYR-8 RY1081-1090	-23.56	2698±26*	This paper
OxA-29473	KYR-8 RY1111-1120	-24.35	2649±25*	This paper
OxA-29474	KYR-8 RY1121-1130	-23.77	2708±25*	This paper
OxA-29680	KYR-8 RY1031-1040	-23.21	2718±28*	This paper
GrM-30706	KYR-8 RY1000-1004	-21.61	2570±40	This paper
GrM-30707	KYR-8 RY1005-1009	-21.48	2488±40	This paper
GrM-30708	KYR-8 RY1023-1027	-22.35	2520±40	This paper
GrM-30709	KYR-8 RY1043-1047	-21.98	2700±50§	This paper
GrM-30711	KYR-8 RY1063-1067	-22.15	2465±40	This paper
GrM-30713	KYR-8 RY1083-1087	-22.35	2510±40	This paper
GrM-30714	KYR-8 RY1104-1107	-26.69	2330±80§	This paper
GrM-30716	KYR-8 RY1123-1127	-21.22	2450±40	This paper
OxA-30952	Almond shell (*Prunus dulcis*) W70-B	-23.12	2289±31	This paper
OxA-30953	Almond shell (*Prunus dulcis*)	-21.67	2343±29	This paper
OxA-31032	Wood twigs KS 5 USFP Jodrell J.10, W69	-26.85	2307±30	This paper
OxA-31033	Almond shell (*Prunus dulcis*) W70-B	-23.76	2329±30	This paper
OxA-31034	Almond shell (*Prunus dulcis*) W70-B	-25.51	2291±29	This paper
OxA-31677	KYR-35 RY1001-1005	-23.52	2345±27	This paper
OxA-31699	KYR-35 RY1001-1005	-23.74	2351±30	This paper
OxA-31700	KYR-35 RY1006-1014	-22.81	2214±21	This paper
OxA-31701	Tree Nail from C, “Jodrell 30”, KS1 (*Pinus brutia/halepensis*)	-23.10	2223±21	This paper
OxA-31840	KYR-35 RY1015-1019	-24.07	2200±21	This paper
OxA-31841	KYR-35 RY1021-1025	-24.09	2215±19	This paper
OxA-31842	Astragalus W45 (*Ovis/Capra*)	-21.14	2267±19	This paper
OxA-31875	KYR-35 RY1015-1019	-22.52	2231±21	This paper
OxA-31876	KYR-35 RY1021-1025	-22.59	2184±33	This paper

In principle, this should have led to a straightforward tree-ring-^14^C-wiggle-match. The ORAU tried as thorough a pretreatment (solvent-based) as available at the time (2014) to remove PEG from the wood cellulose. These dates only demonstrated that there was still at least some small amount of PEG remaining in this wood even after pretreatment: they are centuries (perhaps on average ca. 250 ^14^C years) too old (see Fig 1). A previously published routine (non-Accelerator Mass Spectrometry, AMS) ^14^C date, BM-1639R [29] at p.72, reported on wood from the Kyrenia Ship, is similarly far too old (whether as wood from inner rings of an old tree, or re-used old wood, or because of conservation treatment also). These data may be excluded as not directly relevant to dating the Kyrenia Ship.

A subsequent series of AMS ^14^C measurements was therefore carried out on KYR-8 samples at the Groningen Centre for Isotope Research in 2022 (GrM samples in Table 1) following the pretreatment methods as described in [35]. This involved soaking the samples in hot (80°C) ultrapure water for 36 hours (shown to be the most effective means to remove PEG) before pretreatment to α-cellulose using the usual Groningen pretreatment [36]. Whereas 6 of the 8 GrM samples produced >18% carbon (see % C values in Fig 1), two samples produced very low amounts of carbon <9% (GrM-30709 with 8.9% and GrM-30714 with 5.5%), rendering them suspect, and GrM-30714 also produced an anomalous δ^13^C value of -26.69‰ whereas all the other GrM samples on the same wood lie between -21.22‰ to 22.35‰. Thus these two samples (GrM-30709 and GrM-30714) are not used in the analyses. The ^14^C dates obtained on the other six Groningen samples appear to indicate ages with most PEG removed (Fig 1): see further below.

### KYR-35 and other non-PEG treated wood samples and long-lived sample material

A few wood samples are available from the Kyrenia Ship which were never treated with PEG. KYR-35 is a small wood fragment of such non-treated material. It is a pine (*Pinus nigra*) sample from the stern post that had been left to dry out slowly. The sample was highly problematic in dendrochronological terms: it was very twisted/distorted, making efforts to study, measure and dissect it extremely difficult. Some material, i.e. tree-rings, may have been lost from desiccation. There were 30 tree-rings observed and no outermost rings—thus the last extant tree-ring sets only a TPQ. However, it was evident we did not have a satisfactory and complete tree-ring sequence, and a number of likely original rings were missing or unable to be observed or coordinated in the extant sample. We might especially suspect problems in the older (earlier) part of the sequence where a number of rings may be missing.

Sample KYR-35 was dissected into a time series of 4 sections and AMS ^14^C dated at the ORAU (OxA dates) for a short tree-ring-^14^C-wiggle-match exercise, comprising (approximately) what were defined as Relative Years (RY) 1001–1005 (midpoint 1003), RY 1006–1014 (midpoint RY1010), RY1015-1019 (midpoint RY1017) and RY1021-1025 (midpoint RY1023). However, as just stated, these definitions should be seen as extremely elastic, especially for the dates on the innermost tree-rings. Two ^14^C dates were run on the first, third and fourth sections and one date on the second section (see Table 1). But, while we believe the series order is correct (oldest to most recent), observation of the sample leads us to treat the ring identifications as only very approximate, especially for the first half of the sample, and so we apply an uncertainty allowance on the calendar (tree-ring) information of up to ca. 200% for the first half of the series, and about 50% for the remainder of the series. At the same time, as a small sample, the total temporal length involved in the sample’s tree-rings cannot be too long (e.g. probably less than ca. 60 years in total). A wooden tree-nail (C Jodrell 30 KS1) of pine (*Pinus brutia/halepensis*) (OxA-31701), placed as relevant to ship construction/repair, and a sample comprising a wood twig (KS5 USFP Jodrell J.10 now W69) (OxA-31032), placed as relevant to the cargo/contents and last voyage, were also AMS ^14^C dated at ORAU. In addition, there are some previously reported routine (non-AMS) ^14^C dates run around 50 years ago. P-1622 is on non-treated wood from the hull [30] (see Table 1). This date did not receive NaOH pretreatment nor δ^13^C measurement and correction and so must be viewed as less than accurate versus modern properly pretreated and analyzed AMS ^14^C dates. Another previously reported date, BM-1639R on pine, is very old and similar to the ORAU dates on the PEG affected samples of KYR-8. Whether due to PEG contamination or because it is very old or re-used wood, this BM age is clearly not relevant to the date of the ship and so we exclude it. A final sample in the long-lived category is the previously published BM-2294R [29] at p.72 on pitch material (see Table 1).

### Other dated short/shorter-lived organic samples

Short- or shorter-lived sample material was found with the wreck, presumably from cargo or ship crew use, and is thus likely to relate to the last voyage of the ship. Unless residual, these samples should yield ^14^C ages relatively close to, or within a year to a few years of, their use. None of these samples had ever received conservation treatment and thus should be free of modern contaminants. This material was AMS ^14^C dated at the ORAU. There are also a few previously published routine (non-AMS) dates on similar samples. The majority of these dates on short-lived sample matter are on elements of a cache of almonds (*Prunus dulcis*) found with the wreck, consisting of: a Pennsylvania Laboratory date (P-1621 in [30] at pp.363-364), two British Museum dates (BM-1588, BM-1588A in [31] at pp. 239–240), and seven of the new OxA dates reported here (as well as two OxA-X dates): see Table 1. We observe that the δ^13^C value reported for P-1621, -6.48‰, makes no sense considering the expected value range for this species. All the other δ^13^C values reported for the almond samples are within a plausible range (from ca. -21.7‰ to -27.4‰) (and, as an example, [37] at Table 1 reports a mean value of -26.43‰ for *Prunus dulcis* from a Mediterranean case study). This leaves the P-1621 date as suspect (unless it is a typo and was meant for instance to be -26.48‰?) and it is noticeably more recent than any of the other dates obtained on almond samples. The OxA-X designation from the ORAU (two dates on almond samples in Table 1) reflects where either the analytical data associated with the measurement is outside the expected range, or where there is an experimental pretreatment applied. The advice is to regard such samples with caution. In the case of OxA-X-2561-15, it was also noted that the sample produced a much lower than expected yield of carbon upon combustion (18% versus a normal range of 40–50%), while OxA-X-2614-13 exhibited both a low carbon yield and had a low target current. We thus do not employ these two X dates in our analysis, nor the P-1621 date (apart from illustrating that it is very different in one figure below).

In addition to the almond samples, we dated an animal astragalus (W45) (*Ovis/Capra*) and an unidentified twig, mentioned above (KS5 USFP Jodrell J.10 now W69, OxA-31032). These two samples should also offer shorter-lived samples; although the astragalus could have been in use (e.g. for gaming or divination [38]) for a period of time. For the ^14^C dates, see Table 1.

### ^14^C dating

The ^14^C determinations on Kyrenia Ship samples published here for the first time comprise AMS ^14^C samples measured at the ORAU (OxA) and Groningen (GrM): Table 1. The dates are corrected for isotopic fractionation using the δ^13^C values measured in the AMS—whereas the quoted δ^13^C values in Table 1 were measured independently on a stable isotope mass spectrometer (±0.3‰ relative to VPDB for OxA and ±0.15‰ for GrM). Details of methods at the ORAU for chemical pretreatment, target preparation, and AMS measurement follow those set out in [39–42]. The laboratory procedures at Groningen are as set out in [36]. In view of apparent issues with several of the previously reported routine ^14^C ages (run 50 years ago), their larger measurement errors, and likely issues of accuracy (versus modern AMS ^14^C dates), we focus here on the AMS ^14^C dates in our analysis.

Calibration of ^14^C ages into calendar year probabilities, Bayesian chronological modelling (e.g. [43, 44]) of the data integrating the prior information available from the samples and/or their type and context, and outlier assessment and treatment, employed the OxCal software [43, 45] version 4.4.4 and initially the current standard Northern Hemisphere IntCal20 ^14^C calibration curve [10] with curve resolution set at 1 year—but see below. OxCal command terms are capitalized in the text, e.g. Sequence, Phase, Boundary. Only models with Convergence values for all elements ≥95 were employed (where appropriate, the kIterations value—the number of Markov chain Monte-Carlo (MCMC) passes in the analysis—was increased from the default value of 30 by a factor of 100 to kIterations = 3000 to help ensure good Convergence). It should be noted that there are small variations in quoted details between different model runs; typical results from several model runs are reported.

### ^14^C calibration and issues with IntCal20 in the relevant period

**Fig 2:**
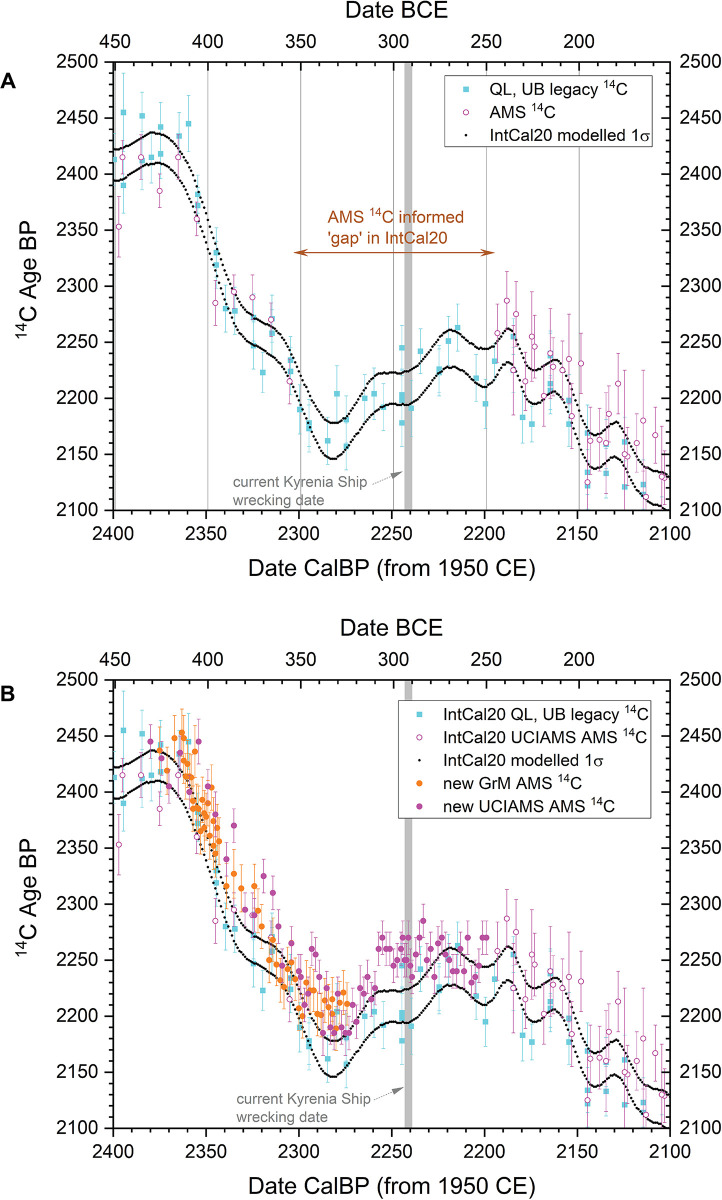
Existing and new ^14^C data for calibration curve construction 450–150 BCE. A. the constituent (raw) ^14^C data used to construct the IntCal20 ^14^C calibration curve [10] in the period from 450–150 BCE shown against the modelled 1σ IntCal20 curve and versus the date estimate for the Kyrenia Ship, 294–290 BCE, in [1]. There are no AMS ^14^C data between 350–250 BCE. B. The data in A. but adding the new AMS ^14^C data on known-age wood discussed and reported in this paper (see S1 Table). 1σ error bars are shown.

Preliminary analysis of the Kyrenia Ship ^14^C dataset revealed that the current standard ^14^C calibration curve, IntCal20, appears to introduce an additional complication for producing accurate and precise calendrical dating estimates. The relevant 350–250 BCE portion of the calibration curve in IntCal20 for dating the Kyrenia Ship materials is based primarily on relatively few legacy pre-AMS ^14^C data (and these on multi-year blocks of wood), versus recent AMS ^14^C information from samples comprising single (or very few) years: Fig 2A. There are only 22 data points for the period around 350–250 BC in IntCal20 and these data also comprise solely older, pre-AMS ^14^C values (from the University of Washington, QL, and Queen’s University Belfast, UB, datasets run several decades ago: e.g. [46]). These data were also run on multi-year (decadal or even bi-decadal) blocks of tree-rings and thus may miss fine (annual-scale) features of the ^14^C record. Thus the definition of the Northern Hemisphere atmospheric ^14^C record represented in IntCal20 across this time period is poorly delineated. Moreover, since investigations of other periods have in several cases found that revisions are necessary to such legacy ^14^C calibration data, and that such revisions, where applicable, have usually produced older ^14^C values or additional fine curve structure (e.g. [11–13, 47–50]), the circumstances combine to suggest that the current IntCal20 record 350–250 BCE may not offer an accurate calibration record for the Kyrenia Ship dataset.

Therefore, we use and report measurements on known-age (dendrochronologically dated) tree-ring samples for this period which better inform and revise the ^14^C calibration record, shown in Fig 2B (these ^14^C data and associated tree-ring information are listed and described in S1 Table, S1 Fig and S1 File). The Groningen (GrM) data on known-age annual tree-rings (deciduous *Quercus* sp.) are those reported with full details elsewhere [14]. The KECK Carbon Cycle AMS Facility, University of California, Irvine (UCIAMS) data are newly reported measurements on known-age annual *Sequoiadendron giganteum* tree-rings. For initial holocellulose production the sample pretreatment methods described in [50] were followed; then, for the samples reported here, an additional step was added to generate alpha-cellulose: before drying, holocellulose samples were treated at room temperature with 5N NaOH (1 hour, repeated until the base solution remained clear), 1N HCl (70°C, 30 minutes) and ultrapure MQ water (70°C, 30 minutes, repeated until pH >4).

**Fig 3:**
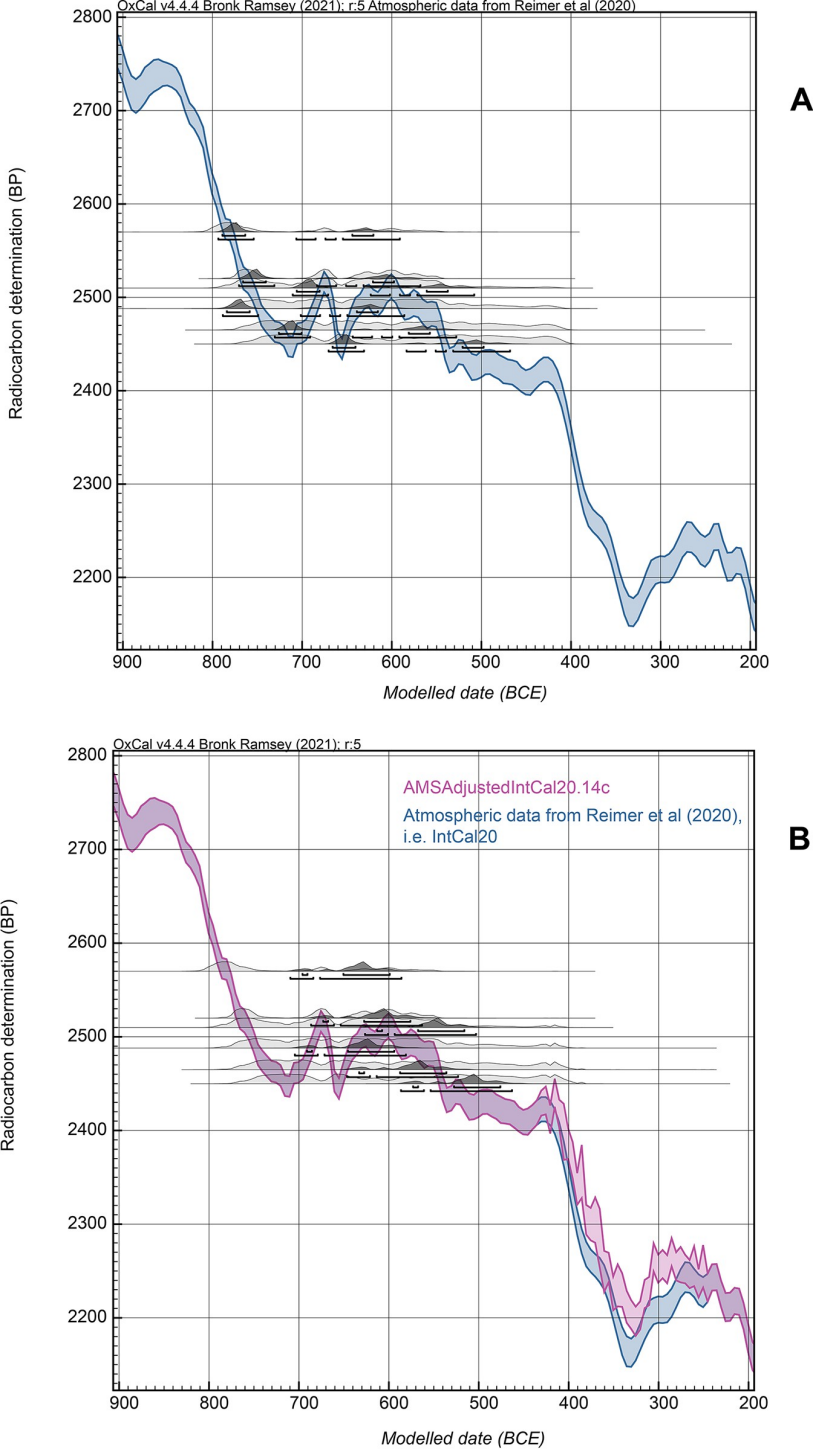
The wiggle-match fit of the tree-ring defined time-series of GrM AMS ^14^C dates on the KYR-8 timber (Table 1). A. The data after pretreatment that should have removed nearly all (most) of the PEG contaminant (as confirmed by the known-age PEG test shown in Fig 6 below) versus IntCal20 [10] (resolution 5 years). B. The data in A. but with the last extant year, RY1134, placed with uniform probability between 600–250 BCE and using the modified new AMS ^14^C dataset (with resolution set at 5 years): referred to as the ‘AMSAdjustedIntCal20’ calibration curve. This last model run offers more recent age ranges but still places the series before the steep slope (change) in atmospheric ^14^C around 400 BCE. Data from OxCal [43] version 4.4.4. The upper and lower lines under each distribution indicate, respectively, the 68.3% and 95.4% highest posterior density (hpd) ranges.

It is evident that the new data offer a revision to the previous IntCal20 dataset. The substantial change in slope at the end of the 5^th^ century BCE is still evident (as evident also from the dates reported recently on the Mazotos ship: [15]), but the AMS ^14^C values in general are a little older (in ^14^C terms) than the IntCal20 curve (tending to shift calibrated calendar ages to slightly more recent ages). The new values also reduce/modify the previous strong dip in ^14^C values 350–310 BCE and raise the calibration curve in the period around and following 300 BCE. This suggests that slightly different calendar age probabilities will exist using these new calibration data 400–250 BCE. For this paper we therefore compare results from IntCal20 versus those from a modified version of IntCal20 that uses the new data shown in Fig 2B (see the indicated modified curve section and versus unmodified IntCal20 in Fig 3). The curve shown in Fig 3B, and used below, is a simple linear interpolation at 5 calendar year resolution using OxCal (and also compared with a cubic extrapolation below). This is an interim step pending the next iteration of IntCal which will include these and other new datasets applying much more sophisticated modelling (e.g. [51]).

**Fig 4:**
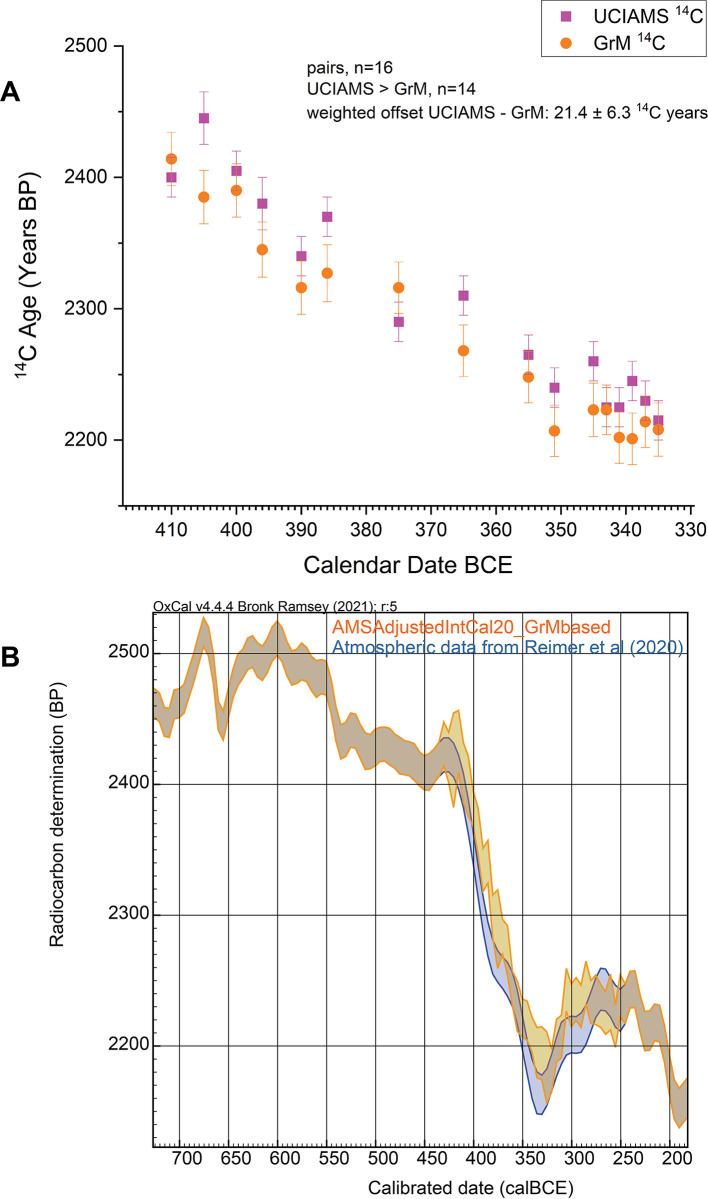
A. ^14^C data from the same calendar year comparing values from UCIAMS on sequoia versus GrM on European oak and B. comparison of an adjusted calibration curve with all values adjusted based on the GrM European oak data values shown versus IntCal20 (contrast Fig 3B). Data in A. shown with 1σ error bars; calibration curves in B. shown as 1σ bands—with data in B. from OxCal [43] version 4.4.4.

One further issue is evident examining the new GrM and UCIAMS data. In 16 cases a single-year tree-ring representing the same calendar year was measured on oak at Groningen and on sequoia at the University of California, Irvine. These specific pairs are shown in Fig 4A. It is apparent that the ^14^C ages from UCIAMS on sequoia are typically older than the GrM ages from European oak (14 of 16 instances). In a number of cases the two measurements are not consistent with representing the same ^14^C age as reported (using ref. [52]). The weighted average difference (UCIAMS-GrM) is 21.4±6.3 ^14^C years [52]. Some of this difference may result from the following factors:

(i) Differences in growing season for the sequoia versus temperate-boreal trees like European oak (thus capturing a slightly different reflection of the annual atmospheric ^14^C cycle [53]). In particular, the overall longer growing season for the sequoia, and its generally reduced growth and sensitivity to drier summer conditions [54, 55], likely minimizes representation of the late summer high in atmospheric ^14^C versus European oak data, whether on whole rings, and very especially where the ^14^C measurements are on oak latewood only as in [12];

(ii) Some of the difference may also represent a Pacific Ocean offset element since ^14^C dates on known age tree-rings from Japan have regularly shown a small older age trend (e.g. [56]);

(iii) Some of the difference may reflect a small inter-laboratory offset between UCIAMS and GrM.

Without a much larger and targeted study we cannot discriminate and identify the sources of the differences (and this was not the focus of the present investigation). However, the relevant point for this study is that the GrM values indicate less of a difference versus IntCal20, and especially in the earlier 3^rd^ century BCE (see Fig 2B).

We might therefore suggest that the ‘AMSAdjustedIntCal20’ curve shown in Fig 3B, using both the UCIAMS and GrM data, is probably about a maximum change scenario versus the current IntCal20 calibration curve. If we adjust all the UCIAMS data by 21.4±6.3 ^14^C years to better match the GrM data on European oak, and derive an ‘AMSAdjustedIntCal20_GrMbased’ calibrated curve (see Fig 4B), then this likely represents about the minimum required adjustment to the current IntCal20 calibration curve. In each case, with modern AMS ^14^C measurements, it is clear that adjustment to slightly older ^14^C ages, and hence slightly more recent calendar ages, are required across the period 400–250 BCE.

In this paper we primarily use the ‘AMSAdjustedIntCal20’ curve shown in Fig 3B, incorporating all our new calibration data, especially as we do not know whether the samples in the Mediterranean case at issue here more likely reflect the sequoia (and its more ‘Mediterranean’-like climate-growth pattern), or temperate-boreal oak (etc.), calibration records, and hence a combined calibration curve appears to offer a best estimate and one likely including a Mediterranean-relevant atmospheric signal. In some cases we then also compare with the ‘AMSAdjustedIntCal20_GrMbased’ calibrated curve (see Fig 4B).

### ^14^C dating offsets and considerations for sample KYR-8

Other potential factors affecting accurate dating of the Kyrenia samples are two types offsets to the ^14^C dates related to (i) growing season and (ii) presence of residual PEG in the samples.

Growing season related ^14^C offsets have been observed as relevant in dating some archaeological material in the Mediterranean [13, 57, 58]. However, in this case, it is likely largely irrelevant for most of the Kyrenia Ship samples. *Pinus nigra* is a higher elevation tree species and thus has a growing season, spring through summer to start autumn, largely similar to the trees supplying the current IntCal dataset ([59] at p.79), while *Prunus dulcis* are typically (unless eaten green) harvested in the later summer and again their growth thus also parallels the period represented by the standard IntCal samples ([59] at p.197). This at most leaves a few other samples for whom a small growing season offset might apply, and the topic is thus unlikely to be of substantive relevance in this case.

The second potential ^14^C offset in the Kyrenia Ship dataset relates to the effects of residual PEG in the sample material (KYR-8). This is clearly relevant, as a major offset for the OxA data and as a (likely more minor offset) in the GrM data (Fig 1). Examining both the OxA and GrM dates on the KYR-8 samples illustrates one common key pattern: the two tree-ring time series, whether (i) the OxA series with remaining PEG, or (ii) the GrM series with the PEG removed or largely removed, in each case show ^14^C ages that across each of their respective time-series remain relatively similar (with the OxA series offset to substantially older ^14^C ages due to remaining PEG contamination) and so likely describe a century-scale series running across a plateau in the ^14^C calibration curve (Fig 1). It is highly unlikely these series of dates match with a period with rapidly changing ^14^C levels (a steep slope), nor a period of any major ‘wiggle’ or reversal in atmospheric ^14^C values. In the period between 800 to 250 BCE—the plausible extreme chronological limits within which this wood must lie—this means the samples are very likely to represent tree-rings that formed during the time of the so-called Hallstatt Plateau in the ^14^C calibration curve; that is, sometime from the later 9^th^ through the end of the 5^th^ centuries BCE. They cannot plausibly describe the period 400–250 BCE. At the same time, the extensive material culture evidence associated with the Kyrenia Ship would indicate that, even if KYR-8 were to be missing 50, 100 or even 200 years of the outermost tree-rings, the last extant tree ring (RY1134) should date somewhere more in the region of ca. 500–300 BC. Hence when we consider a wiggle-match (Fig 3), the last dated element in the wiggle-match (placed as RY1125, just 9 years earlier) should be somewhere ca. 509–309 BC and the first dated element (RY1002) no older than ca. 632–432 BC.

To control for PEG offsets, we employ only the GrM dates for KYR-8, as it is evident the OxA dates on KYR-8 samples are much too old and retain substantial PEG aging effects (see Fig 1). If we use the tree-ring informed sequence and wiggle-match the GrM dates (against IntCal20), they are placed across the later Hallstatt Plateau (Fig 3A). But, despite the best efforts at PEG removal, it appears that there is still a small offset to older (and thus minimally-PEG-affected) results, although a small portion of this offset may also be the difference between modern AMS ^14^C dates versus the legacy ^14^C dates informing some parts of this section of the IntCal20 calibration curve. The RY1125 sample is placed somewhere between 671 to 469 BC (at 95.4% hpd) and the RY1002 sample between 794 to 592 BC (at 95.4% hpd). Both ranges seem too old by a century or more and far too wide (due to the Hallstatt Plateau). It would therefore seem reasonable to limit the date range for the placement of the end of the series (RY1134) as plausible only between 600–250 BC (uniform probability).

We may also consider the possible relevance of adjusting the IntCal20 calibration curve with the new calibration data shown in Fig 2B. The revised wiggle-match and the revised calibration curve are shown in Fig 3B. The wiggle-match is moved to a more recent range but still appears too old. At 95.4% hpd the RY1002 element is now placed between 710–586 BC but with a poor OxCal Agreement (A) value (36.1 < 60), as it sits rather older than the calibration curve, and the RY1125 element is between 587–463 BC. Thus it seems there is likely some, probably very small, remaining PEG contaminant factor at least in some samples, as well as probably a small AMS ^14^C to legacy ^14^C issue for the portion of the Hallstatt Plateau still largely determined from legacy ^14^C data.

We might therefore consider employing a reasonable remaining offset allowance, and examine what difference such an additional offset allowance potentially makes to the calendar placement of KYR-8. In a post-cleaning case like this, there is no reason to expect that any remaining offset will be consistent between the samples. Various factors might lead to varying situations, depending on the varying amounts of residual PEG fraction in the wood cells.

Since there is no objective way to assess the individual cases, the best strategy appears to assess what average offset—that is the same offset applied to each sample—would yield the best fit of data to the calibration curve and then to consider what effect this has on the calendar placement of KYR-8. Examination of Figs 1 and 3 would suggest that any remaining offset is likely in the range from 0 to at very most about 400 ^14^C years. Using the modified AMSAdjustedIntCal20 calibration curve, we thus considered OxCal Delta_R tests of 0±10, 0±25, 0±50, 0±75 … 0±300 ^14^C years as more than covering this range. We limited the placement of the last extant tree-ring, RY1134, at uniform probability to somewhere between 600–250 BC.

**Fig 5:**
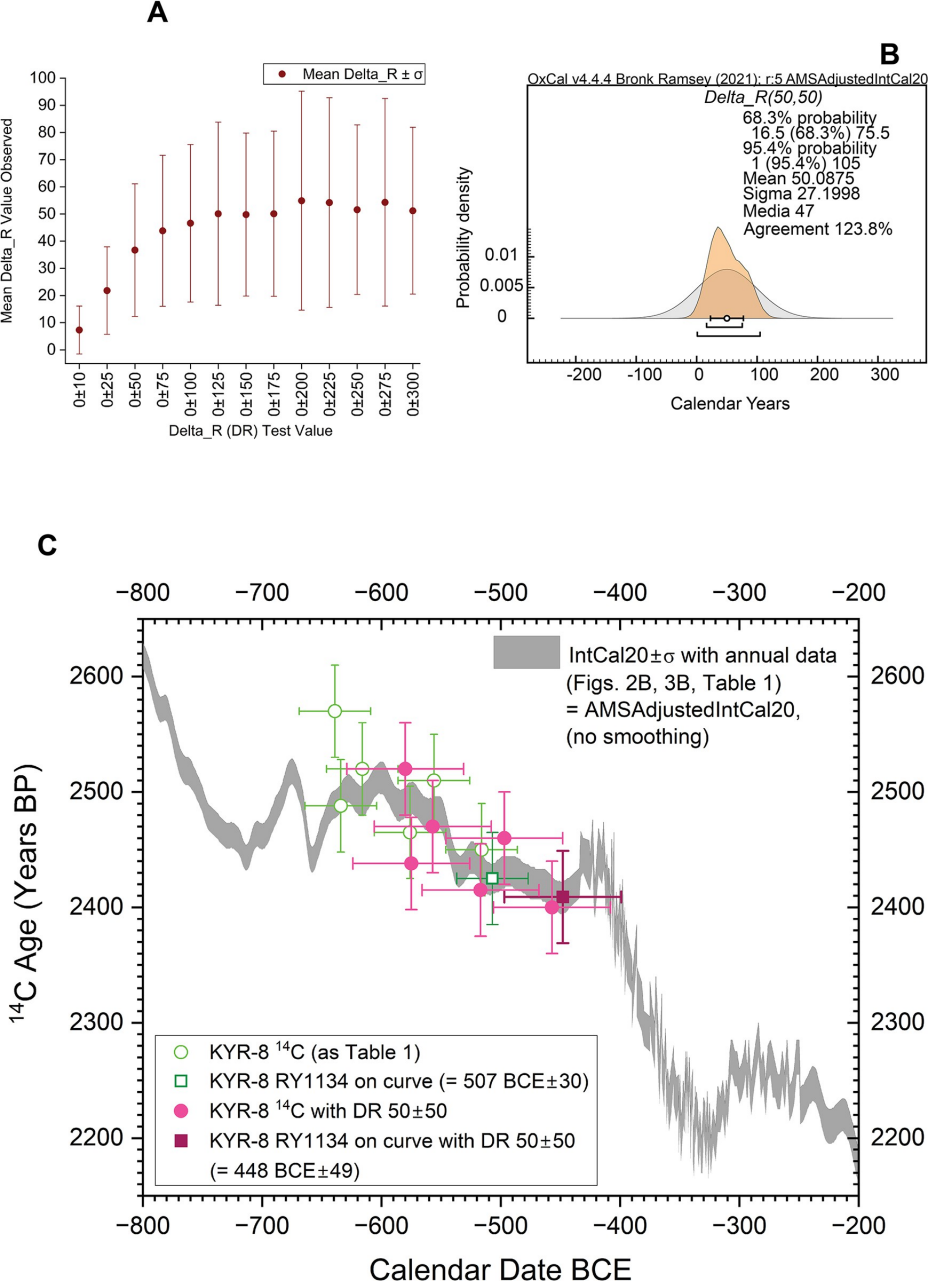
Likely range of remaining PEG-caused offset in the cleaned GrM KYR-8 samples. A. The Delta_R offset (μ±σ) identified from neutral Delta_R tests of 0±10, 0±25, 0±50 …0±300 ^14^C years applied to the KYR-8 wiggle-match series (against AMSAdjustedIntCal20). The offset stabilizes around 50±50 ^14^C years. B. A wiggle-match run with a generous Delta_R of 50±50 ^14^C years offers good agreement comfortably within assumption error margins between modelled and assumed offset. C. The μ±σ wiggle-match calendar placements of the KYR ^14^C ages (±σ) and modelled last extant ring (RY1134 ±σ) against the modified AMSAdjustedIntCal20 calibration curve (±σ) (Figs 2B and 3B) comparing (green) the KYR-8 data as in Table 1 with no additional Delta_R adjustment (but with RY1134 placed between 600–250 BC at uniform probability), and (pink) the data applying a generous Delta_R of 50±50 ^14^C years (and RY1134 placed between 600–250 BCE at uniform probability). Data from OxCal [43] version 4.4.4.

It is noticeable that the mean ± σ Delta_R values increase as the possible range increases, but then approximately stabilize (and different model runs yield very slightly varying values), with mean values from the 0±100 ^14^C years test onwards yielding values in the higher 40s and especially low 50s with σ values <41. An approximate Delta_R value of about 50±50 ^14^C years therefore generously captures the range of what appears as the plausible offset (Fig 5A). This conservative approximation offers a reasonable (if generously wide) fit of data and model assumptions (with a returned mean close to the model assumption) versus the calibration curve with an OxCal A value of ca. 124% (Fig 5B). Thus, although the level of remaining contaminant may well vary between the samples, this suggests that application of an ‘average’ additional offset of around 50±50 ^14^C years may likely yield (or generously include) about the correct ‘average’ ^14^C values for the series and so the approximate correct or latest plausible calendar age ranges for the wiggle-match. Fig 5C compares a no Delta_R fit, with RY1134 constrained between 600–250 BC with uniform probability, versus the Delta_R 50±50 ^14^C years fit with RY1134 between 600–250 BC with uniform probability. The 95.4% hpd calendar ranges BC for the last extant tree-ring of KYR-8, RY1134, are respectively (i) with no Delta_R: 578–553 BCE (12.9%), 545–530 BCE (6.2%), 526–454 BCE (76.3%), and (ii) with Delta_R 50±50 ^14^C years: 542–530 BCE (2.4%) and 523–358 BCE (93.1%).

Even with the modified calibration curve and with the generous Delta_R offset of 50±50 ^14^C years, the GrM KYR-8 data series remains clearly placed on the Hallstatt Plateau and does not continue beyond it (Fig 5C). Further, even at the limits of modelled 95.4% ranges and with the new, revised, AMS ^14^C dataset for this period (‘AMSAdjustedIntCal20’), the calendar position of the last extant tree-ring, RY1134, does not reach beyond 358 BCE (376 BCE as latest 68.3% hpd year). With IntCal20 (not adjusted) but using the Delta_R offset of 50±50 ^14^C years, these last possible years for estimating a minimum felling date for KYR-8 are 374 BCE at 95.4% hpd and 386 BCE at 68.3% hpd.

### No other soundly based dendrochronological calendar date information is available

Our finding of a 95.4% hpd date limit before ca. 374 BCE for RY1134 (IntCal20) or before 358 BCE (revised AMS ^14^C dataset) in fact implies that the likely real date for the last extant tree-ring is of course older than this latest possible date, e.g. before ca. 400 BCE: see Fig 5C. This finding is relevant to claims for a supposed dendrochronological date for the Kyrenia Ship hull timbers and is an important part of our explanation for why we do not employ this claimed information in our analysis. Kuniholm et al. [17] at Fig 1 show a Kyrenia Ship Hull series placed at ca. 490–330 BCE (and [8] at p. 281 refers to a forthcoming chapter by Kuniholm also alleging a 330 BCE end date for this sequence). This claim refers to the 147-year *P*. *nigra* sequence (plus one unmeasured partial ring) from the bow sheathing, including samples KYR-1+8, with KYR-8 providing the most recent (and so relevant) portion of this sequence. Kuniholm et al. [17] fail to provide any explanation, nor are supporting dendrochronological details provided, and there is no secure known-age same/similar species tree-ring chronology from the relevant geographic region and ecological zone to offer a plausible crossdate. The ^14^C data further disprove this claimed date. The supposed end date (for RY1134) is at least 44 or 28 years too recent to be potentially consistent within even the very latest limits of the 95.4% probability ranges from the ^14^C wiggle-match evidence from this same wood series even with a generous plausible offset adjustment allowing for any remnant PEG contamination. In fact, Kuniholm’s proposed placement is more realistically at least 69 years or 147 years too recent versus the lower 1σ limits of the μ±σ wiggle-match placements shown in Fig 5C versus the modified AMSAdjustedIntCal20 dataset, and against unmodified IntCal20, respectively.

The relevant observation is that the end point (last extant tree-ring) for the KYR-8 sample, RY1134, can reasonably be constrained as belonging *no earlier* than the later Hallstatt Plateau period (so a TPQ in the late 6^th^ century BCE) but also *no later* than about the point in time when the plateau ends and there is a sharp change in atmospheric ^14^C levels and thus the change in gradient to a steep slope in the ^14^C calibration curve from around ca. 410 BCE (and certainly before 374/358 BCE). Supposed dates for this wood many decades later can be dismissed as implausible. Nonetheless, for the purposes of modeling the overall Kyrenia Ship ^14^C dataset, we might reasonably apply a very conservative constraint that this last extant tree-ring (RY1134) in fact dates somewhere between 550–300 BCE.

### Known-age test on PEG removal

**Fig 6:**
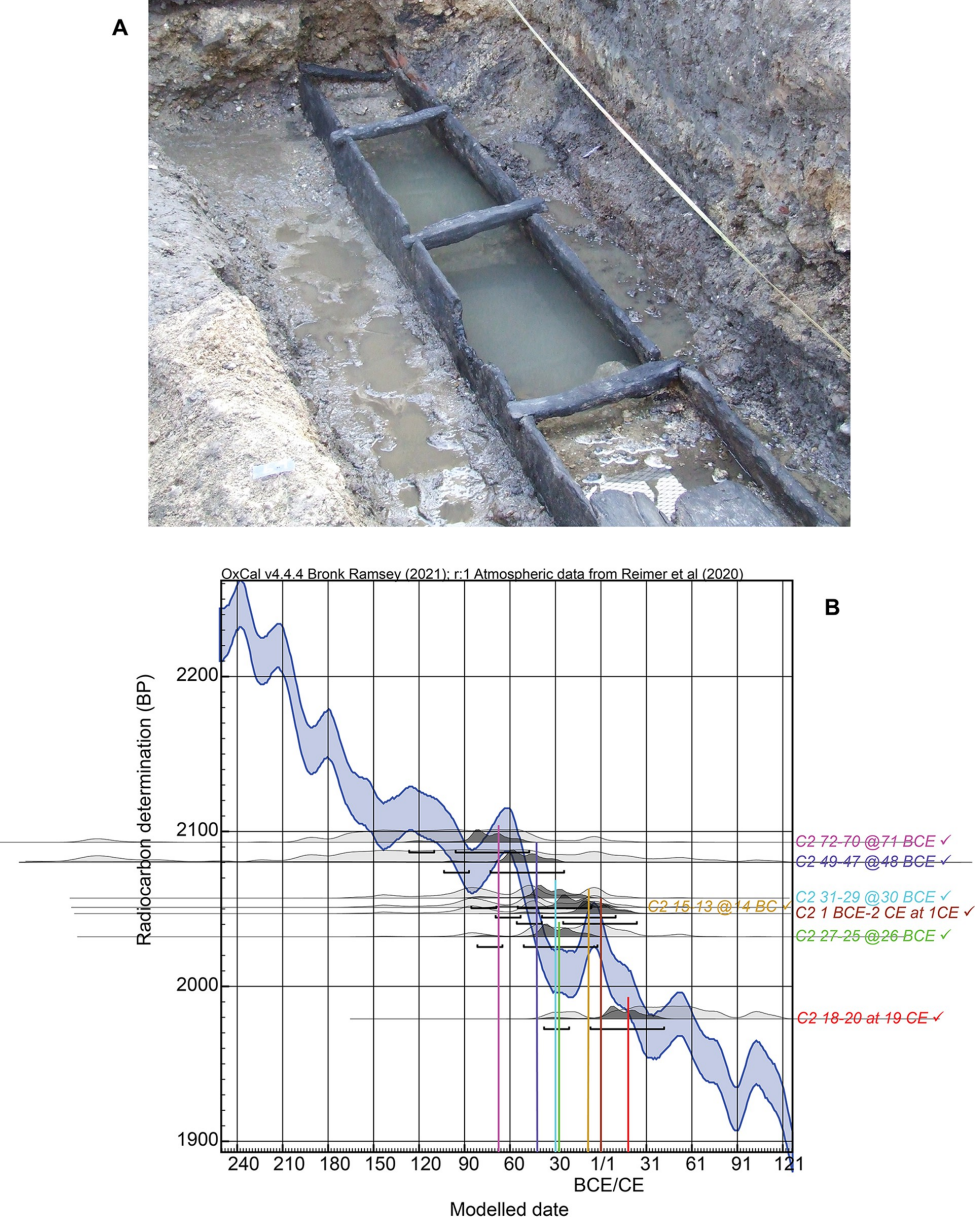
Test of PEG removal on a known-age tree-ring series. A. Oak (*Quercus* sp.) drain construction found in a rescue excavation by the Colchester Archaeological Trust in St. Peter’s Street in Colchester in February 2008 [60]. Sample C2 came from this drain construction and was PEG treated in 2008. Photo: Martin Bridge. B. Comparison of the wiggle-matched (known tree-ring series) ^14^C ages on tree-ring samples from sample C2, 95.4% hpd ranges are indicated, compared with the known calendar (dendrochronological dating) of these samples using IntCal20 [10]—illustrating good correspondence and thus successful removal of the PEG contaminant. Data from OxCal [43] version 4.4.4.

The GrM pretreatment applied to the KYR-8 samples was designed to try to remove the PEG contamination. It has proved successful in other cases (e.g. [35]). However, it appears important to conduct an independent known-age test and on a sample of broadly similar age (i.e. not relatively recent) to gauge success. We thus ^14^C dated and wiggle-match dated a series of 7 tree-ring segments (running from 72 BCE to 19 CE) across a dendrochronologically known-age dated wood (*Quercus* sp.) sample (C2) from a Roman period drain construction obtained in a rescue excavation by the Colchester Archaeological Trust in St. Peter’s Street in Colchester, UK, in February 2008 (Fig 6A) [60]. The archaeological context is placed before Boudica’s revolt in 60–61 CE and the oak wood was dendrochronologically dated against other English oak series by MB. This wood was impregnated with PEG as part of its conservation in 2008 and thus had remained as PEG-treated for 14 years by the time we attempted cleaning and ^14^C dating—hence offering a reasonable comparison for the Kyrenia Ship case and in contrast with ^14^C pretreatment tests where cleaning was attempted only shortly after treatment.

The ^14^C data obtained are listed in Table 2, and the calendar age probabilities from the tree-ring sequenced wiggle-match (listed in Table 2) are shown against the IntCal20 ^14^C calibration curve in Fig 6B. In all cases the 68.3% hpd wiggle-match ranges (and thus also the 95.4% hpd ranges) include the known-ages, indicating good success at removing the PEG contamination. Therefore, we may reasonably hope that the same pretreatment applied to the KYR-8 samples will have made them PEG free, or at least very nearly PEG-free. The Kyrenia Ship material was impregnated for rather longer, which may make it more difficult to completely remove all PEG. It is also possible that wood anatomical structures and cellular composition in different tree taxa (e.g., oak vs. pine) have small effects on PEG removal. Nonetheless, it is reasonable to regard the GrM KYR-8 results as likely close to PEG-free.

**Table 2:** ^14^C dates on the Colchester (C2) oak (*Quercus* sp.) sample for known-age PEG removal test. Unfortunately, a technical issue at the time meant that no reliable IRMS δ^13^C data are available. The known-age for each sample falls within the ^14^C wiggle-match 68.3% hpd range in all cases.

Lab ID	Material–all *Quercus* sp. (tree-rings dated)	^14^C Age BP	Wiggle-match 68.3% and 95.4% highest posterior density (hpd) ranges (start/end)
GrM-30717	Rings 72–70 BCE	2093±40	87–62 BCE (68.3%); 127–48 BCE (95.4%)
GrM-30718	Rings 49–47 BCE	2080±70	64–39 BCE (68.3%); 104–25 BCE (95.4%)
GrM-30095	Rings 31–29 BCE	2057±24	46–21 BCE (68.3%); 86–7 BCE (95.4%)
GrM-30096	Rings 27–25 BCE	2032±24	42–17 BCE (68.3%); 82–3 BCE (95.4%)
GrM-30097	Rings 15–13 BCE	2051±24	30–5 BCE (68.3%); 70 BCE-10 CE (95.4%)
GrM-30098	Rings 1 BCE-2 CE	2047±23	16 BCE-10 CE (68.3%); 56 BCE-24 CE (95.4%)
GrM-30099	Rings 18–20 CE	1979±23	3–28 CE (68.3%); 38 BCE-42 CE (95.4%)

### Dating model considerations for KYR-35

We have noted above that while the temporal order of the dated segments appears secure, the exact dendrochronological sequence for KYR-35 (not PEG treated) is only very approximate and there are undoubtedly a substantial number of missing or extra (i.e. unaccounted-for) tree rings, especially in the first (inner/older) part of the sample. The ^14^C ages obtained further inform on this situation. The oldest rings dated, RY1001-1005, were measured twice, and produced very similar ^14^C ages, 2345±27 ^14^C years BP and 2351±30 ^14^C years BP, which both point to calendar ages within the range ca. 413–388 BCE (68.3% range from IntCal20). The weighted average [52] is 2348±21 ^14^C years BP which gives a 68.3% hpd range from IntCal20 of 407–394 BCE, and the intercept is ca. 400 BCE. The date range with the new AMSAdjustedIntCal20 calibration curve ^14^C data set in Fig 3B is slightly later but similar: 406–370 BCE at 68.3% hpd, 464–437 (4.7%), 425–418 (1.0%) and 413–362 (89.8%) at 95.4% hpd. Since this segment was measured twice, both times achieving very similar ^14^C ages, it seems reasonable to assume this age range is approximately correct.

**Fig 7:**
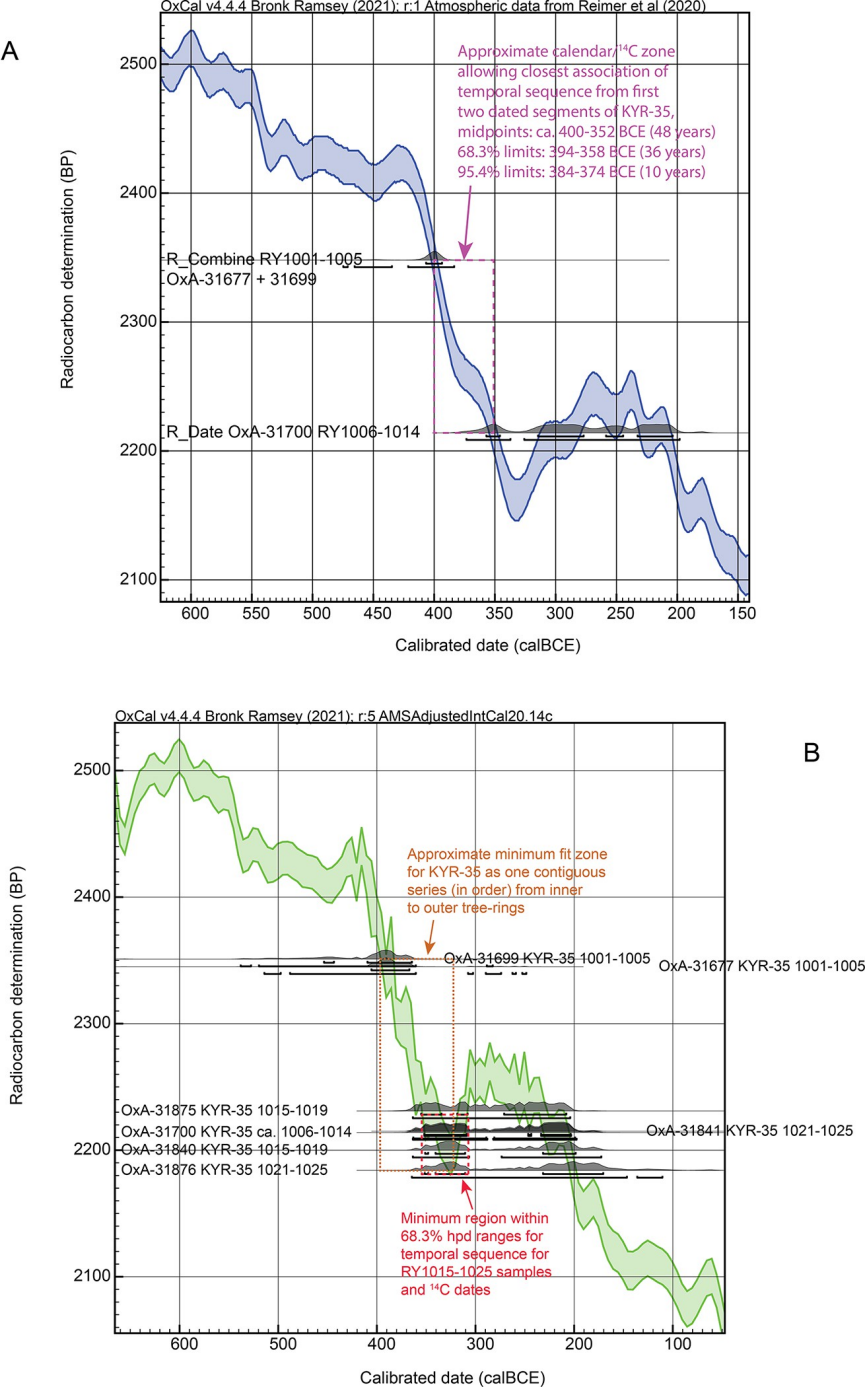
Calendar placement of the KYR-35 sample. A. Comparison and placement of the first two (arranged from inner/older to subsequent) dated tree-ring segments (the first a weighted average of two dates) from KYR-35 versus IntCal20 [10]. This identifies a specific fit zone in the earlier 4^th^ century BCE. B. Comparison and placement of all the dates on the ordered tree-ring samples from KYR-35 compared against the AMSAdjustedIntCal20 curve using the data reported in S1 Table (see Figs 2B and 3B). The approximate minimum fit zone that can accommodate all the dates and in the known order is indicated and covers the early to later 4^th^ century BCE. The upper and lower lines under the distributions indicate the 68.3% and 95.4% hpd ranges. Data from OxCal [43] version 4.4.4.

The other five dates on the KYR-35 segments all yield rather more recent ^14^C ages (more than 117 ^14^C years later): between 2231±21 ^14^C years BP and 2184±33 ^14^C years BP. The two cases here comprising pairs of dates also yield consistent results, suggesting again that these dates are approximately correct. In each case more than one tree-ring was combined for the measurement, so the ^14^C values are each an average of at least a few years minimizing any possible dramatic year-to-year excursions. In between the dates centered at the notional RY1003 and RY1010 placements, there is a 134 ^14^C years difference. Considering the central values versus those of IntCal20, the minimal calendar placements required to explain these results and difference are ca. 400 BCE and 352 BCE (48 years apart; ca.47 years between 396 BCE and 349 BCE considering the ‘AMSAdjustedIntCal20’ ^14^C revised dataset in Fig 3B), both falling in a period of rapidly changing atmospheric ^14^C levels (which corresponds with a steep slope in the ^14^C calibration curve, shown in Fig 7A). Allowing for the measurement errors this gap could be reduced a little (Fig 7A). This major ^14^C difference ties the earlier part of KYR-35 to the first half of the 4^th^ century BCE; it also implies that between notional RY1001-1014 in the fragmentary/distorted KYR-35 sample there were probably some 100–200% missing or unaccounted-for rings.

Critically, however, the ^14^C ages for the more recent segments of KYR-35 do not correspond to the wiggle to older ^14^C ages in the 3^rd^ century BC, but must lie on the section of the ^14^C calibration curve beforehand, e.g. ca. 350–300 BC. This is even more clear-cut when we consider the ‘AMSAdjustedIntCal20’ ^14^C dataset (Figs 2B and 3B), where the ^14^C ages from about 310 BCE through the mid-3^rd^ century BCE are raised above those in IntCal20. This necessarily leaves the ^14^C dates on the KYR-35 (notional) RY1015 to RY1025 samples tied to the period ca. 360–310 BCE and not later. This is consistent also with a placement not too much later than the dates on the KYR-35 RY1001-1005 samples (Fig 7B). These placements are compatible with the observation that the KYR-35 sample was somewhat more complete/satisfactory in its second (more recent) half, suggesting that the RY1015-RY1025 portion of the sample sequence probably did only occupy a decade or, even allowing a 100% error regarding unaccounted-for rings, perhaps a two/three-decade range (and not too much more). Further, overall, KYR-35 was a small sample and clearly did not represent a long total time period (e.g. less than maybe ca. 60 years in total).

It is therefore striking that, if the ordered sequence for the series of KYR-35 segments and ^14^C dates is retained, then they can all only be compatible with a placement ranging from the earlier through later 4^th^ century BCE (Fig 7B). We may therefore plausibly rule out the possible post-300 BCE probability regions for the KYR-35 ^14^C dates.

### Dating considerations for the short/shorter-lived samples from the ship

Excluding two OxA-X dates (see above), we have a set of 12 ^14^C dates on short/shorter-lived material from the Kyrenia Ship. These samples were on board when the ship sank. Most should relate to the period immediately or shortly before the last voyage, and, even if a few were residual or—perhaps in the case of the *Ovis/Capra* astragalus—in use for a few/several years, they are unlikely to be more than a few years to a decade (or so) older than the ship’s last voyage. The almonds could be derived all from one location and harvest, but could also plausibly be collected from more than one source and/or more than one year of harvest. Realistically, though, their dates should be from within at most 0 to about 2 years of the shipwreck date as edible goods being transported/traded.

The range of ^14^C dates on the almond samples is quite large. Even restricting comparison just to the same laboratory OxA data, they are not consistent with representing the same ^14^C age: T = 15.1>12.6 for df6 at the 5% level: [52]). The range of the OxA set is 2343±29 ^14^C years to 2214±25 ^14^C years, very similar to the range of dates encompassed by the KYR-35 sample. If we rule out possible factors related to laboratory noise, such a wide range of dates within a set that should at most represent a very few calendar years is best (and only) explained by the samples coming from a period of very rapid and major change in atmospheric ^14^C levels, such as (i) the steep slope in the calibration curve in the early to later-4^th^ century BCE, or (ii) immediately after this on the steep reversal at the end of the 4^th^ century BCE and into the early 3^rd^ century BCE. It is notable that the ‘AMSAdjustedIntCal20’ ^14^C dataset highlights a rapid and major reversal generally between 324 BCE and 284 BCE changing from 2185±15 ^14^C years BP to 2285±15 ^14^C years BP, potentially 70–130 ^14^C years at 1σ (Figs 2B, 3B and 7B), and very specifically between 324 BCE and 307 BCE changing from 2185±15 ^14^C years BP to 2270±25 ^14^C years BP, potentially 45–125 ^14^C years at 1σ (Figs 2B, 3B and 7B).

**Fig 8:**
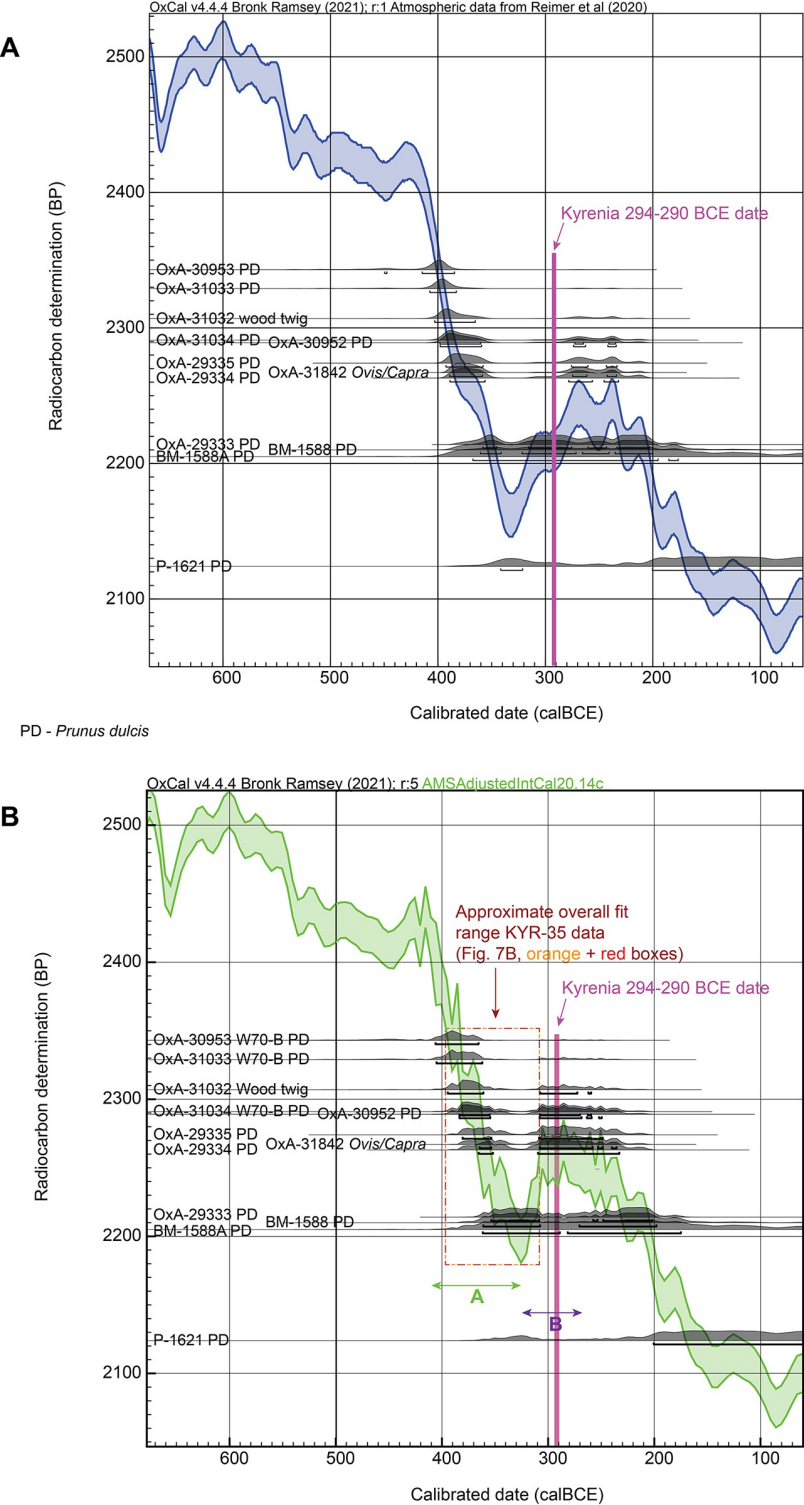
Comparison of the Kyrenia Ship ^14^C dates versus the IntCal20 calibration curve and versus the revised AMSAdjustedIntCal20 ^14^C calibration curve using the new data in S1 Table. A. Comparison of the ^14^C measurements on short-lived samples from the contents of the Kyrenia Ship against IntCal20 [10] (with the most likely 68.3% hpd ranges indicated). With IntCal20 most of the data are not compatible with the date of 294–290 BCE proposed for the ship’s wrecking in [1]. B. As A. but instead against the AMSAdjustedIntCal20 ^14^C dataset as in Figs 2B and 3B. The short-lived data now in all cases could include 294–290 BCE. Two possible fit areas are evident, labelled ‘A’ and ‘B’; ‘B’ offers a date range subsequent to the time period occupied by the KYR-8 tree-ring samples. Data from OxCal [43] version 4.4.4.

The previous pre-AMS ^14^C Pennsylvania (P) and British Museum (BM) dates extend down to 2124±60 ^14^C years BP. The wood twig (OxA-31032) and *Ovis/Capra* astragalus (OxA-31842) offer ages also within the almond range. The non-modelled calibrated calendar probabilities for each of these 12 dates, along with the most likely 68.3% ranges versus both IntCal20 and then the ‘AMSAdjustedIntCal20’ ^14^C dataset, are shown in Fig 8A and 8B.

Several observations are evident. First: looking at the dates versus IntCal20 (Fig 8A), there is surprisingly little dating probability consistent with the proposed 294–290 BCE wrecking date in [1]. Only 4 of the 12 dates include the 294–290 BCE date range within their most likely 68.3% calibrated calendar age probabilities (OxA-29333, OxA30953, BM-1588, BM-1588A). Five other dates could include later mid-3^rd^ century BCE ranges, but these seem too late based on the archaeological assessment of the Kyrenia Ship contents. Even at 95.4% probability, 4 of the 12 dates do not include 294–290 BCE. This might suggest a problem with the archaeological date, or, a problem with IntCal20 in this period—and the latter seems evident from the ‘AMSAdjustedIntCal20’ approximate curve and dataset shown in Figs 2B, 3B and 7B. Thus, rather than try to offer a solution with IntCal20, it seems that we should instead focus on the approximate new ^14^C calibration information in the ‘AMSAdjustedIntCal20’ ^14^C dataset and the correspondence of the dated samples with this (see Fig 8B). Second: if we look at the data versus the ‘AMSAdjustedIntCal20’ ^14^C dataset, there appear to be two plausible/possible fit options. An early possible date (also possible with IntCal20) in which all dated short-lived samples (excluding P-1621: see above) are situated around 400 BCE to around 330 BCE—thus more or less in the period of the KYR-35 samples (see Fig 7).

This possible early dating estimate, labelled as ‘A’ in Fig 8B, places the Kyrenia Ship’s construction TPQ (the KYR-35 sample) as effectively of the same or similar date as the date of its last voyage—meaning a ship that was essentially new when it sank. This would run against the long-held assessment that the ship had seen some period of service before it sank (see below). However, the main and fundamental problem with this ‘A’ date range is that the coins indicate a solid absolute minimum TPQ for the shipwreck of 334 BCE [6]. Since it is unlikely that the short-lived samples from the ship’s contents (or at least most of them) date more than a year or two before the wrecking, this leaves only the very end of the ‘A’ range as even plausible. Considering the evidence for a realistic last voyage TPQ at least little later (from coins and other material culture evidence [6–8]), around 325 BCE or even likely 310–306 BCE or from 294 BCE (see above), the ‘A’ range estimate becomes implausible.

The alternative is a possible more recent dating estimate labelled as ‘B’ in Fig 8B. The wiggle upwards in the AMSAdjustedIntCal20 ^14^C dataset at the end of the 4^th^ century BCE and then the plateau in the ^14^C curve in the early 3^rd^ century BCE offer another and conveniently short period of time that could accommodate most of the ^14^C dates on the short-lived samples (and just about all of them within measurement errors without being outliers). This ‘B’ range has the advantages of (i) being (shortly) after the date range indicated by the KYR-35 samples (which makes logical sense: the ship is built and then used and then we have the dates for the cargo of the last voyage), and (ii) it could offer dates for the last voyage that are compatible with the archaeological TPQ information and could include or be closer to the archaeological assessments ca. 310–290 BCE and especially 294–290 BCE.

### A dating model for the Kyrenia Ship

Given the considerations discussed above, we can now combine the information available into an overall dating model for the Kyrenia Ship (e.g. [15]). In essence we have a sequence in time:

*1. A Phase (1) that describes ship wood and construction or repairs to the ship*

This Phase comprises the following three elements:

1.1 KYR-8 (potentially containing some residual PEG) with the last extant tree-ring setting a TPQ for ship construction/repair and certainly its last voyage (LV). We allow for a generous (maximum plausible) Delta_R adjustment for this KYR-8 sample of 50±50 ^14^C years, since this appears to offer, and allow for, a best PEG-almost-free estimate.
1.2 KYR-35 (no PEG) with the last extant tree-ring of this sample setting a much closer TPQ for ship construction/repair and providing a TPQ for the LV.
A wooden treenail (no PEG) that sets perhaps a relatively close TPQ for construction/repair (and also a TPQ for the LV).

A Boundary after the above collective Phase 1 sets a TPQ for the ship construction or repair and so also for the LV. If we regard the contents of the last voyage of the ship as definitely later than this TPQ Boundary, then this Boundary acts as a TPQ for the date of the contents and last voyage (LV). Model 1 uses this assumption and this Boundary is labelled as “TPQ”. Since there is no evidence that the non-PEG-treated wood samples include outermost tree-rings, and every reason to suspect otherwise, and given evidence that the ship was in service for some period (with repairs: see below) such a TPQ model appears likely the most appropriate for this case.

Alternatively, the last voyage need not be long after ship construction or final repair (as dated by the information from the wood samples—but note there is no reason to assume the sample material available is near the original outermost tree-rings in this instance). In which case this Boundary, after the latest wood dating evidence, should in fact be regarded as effectively an estimate for the LV (which is not necessarily substantially later). But it must still be after the minimum coin TPQ of 334 BCE and plausibly after the likely TPQ ranges starting 325 BCE, or even the likely 310–306 BCE or 294 BCE TPQ [6]. Use, progressively, of each of these TPQs rules out what would otherwise be considerable ambiguity. Model 2 uses this assumption and the Boundary is labelled as “LV”. In Model 2 the LV Boundaries after the two Phases (1 and 2) are cross-referenced, since both act to inform on the date of the LV. As discussed below, the results with Model 2 indicate that the TPQ assumption of Model 1 must be more likely correct for this Kyrenia Ship case since Model 2 consistently identifies a date placement solution (the ‘A’ range shown in Fig 8B) that is too old—versus the plausible ‘B’ range.

*2. A Phase (2) with short/shorter-lived samples that likely in most cases relate to a period immediately before the Last Voyage (LV)*.

We may reasonably assume that most of the short-lived samples on board as cargo or crew provisions should date from immediately, or shortly, before the last voyage of the ship—although a few of the samples might be older, or even residual material. We can thus model this Phase (2) of dates between a Tau_Boundary and a Boundary in OxCal to create such an exponential distribution with the end of Phase 2 Boundary best describing the wrecking date immediately after the Phase [15]. This end Boundary can be labelled as ‘LV’ for last voyage. In view of the coins found on the ship we may set a conservative minimum TPQ for this LV Boundary of 334 BCE (from coins C3 and C5 in [3]). Models 1A and 2A use this exponential approach.

To check that the choice of an exponential modelling assumption is not biasing our analysis, we can also consider using a more neutral uniform Phase assumption: in which the short-lived data are placed within a Phase (2) bounded by two uniform Boundaries: Models 1B and 2B. Since a model of this form will inherently attempt to place all the data on the short-lived samples within a common and as short-as-possible modelled calendar probability range, we inherently expect some of the dates will exhibit poor OxCal individual Agreement (A) values—but it is worth noting that none of the dates on the short-lived samples is an outlier in either case (outlier probability above 5% probability) using the OxCal General Outlier model [45].

*3. The Boundary immediately following Phase (2), comprising the short/short-lived samples from cargo/crew supplies, best defines the LV, labelled “LV”.*

The additional key issue is how long in total is likely represented by the collection of short/shorter-lived samples from the wreck. Ancient wooden ships had relatively brief total use (or service) lifetimes, of the order of a few years to a few decades, and not much longer. Even evidence for repairs may only represent a total service period measured in years and not necessarily decades (or many decades). Thus, although there is evidence, from repairs and renovations, that the Kyrenia Ship was not new at the time of its wrecking [1–4, 61], it is implausible that there is a long period (e.g. >50+ years) between the latest wood elements of the ship (the last extant tree-rings of KYR-8 and especially KYR-35 and the wooden tree nail OxA-31701)—setting a TPQ for construction/repair of the ship—and the last voyage of the ship.

This assessment challenges a long-standing assumption about the Kyrenia Ship. Based on the very initial ^14^C dates from the Pennsylvania (P) laboratory which showed a reasonable (ca. 100 ^14^C years) age difference between a date on wood from the hull of the ship (unspecified regarding species and whether the sampling included outermost tree-rings) at 2222±43 ^14^C years BP (P-1622) and a date on the cargo of almonds at 2124±60 ^14^C years BP (P-1621) with an incorrect (typo or incorrect) δ^13^C value and no NaOH pretreatment, it has long been assumed that the Kyrenia Ship was quite old (with a long to very long service period) by the time it sank ([1] at p.29). The excavator, Katzev ([2] at p.14), for example, indicated maybe 80 years citing these two ^14^C dates. Apart from simple laboratory noise, this difference could merely be the result of dating tree-rings that were not the latest (outermost) from the original relevant timber.

However, based on what we know of comparable historical or ethnographic comparisons (e.g. [62] at p.17; [63] at p.142) and from shipwreck sites with dating information available [15, 64–66], an average, or even typical maximum, ship service period of 20–30 years might be estimated for antique wooden vessels, although many ships in fact had much shorter service periods. Thus, conservatively, we might limit the period represented in total by the collection of shorter/short-lived sample material from the shipwreck. The material dated in this case, mainly almonds from cargo, a twig, and an astragalus, may in reality represent just a couple to a handful of years and are very unlikely to represent a period longer than the typical ship service period. Thus we may apply a prior to the time constant (Tau) for the exponential distribution in a revised version of the models. A uniform probability period of 0–60 years (double the expected ‘average’) is both reasonable and very conservative and is used in Models 1C and 2C. We regard these two models are likely offering our best age estimates—and, since we find Model 2 to be unrealistic in view of the ship and ^14^C data available, this leaves Model 1C as our best dating estimate.

**Fig 9:**
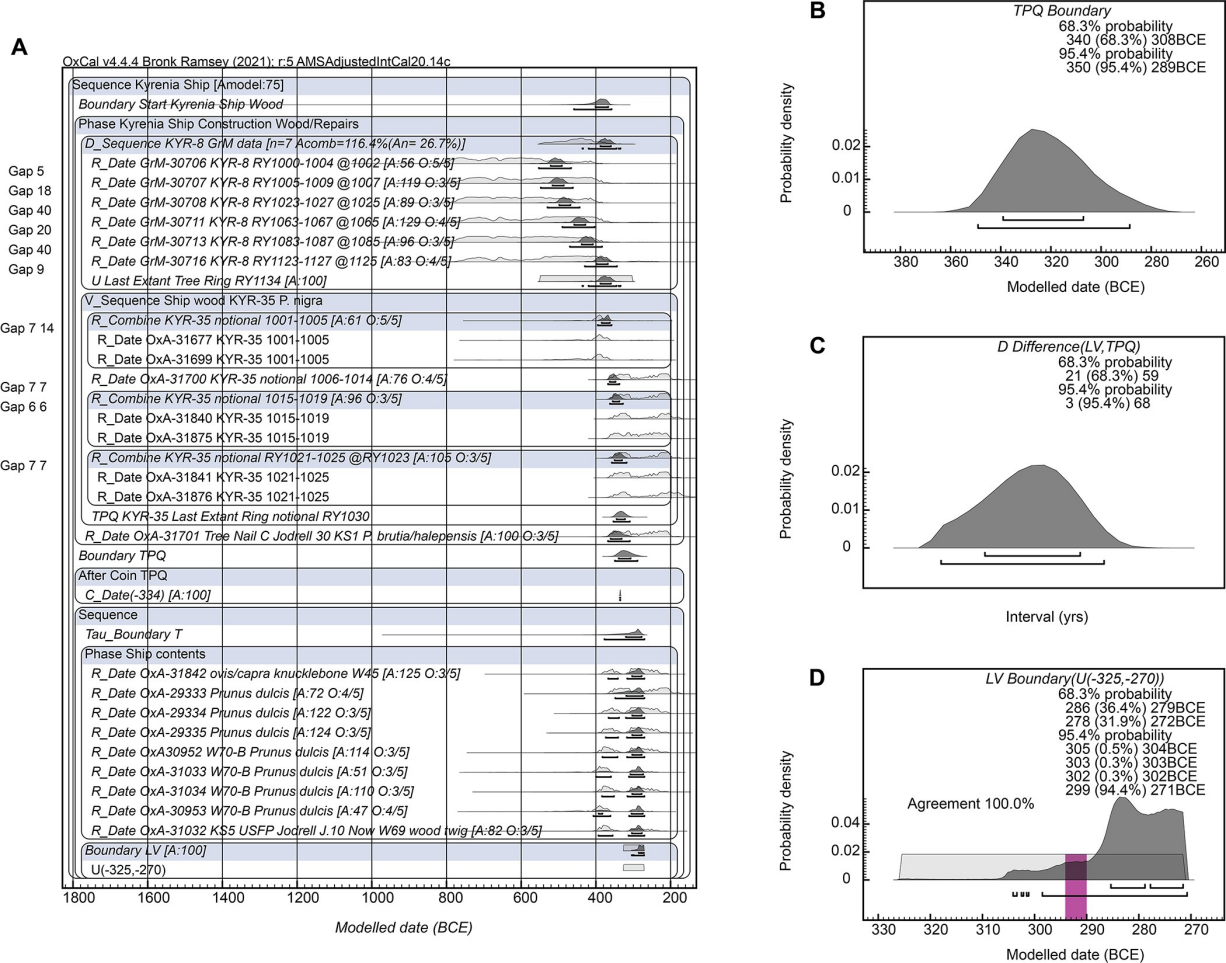
Model 1C using the revised AMSAdjustedIntCal20 ^14^C dataset and selected results. A. whole model. The OxCal keywords, numerical values, and outlining indicate the structure of the model exactly. The light shaded distributions are the non-modelled calibrated calendar probabilities; the smaller dark histograms indicate the modelled probability with the lines under these indicating the 68.3% and 95.4% hpd ranges. B. Detail of the TPQ Boundary. C. Detail of the LV Boundary. D. Detail of the Difference query (time interval between the TPQ and the LV). Data from OxCal [43, 45] version 4.4.4.

**Fig 10:**
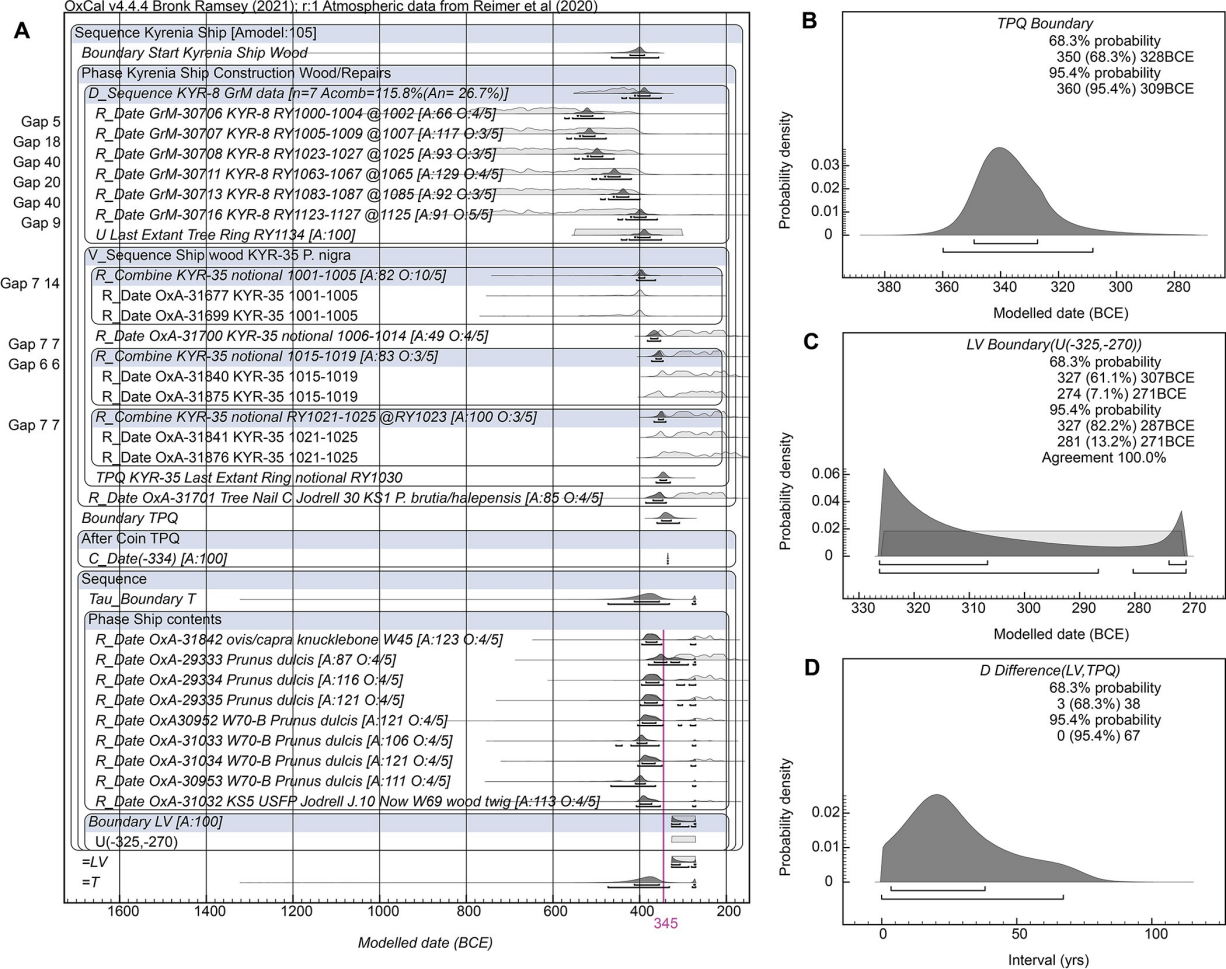
Model 1C using the previous IntCal20 dataset [10] and selected results—compare with Fig 9. A. Whole model. The OxCal keywords, numerical values, and outlining indicate the structure of the model exactly. The light shaded distributions are the non-modelled calibrated calendar probabilities; the smaller dark histograms indicate the modelled probability with the lines under these indicating the 68.3% and 95.4% hpd ranges. Note, with IntCal20, the main (>90% probability range) of the overall modelled 95.4% hpd ranges of 8 of 9 of the short-lived samples from the ship contents entirely lie no later than 345 BCE. B. Detail of the TPQ Boundary. C. Detail of the LV Boundary. D. Detail of the Difference query (time interval between the TPQ and the LV). Data from OxCal [43, 45] version 4.4.4.

We thus consider 6 models (1A, 1B, 1C, 2A, 2B, 2C). Based on the prior information available, in each model the date of the ship contents is placed after a (minimum) TPQ of 334 BCE and the possible date range for the LV Boundary is constrained (uniform probability) as lying between 325 BCE (the conservative TPQ from coin and ceramic evidence) and 270 BCE (a very conservative TAQ from all evidence from the ship: see above). In Models 1A-C a Difference query determines the probability for the length of time in calendar years between the TPQ Boundary and the LV Boundary. (We note that the TPQ Boundary is an earliest possible ship construction or repair date—since we know that there are an unknown number of tree-rings missing between the last extant rings and the original outermost ring when the trees were felled—hence the likely real construction or repair date is later than this TPQ). Model 1C is shown as the example in Fig 9 using the new AMSAdjustedIntCal20 ^14^C dataset, and then in Fig 10 using IntCal20. All models are shown in S2–S9 Figs. Comparison with the ‘minimal-adjustment’ calibration curve, ‘AMSAdjustedIntCal20_GrMbased’, as shown in Fig 4B, is illustrated in S9 Fig (compare with Fig 9). We also show a run of Model 1C using a 1-year resolution version of the AMSAdjustedIntCal20 ^14^C dataset in S8 Fig. This 1-year resolution version produces much more ‘noisy’ distributions, but the results for the modelled TPQ and LV Boundaries are very similar (see S8 Fig compared with Fig 9 and Table 3). The OxCal Amodel/Aoverall values, the date ranges for the TPQ Boundary (where appropriate) and the LV Boundary, and the results of the Difference query (for Model 1) for the various model versions, both with the new AMSAdjustedIntCal20 ^14^C dataset and with IntCal20, are listed in Table 3. The OxCal runfiles are listed in S2 File. As outlined above, we consider the runs against the AMSAdjustedIntCal20 ^14^C dataset to offer the plausible likely approximate calendar age ranges.

**Table 3:** Summary of different models and date ranges for the Last Voyage (LV) Boundary. The table shows the 68.3% and 95.4% hpd calendar date BCE (Cal BCE) ranges for the TPQ Boundary (Models 1A to 1C only) and LV Boundary from each of Models 1A-1C and 2A-2C, and the Difference query for the time period between the TPQ and the LV for Models 1A to 1C, along with OxCal Amodel (Am) and Aoverall (Ao) values across the 6 models, and comparing the values achieved with the new AMSAdjustedIntCal20 ^14^C dataset (with curve resolution set at 5 years) versus those from IntCal20 (with curve resolution set a 1 year) in grey shading. The Am/Ao values for Model 1B are unsatisfactory (<60), indicating that this model’s assumptions are not compatible with the ^14^C data and the ^14^C calibration curve. For Model 1C, as an example, we also list the results if the AMSAdjustedIntCal20 ^14^C dataset is used but with 1 year curve resolution (**bold** text). While the 1-year resolution model results overall are noisier (see S8 Fig), the findings for the TPQ and LV Boundaries are very similar to the 5-year resolution models. For Model 1C we list also the results employing the GrM and European oak adjusted overall dataset (see Fig 4B), ‘AMSAdjustedIntCal20_GrMbased’, in underlined text (see S9 Fig). Finally, for Model 1C, we list the results if the two apparently (subjectively) somewhat older ^14^C ages, for OxA-30953 and OxA-31033 (both have individual OxCal Agreement values <60, respectively 47 and 51: Fig 9), are excluded from the analysis (*italicized* text). This makes little difference. Since neither date is an outlier within the model (outlier probabilities respectively 4% and 3%, both <5%: Fig 9) we leave them in.

	Am/Ao Black AMSAdjusted- IntCal20 GreyIntCal20	68.3% hpd Date Range BCE AMSAdjusted- IntCal20 GreyIntCal20	95.4% hpd Date Range BCE Black AMSAdjusted- IntCal20 GreyIntCal20
**Model 1A TPQ**	75/63 106/95	340–308 349‐328	350–289 360‐309
**Model 1A LV**		286–272 (37.6%) 327‐305(64.9%),274‐272(3.4%)	305–303 (1.1%), 299–271 (94.4%) 327‐288(84.2%),281‐271(11.2%)
**Model 1A Difference** **LV-TPQ**		*21–59 years* 4‐37years	*3–68 years* 0‐65years
**Model 1B TPQ**	44/39 14/13	340–312 351‐322	351–297 358‐287
**Model 1B LV**		286–278 (41.3%), 277–272 (27.0%) 273‐271	305–302 (2.4%), 297–271 (93.0%) 276‐271
**Model 1B Difference LV-TPQ**		*27–60 years* 49‐79years	*11 to 71 years* 15‐86years
**Model 1C TPQ**	77/68 *73/67 ǂ102/90 *#89/81* 105/93	346–312 *345–313 ǂ345–318 *#340–308* 350‐328	356–291 *355–291 ǂ353–292 *#350–289* 360‐309
**Model 1C LV**		286–279 (40.2%), 278–272 (28.1%) *286–278 (44.9%), 276–272 (23.3%) ǂ326–324 (3.0%),293–271 (65.3%) *#287–272* 327‐307(61.1%),269‐262(7.1%)	305–303 (1.6%), 298–271 (93.9%) *306–301 (6.0%), 296–271 (89.4%) ǂ327–314 (14.5%), 306–271 (81.0%) *#302–302 (0*.*7%)*, *301–271 (94*.*7%)* 327‐287(82.2%),281‐271(13.2%)
**Model 1C Difference LV-TPQ**		*28–64 years* *26–64 years ǂ14–59 years *#20–58 years* 3‐38years	*5–75 years* *4–73 years ǂ0–69 years *#3–68 years* 0‐67years
**Model 2A LV**	82/75 116/110	305–303 (5.0%), 296–293 (5.3%), 289–272 (58.0%) 327‐317	327–320 (5.5%), 307–271 (89.9%) 327‐391(95.3%),272‐272(0.1%)
**Model 2B LV**	95/89 128/121	327–312 (39.4%), 306–298 (13.2%), 296–296 (0.5%), 287–279 (15.2%) 327‐316	327–309 (44.7%), 308–291 (25.8%), 290–274 (24.9%) 327‐292
**Model 2C LV**	83/76 115/109	305–302 (5.6%), 296–294 (4.7%), 289–272 (58.0%) 327‐317	327–320 (5.0%), 307–271 (90.5%) 327‐293(94.6%),274‐272(0.8%)

### Inclusivity in global research

Additional information regarding the ethical, cultural, and scientific considerations specific to inclusivity in global research is included in the Supporting Information (S3 File, Checklist).

## Results

The results from the various models are compared in Figs 9 and 10 and S2–S9 Figs. S2–S4 and S8 Figs use the new revised AMSAdjustedIntCal20 ^14^C dataset, S5–S7 Figs use IntCal20, and S9 Fig employs the more minimal offset AMSAdjustedIntCal20_GrMbased curve. The results for the main elements of each model, or an example using the preferred Model 1C, are listed in Table 3. The results with more details from Model 1C using the new AMSAdjustedIntCal20 ^14^C dataset are shown in Fig 9 and can be compared to those from the same model using IntCal20 in Fig 10.

It is notable looking at S5–S7 Figs and Fig 10 that 5 of the 6 model runs using IntCal20 place the modeled probability from the short-lived samples from the Kyrenia Ship in the 4^th^ century BCE and place the majority or almost all of the Last Voyage (LV) Boundary strongly in the 4^th^ century BCE (thus range ‘A’ in Fig 8B). For example, looking at Fig 10 showing Model 1C with IntCal20, the vast majority of the modelled 95.4% hpd probability from the short-lived samples from the Kyrenia Ship is placed no later than 345 BCE (i.e. before the magenta line in Fig 10A), whereas the archaeological TPQ data indicate a date definitely after 334 BCE and likely after 325 BCE and probably after 310–306 BCE or 294 BCE [1, 6–8]. The sole exception, Model 1B with IntCal20 (S6 Fig), identifies a LV Boundary range in the 270s BC, but exhibits very poor model agreement (Amodel/Aoverall of 14/13 less than the satisfactory level of ca. 60). The fact that the Last Voyage (LV) Boundary dates are even later 4^th^ century BCE (versus earlier in the 4^th^ century BC) in the 5 of 6 cases for the IntCal20 runs is clearly solely due to the imposition within the model of the archaeological TPQs of 334 BCE and 325 BCE—the calibration data from IntCal20 in fact point earlier in the 4^th^ century BC. The Model 1C case shown in Fig 10 illustrates this observation. These findings suggest that IntCal20 does not offer a correct calendar dating for the Kyrenia Ship.

In contrast, although only approximately modelled (versus the more elaborate IntCal curve modelling process in [51]), the model runs reported with the new AMSAdjustedIntCal20 ^14^C dataset offer calendar placements for the short-lived samples and the LV Boundary date range clearly or decisively compatible with the archaeological TPQ information in 5 of 6 cases (Fig 9, S2–S4, S8 Figs, Table 3)—and thus also consistent with a likely TPQ of 310–306 BCE or 294 BCE and in the area of range ‘B’ in Fig 8B. The sole partial exception is Model 2B where the placement is more ambiguous (although, even then, the majority, 50.7% of the probability, for the LV is 308–274 BCE versus 44.7% for 327–309 BCE when examining the 95.4% hpd ranges: Table 3).

**Fig 11:**
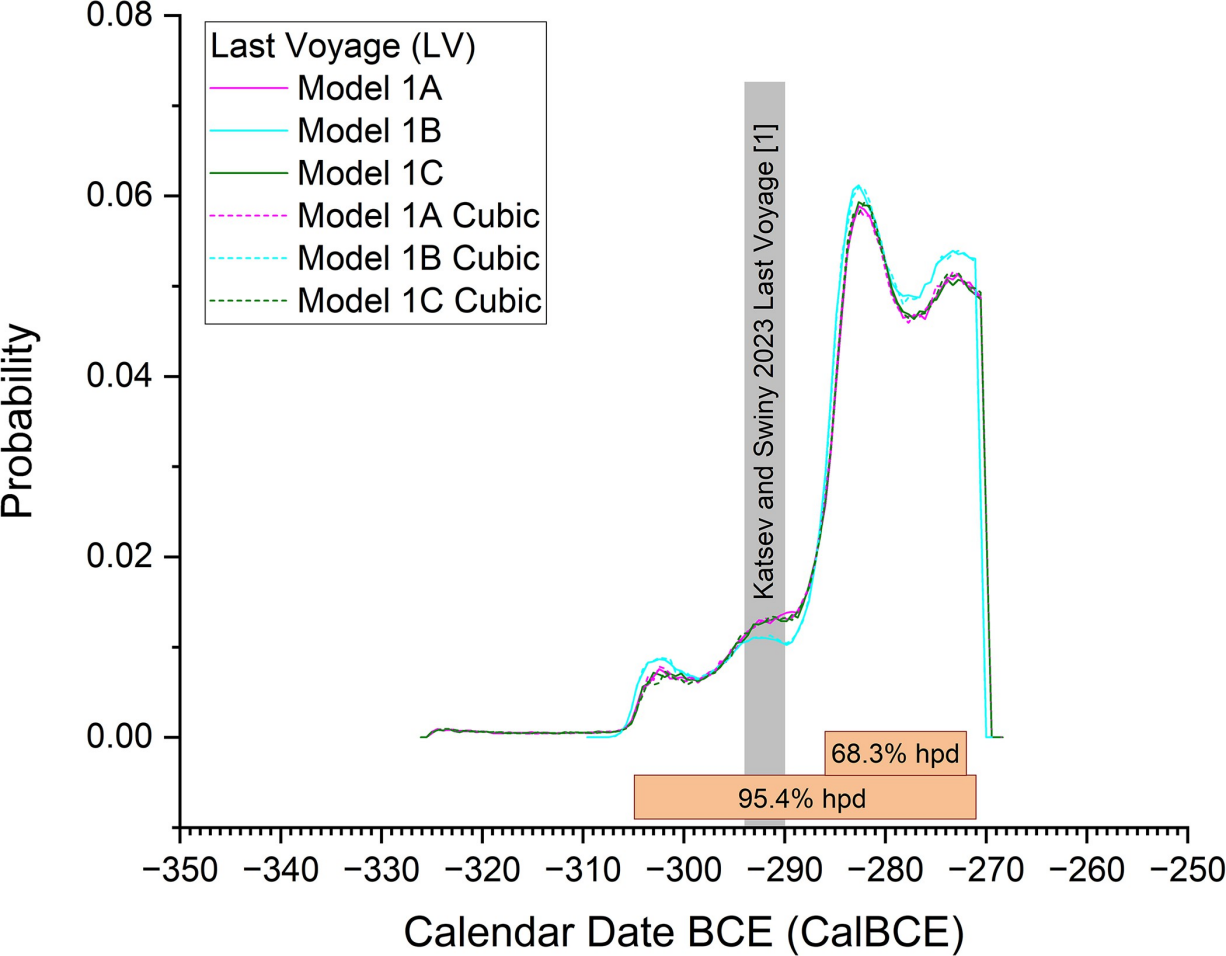
The highest posterior density (hpd) distributions for the Last Voyage (LV) Boundary from Models 1A, 1B and 1C with the AMSAdjustedIntCal20 ^14^C dataset with curve resolution set at 5 years and with linear interpolation and also the same but using cubic interpolation all from OxCal [43] version 4.4.4. The indicated 68.3% and 95.4% hpd ranges, 286–272 BCE (68.3%) and 305–271 BCE (95.4%) are the upper and lower limits from each version of Models 1A, 1B and 1C. The only variation is that three models begin the 95.4% range at 305 BCE and three at 304 BCE across our runs.

It is further evident, examining each of Models 1A-C versus Models 2A-C all using the AMSAdjustedIntCal20 ^14^C dataset, that the assumption that the Phase with the short-lived samples from the ship contents is later than the TPQ from the last extant ship timber samples (Model 1) yields results more consistent both with the available Kyrenia Ship dataset and with the archaeological TPQ information. Hence we prefer the results from Model 1. Here, across all models with the AMSAdjustedIntCal20 ^14^C dataset, the LV Boundary is placed between 306/305/302-271 BCE at 95.4% hpd and the most likely 68.3% hpd range is 287/286-272 BCE (Fig 9, S2–S4 Figs, Table 3). We note that if the AMSAdjustedIntCal20 ^14^C dataset is modelled not by simple linear interpolation, but instead by a cubic function in OxCal, then the LV boundary dates are almost identical (see Fig 11). The most likely date ranges for the LV from the more minimally adjusted AMSAdjustedIntCal20_GrMbased ^14^C dataset are also similar for Model 1C: 293–271 BCE as the main likely sub-range of the 68.3% hpd range, and 306–271 BCE as the main likely sub-range of the 95.4% hpd range (see Table 3).

## Discussion

### Kyrenia Ship

Archaeological information provides calendar TPQ constraints for the dating of the Kyrenia Ship [1, 6–8]. The currently available AMS ^14^C dates on the materials from the Kyrenia Ship and their modelling do not offer a satisfactory calendar date range for the ship using the IntCal20 ^14^C calibration curve [10]. The issue appears to be that IntCal20 in the relevant period, between ca. 350–250 BC, is based on legacy non-AMS ^14^C data and these legacy data appear to exhibit, as noted in other portions of the calibration record, small differences from modern AMS ^14^C measurements. We have therefore measured AMS ^14^C data on known-age tree-ring samples across this calendar period and the resultant calibration record (our AMSAdjustedIntCal20 ^14^C dataset: Figs 2B and 3B) appears, then, to offer a satisfactory correspondence between the archaeological dating information relevant to the Kyrenia Ship and the AMS ^14^C dates on materials from the ship.

The available ^14^C data point to a most likely last voyage (LV) date range that is slightly later (more recent) than the current archaeological assessment of 310–290 BCE and especially 294–290 BCE [1]. The ^14^C modelling includes the years 304–290 BCE within the less likely region of the overall 95.4% hpd range (and so this date range is possible), but points to a most likely 68.3% LV range of 286–272 BCE (although the minimally adjusted AMSAdjustedIntCal20_GrMbased ^14^C dataset extends the most likely sub-range at 68.3% hpd to 293–271 BCE, see Table 3, which could allow the current 294–290 BCE assessment). Within the overall 68.3% hpd range using the AMSAdjustedIntCal20 ^14^C dataset—which because it includes the sequoia data may likely offer a better approximate representation of the relevant atmospheric ^14^C record for the Mediterranean samples (see above)—the sub-range 286–279 BCE is slightly more likely for Models 1A and 1C (Table 3, Fig 9). If an unsmoothed 1-year resolution version of the AMSAdjustedIntCal20 ^14^C dataset is employed with Model 1C, then this more clearly favors the earlier range 286–278 BCE (44.9% hpd) versus 276–272 BCE (23.3% hpd): S8 Fig.

The available archaeological dating information indicates a TPQ perhaps (latest interpretation of coin C7) as late as 294–290 BC, but does not restrict the date being a little after this TPQ information. Indeed, by definition, a coin find in an archaeological context, even when it can be dated, sets only a TPQ (not a date), and there can be numerous complications involved [67]. A last voyage (wrecking) date of 286–272 BCE (and especially in the earlier years of this range, e.g. 286–278 BCE) would not be incompatible with any of the available archaeological/epigraphic information from the ship.

Achieving a date range that corresponds with the available archaeological TPQ information also supports use of the assumption in Model 1 that the TPQ available from the last extant wood elements of the ship is before the date of the short-lived samples from ship contexts from the last voyage of the ship. This is inherently plausible in this case, since there is no evidence any of the wood elements dated contain outermost tree-rings that might date exactly either the construction or a repair to the ship. In each case, there are an unknown number of tree-rings absent between preserved sample material and the original outermost tree-ring from the tree stem.

Despite the complications introduced by the application of PEG, and after best efforts to remove this and to allow for plausible remaining contaminants, the KYR-8 timber and the pattern of the time-series of ^14^C dates from this timber demonstrate that the last extant tree-ring belongs no later than the very start of the steep slope (change) in atmospheric ^14^C levels shortly before 400 BCE (Figs 3 and 5). Since the wood was a potentially long-lived species (*Pinus nigra*), even a small to modest amount of missing original outer wood could easily represent many decades of missing tree-rings. Thus this sample suggests a construction or repair date somewhere in the 4^th^ century BCE.

The KYR-35 wood sample was less than straightforward, but the ordered pattern of the ^14^C data (two groups replicated) ties this sample to the earlier to mid-4^th^ century BCE. The ^14^C date on the tree-nail (OxA-31701) could then date either in a similar earlier to mid-4^th^ century BCE range or later.

This leaves the construction or repair of the Kyrenia Ship TPQ as ca. 356 to 289 BCE (95.4% hpd) and most likely ca. 346/340 to 312/308 BCE (68.3% hpd) from Models 1A-C using the AMSAdjustedIntCal20 ^14^C dataset (Table 3). This date range is a TPQ. Hence a date for ship’s construction or repair in the later 4^th^ and even late 4^th^ century BCE is probable. The intervals listed in Table 3 for Models 1A-C between the TPQ and the LV Boundaries are thus maximum possible estimates for the ship’s service period. In reality, the actual construction/repair date is after the TPQ and so the service period was shorter.

The exact calendar age ranges we have provided from our interim and approximate AMSAdjustedIntCal20 ^14^C dataset will be revised when the next version of IntCal (including these data) is available. However, the fundamental finding that IntCal20 needs revision in this time period, and that we have provided modern AMS ^14^C data on known-age wood which confirm and approximately quantify this revision will remain valid. A case like this highlights the importance and usefulness of the on-going shift to a new ^14^C calibration timescale that is informed with modern high-resolution AMS ^14^C data on annual resolution known-age materials. This both removes issues of small apparent offsets between (at least some) legacy pre-AMS ^14^C data and modern AMS ^14^C measurements and allows issues of high-resolution details in calibration to be identified and investigated. These types of issues are especially key in cases like the Kyrenia Ship where archaeological dating requires accuracy and precision at the level of a couple of decades or less.

### Applying the new calibration curve data to the Mazotos ship

The revised ^14^C calibration data reported in this paper and used for the dating of the Kyrenia Ship are relevant also to other Classical-Hellenistic era ships, including the recently reported dating of the Mazotos ship found off the southern coast of Cyprus [15]. A combination of tree-ring-sequenced ^14^C wiggle-matching and ^14^C dates on short-lived materials from the Mazotos ship using IntCal20 placed the last extant tree-ring (a TPQ for construction of the ship) at 442–399 BCE at 95.4% hpd (422–404 BCE at 68.3% hpd) and the date for the ship’s Last Voyage (LV) 393–374 BCE at 95.4% hpd (390–382 BCE at 68.3% hpd).

**Fig 12:**
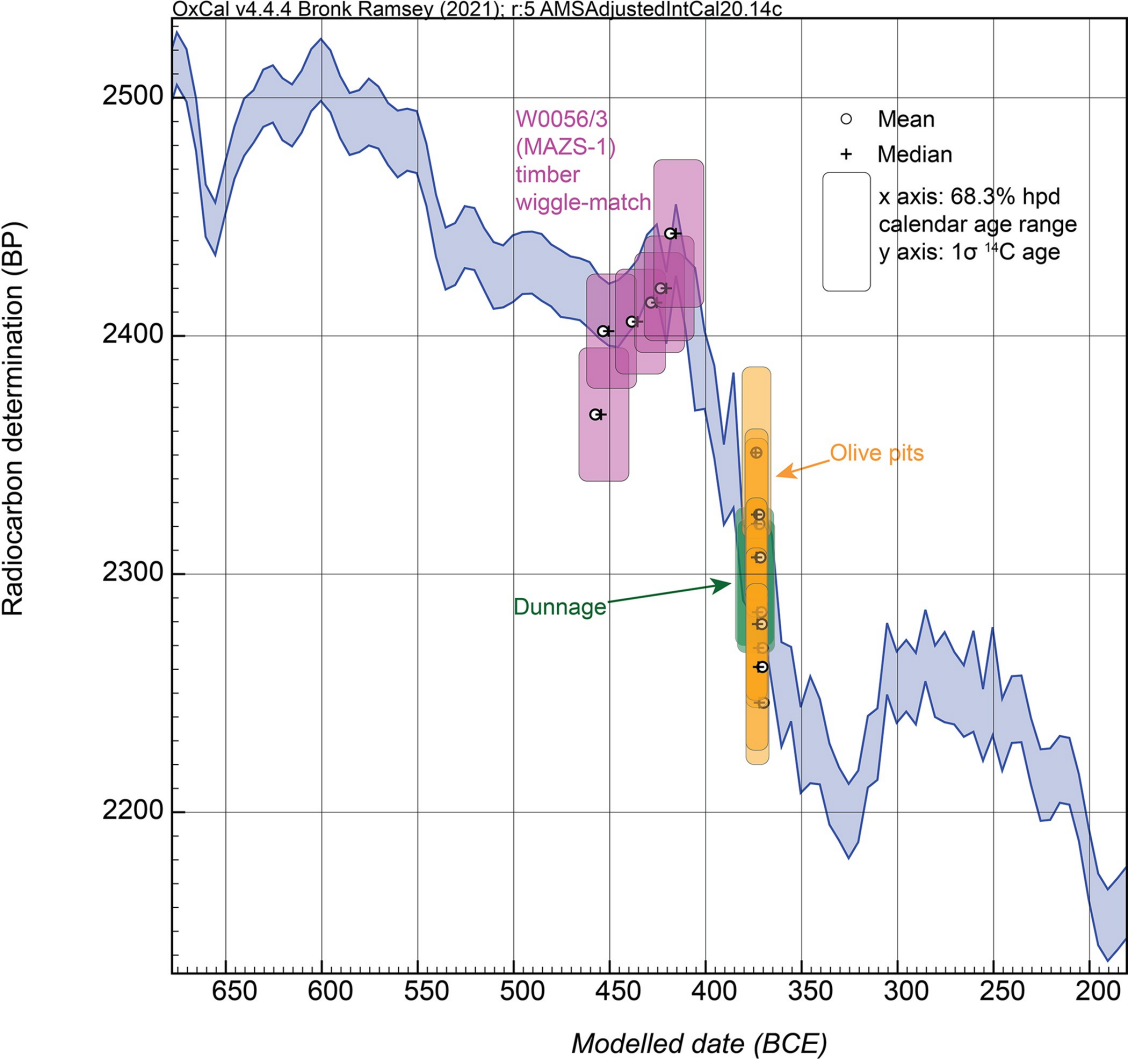
Dating the Mazotos ship and contents. We show a revised version of results from the model in [15] at Fig 5 using now the AMSAdjustedIntCal20 ^14^C dataset (as in Figs 2B and 3B) showing the placements with these data of the timber (MAZ-1) wiggle-match and dunnage and olive pit samples from the dating model for the Mazotos ship (see [15]).

Re-running the same Mazotos dating model with the approximate revised calibration curve reported in this paper (Figs 2B and 3B) leads to dates of 426–400 BCE (95.4% hpd) and 417–414 BCE (68.3% hpd) for the last extant tree-ring/construction TPQ and 383–355 BCE (95.4% hpd) and 377–364 BCE (68.3% hpd) for the ship’s Last Voyage. The new calibration data point to a slightly later calendar date range for the Mazotos ship, but, nonetheless, place the timber in the period before ca. 400 BCE. It is indeed noticeable that the revised calibration data slightly better fit, especially the last part, the tree-ring wiggle-match from the Mazotos timber. This revised calibration places the short-lived samples defining the ship’s Last Voyage on the steep slope in the ^14^C calibration curve and no later than 367/358 BCE (68.3%/95.4% hpd): see Fig 12. This revised date range at 95.4% hpd is 5–58 years earlier than the original date estimate (350–325 BCE) based on the ceramic material and continues to suggest that this ceramic-based date needs a small but critical revision.

### Importance of appropriate calibration dataset to historical-level analyses, and post-PEG potentials

Recent work has highlighted small but important differences in various periods between modern AMS ^14^C measurements and some legacy routine (non-AMS) ^14^C measurements (e.g. [11–13, 47–50, 68]). Especially when dealing with historical periods, even such usually small differences (often e.g. 10–15 ^14^C years BP) become important when using ^14^C to inform on historical dating or when comparing independent historically-derived dates against those achieved via ^14^C, as in this paper. Such accuracy and appropriate (comparable technology) comparisons also become key when considering the implications of differences in growing seasons, and therefore ^14^C ages, between regions for some plants and growth contexts [13, 57, 58, 69]. The modified ^14^C calibration time series presented and used in this paper is thus important and relevant for high-precision dating using ^14^C broadly in the late Classical and Hellenistic periods for all archaeological contexts—and not only maritime cases as here.

The presence of PEG has often been seen as defining the end of any potential for achieving calendar age estimates via ^14^C for wood from historical and ancient ships. The effective removal of PEG, especially when considerable time has elapsed after treatment, has long been considered impossible. While inevitably a challenge, we have shown in line with other work [35] that it is, however, possible to largely remove PEG from samples to achieve relevant information—although consideration, even then, of potential very small remnant traces can remain, as appears to be the case for the Kyrenia KYR-8 samples.

## Supporting information

S1 Table ^14^C measurements on known-age (dendrochronologically dated) single-year tree-ring samples from the period 433–250 BC run at the Groningen (GrM) and KECK Carbon Cycle AMS Facility (UCIAMS). (DOCX)

S1 File Details on the tree-ring samples dated at UCIAMS and first reported in S1 Table (for details on the tree-ring samples dated at GrM, see [14]). (DOCX)

S2 File OxCal runfiles for the models and ‘AMSAdjustedIntCal20’ and ‘AMSAdjustedIntCal20_GrMbased’ calibration data employed 2382–2199 CalBP. (DOCX)

S3 File Checklist–Additional information regarding the ethical, cultural, and scientific considerations specific to inclusivity in global research. (DOCX)

S1 Fig Crossdating results using COFECHA [18] comparing the measurements on the *Sequoiadendron giganteum* D22SEC sample (RY1-1382) versus those for known-age *Sequoiadendron giganteum* trees offering substantive overlaps from the Mountain Home combined chronology (ITRDB CA717: https://www.ncei.noaa.gov/access/paleo-search/study/32186). The last year of overlap is indicated and the *t* value is reported. The D22SEC series is placed (removing a year 0 from the Mountain Home combined chronology) from -558 BCE to 824 CE.
(JPG)

S2 Fig Results from Model 1A compared with Model 2A, both employing the AMSAdjusted IntCal20 ^14^C dataset with curve resolution set at 5 years. Both these models use an exponential probability Phase for the short-lived materials from the ship (compared with the uniform probability Phase assumption in the B models). The upper and lower lines under the distributions show respectively the 68.3% and 95.4% hpd calendar age ranges. Data from OxCal [43, 45] version 4.4.4.
(JPG)

S3 Fig Results from Model 1B compared with Model 2B, both employing the AMSAdjusted IntCal20 ^14^C dataset with curve resolution set at 5 years. Both these models use a uniform probability Phase for the short-lived materials from the ship (compared with the exponential Phase assumption in the A and C models). The upper and lower lines under the distributions show respectively the 68.3% and 95.4% hpd calendar age ranges. Data from OxCal [43, 45] version 4.4.4.
(JPG)

S4 Fig Results from Model 1C compared with Model 2C, both employing the AMSAdjusted IntCal20 ^14^C dataset with curve resolution set at 5 years. Both these models use an exponential probability Phase for the short-lived materials from the ship like Models 1A and 2A, but now with a time constant, Tau, defining the exponential distribution which has a uniform prior assigned between 0 and 60 calendar years. The upper and lower lines under the distributions show respectively the 68.3% and 95.4% hpd calendar age ranges. Data from OxCal [43, 45] version 4.4.4.
(JPG)

S5 Fig Results from Model 1A compared with Model 2A, both employing the IntCal20 dataset with curve resolution set at 1 year. Both these models use an exponential probability Phase for the short-lived materials from the ship (compared with the uniform probability Phase assumption in the B models). The upper and lower lines under the distributions show respectively the 68.3% and 95.4% hpd calendar age ranges. Data from OxCal [43, 45] version 4.4.4.
(JPG)

S6 Fig Results from Model 1B compared with Model 2B, both employing the IntCal20 dataset with curve resolution set at 1 year. Both these models use a uniform probability Phase for the short-lived materials from the ship (compared with the exponential Phase assumption in the A and C models). The upper and lower lines under the distributions show respectively the 68.3% and 95.4% hpd calendar age ranges. Data from OxCal [43, 45] version 4.4.4.
(JPG)

S7 Fig Results from Model 1C compared with Model 2C, both employing the IntCal20 dataset with curve resolution set at 1 year. Both these models use an exponential probability Phase for the short-lived materials from the ship like Models 1A and 2A, but now with a time constant, Tau, defining the exponential distribution which has a uniform prior assigned between 0 and 60 calendar years. The upper and lower lines under the distributions show respectively the 68.3% and 95.4% hpd calendar age ranges. Data from OxCal [43, 45] version 4.4.4.
(JPG)

S8 Fig Model 1C using the AMSAdjusted IntCal20 ^14^C dataset with curve resolution set at 1 year (versus 5 years in Fig 9, S7 Fig) and selected results (see also Table 3). A. whole model. The OxCal keywords, numerical values, and outlining indicate the structure of the model exactly. The light shaded distributions are the non-modelled calibrated calendar probabilities; the smaller dark histograms indicate the modelled probability with the lines under these indicating the 68.3% and 95.4% hpd calendar age ranges. B. Detail of the TPQ Boundary. C. Detail of the LV Boundary. D. Detail of the Difference query (time interval between the TPQ and the LV). Data from OxCal [43, 45] version 4.4.4.
(JPG)

S9 Fig Model 1C using the AMSAdjusted IntCal20_GrMbased ^14^C dataset with curve resolution set at 5 years and selected results, compare with Fig 9 (see also Table 3). A. whole model. The OxCal keywords, numerical values, and outlining indicate the structure of the model exactly. The light shaded distributions are the non-modelled calibrated calendar probabilities; the smaller dark histograms indicate the modelled probability with the lines under these indicating the 68.3% and 95.4% hpd calendar age ranges. B. Detail of the TPQ Boundary. C. Detail of the LV Boundary. D. Detail of the Difference query (time interval between the TPQ and the LV). Data from OxCal [43, 45] version 4.4.4.
(JPG)
