# Supplementary material for: A revised radiocarbon calibration curve 350–250 BCE impacts high-precision dating of the Kyrenia Ship
Source: PLoS One. 2024 Jun 26;19(6):e0302645. doi: 10.1371/journal.pone.0302645 (PMC11207157; doi:10.1371/journal.pone.0302645)
Supplement: S1 Table — (DOCX) [file pone.0302645.s001.docx]

**Table S1**. Radiocarbon measurements on known-age (dendrochronologically dated) single-year tree-ring samples from the period 433-250 BC run at the Groningen (GrM) and KECK Carbon Cycle AMS Facility (UCIAMS). The primary publication of GrM samples with further information is in [14]. The samples marked in yellow highlight are both on the same sample and weighted average values are also reported. For pretreatment and methods for the GrM samples, see [14,36]. For pretreatment and methods for the UCIAMS dates, see [50]. For the GrM measurements separate IRMS δ^13^C values are listed. For the UCIAMS measurements the results have been corrected for isotopic fractionation with δ^13^C values measured on the prepared graphite using the AMS. Since these can differ from the original δ^13^C of the sample material these values are not listed for UCAIMS results. Separate IRMS values are not available. For details on the tree-ring samples, see File S1.

| **GrM #** | **Tree** | **Tree-Ring  Date BC** | **cal BP** | **δ^13^C ‰** | **^14^C age BP** | **±** | **W. av.** | **±** |
| --- | --- | --- | --- | --- | --- | --- | --- | --- |
| 19961 | *Quercus* sp. | 426 | 2375 | -23.79 | 2437 | 21 |  |  |
| 19963 | *Quercus* sp. | 422 | 2371 | -23.33 | 2419 | 21 |  |  |
| 19966 | *Quercus* sp. | 418 | 2367 | -24.76 | 2448 | 20 |  |  |
| 20099 | *Quercus* sp. | 414 | 2363 | -24.78 | 2453 | 21 |  |  |
| 19968 | *Quercus* sp. | 413 | 2362 | -26.33 | 2428 | 20 |  |  |
| 19967 | *Quercus* sp. | 413 | 2362 | -26.36 | 2448 | 20 | 2438 | 15 |
| 19969 | *Quercus* sp. | 412 | 2361 | -25.95 | 2414 | 20 |  |  |
| 19970 | *Quercus* sp. | 411 | 2360 | -25.45 | 2425 | 21 |  |  |
| 19971 | *Quercus* sp. | 410 | 2359 | -25.37 | 2414 | 20 |  |  |
| 19972 | *Quercus* sp. | 409 | 2358 | -25.40 | 2413 | 20 |  |  |
| 19974 | *Quercus* sp. | 408 | 2357 | - | 2392 | 20 |  |  |
| 19973 | *Quercus* sp. | 408 | 2357 | -24.74 | 2385 | 20 | 2389 | 15 |
| 19975 | *Quercus* sp. | 407 | 2356 | -24.62 | 2436 | 23 |  |  |
| 20100 | *Quercus* sp. | 406 | 2355 | -24.27 | 2386 | 21 |  |  |
| 20101 | *Quercus* sp. | 405 | 2354 | -24.57 | 2385 | 20 |  |  |
| 20102 | *Quercus* sp. | 404 | 2353 | -24.43 | 2365 | 20 |  |  |
| 20104 | *Quercus* sp. | 403 | 2352 | -24.27 | 2393 | 20 |  |  |
| 20106 | *Quercus* sp. | 402 | 2351 | -24.57 | 2381 | 20 |  |  |
| 20105 | *Quercus* sp. | 402 | 2351 | -24.47 | 2368 | 21 | 2375 | 15 |
| 20107 | *Quercus* sp. | 401 | 2350 | -24.26 | 2378 | 21 |  |  |
| 20109 | *Quercus* sp. | 400 | 2349 | -24.46 | 2390 | 20 |  |  |
| 20112 | *Quercus* sp. | 399 | 2348 | -24.51 | 2361 | 21 |  |  |
| 20113 | *Quercus* sp. | 398 | 2347 | -23.54 | 2404 | 20 |  |  |
| 20115 | *Quercus* sp. | 397 | 2346 | -23.38 | 2352 | 21 |  |  |
| 20114 | *Quercus* sp. | 397 | 2346 | -25.10 | 2374 | 21 | 2363 | 15 |
| 20116 | *Quercus* sp. | 396 | 2345 | -24.36 | 2345 | 21 |  |  |
| 20117 | *Quercus* sp. | 395 | 2344 | -25.44 | 2368 | 21 |  |  |
| 20118 | *Quercus* sp. | 394 | 2343 | -24.71 | 2356 | 21 |  |  |
| 20119 | *Quercus* sp. | 390 | 2339 | -24.78 | 2316 | 20 |  |  |
| 20120 | *Quercus* sp. | 386 | 2335 | -24.95 | 2327 | 22 |  |  |
| 20121 | *Quercus* sp. | 382 | 2331 | -25.27 | 2314 | 21 |  |  |
| 25524 | *Quercus* sp. | 375 | 2324 | -24.36 | 2316 | 20 |  |  |
| 25525 | *Quercus* sp. | 373 | 2322 | -24.24 | 2294 | 20 |  |  |
| 25526 | *Quercus* sp. | 371 | 2320 | -24.33 | 2280 | 20 |  |  |
| 25527 | *Quercus* sp. | 367 | 2316 | -24.17 | 2250 | 20 |  |  |
| 25698 | *Quercus* sp. | 365 | 2314 | -24.25 | 2268 | 20 |  |  |
| 25699 | *Quercus* sp. | 363 | 2312 | -24.25 | 2246 | 19 |  |  |
| 25528 | *Quercus* sp. | 361 | 2310 | -24.72 | 2232 | 20 |  |  |
| 25529 | *Quercus* sp. | 359 | 2308 | -24.83 | 2226 | 20 |  |  |
| 25700 | *Quercus* sp. | 357 | 2306 | -24.41 | 2242 | 20 |  |  |
| 25530 | *Quercus* sp. | 355 | 2304 | -24.58 | 2248 | 20 |  |  |
| 25531 | *Quercus* sp. | 353 | 2302 | -24.57 | 2233 | 19 |  |  |
| 25532 | *Quercus* sp. | 351 | 2300 | -24.56 | 2207 | 20 |  |  |
| 25533 | *Quercus* sp. | 349 | 2298 | -24.26 | 2200 | 20 |  |  |
| 25534 | *Quercus* sp. | 347 | 2296 | -24.05 | 2230 | 20 |  |  |
| 25944 | *Quercus* sp. | 345 | 2294 | -24.54 | 2223 | 20 |  |  |
| 25945 | *Quercus* sp. | 343 | 2292 | -25.77 | 2223 | 19 |  |  |
| 25946 | *Quercus* sp. | 341 | 2290 | -25.25 | 2202 | 20 |  |  |
| 25947 | *Quercus* sp. | 339 | 2288 | -25.74 | 2201 | 20 |  |  |
| 25951 | *Quercus* sp. | 337 | 2286 | -25.07 | 2214 | 20 |  |  |
| 25952 | *Quercus* sp. | 335 | 2284 | -24.88 | 2208 | 20 |  |  |
| 25960 | *Quercus* sp. | 333 | 2282 | -25.49 | 2215 | 20 |  |  |
| 25953 | *Quercus* sp. | 333 | 2282 | -25.35 | 2202 | 20 | 2209 | 15 |
| 25954 | *Quercus* sp. | 331 | 2280 | -25.92 | 2189 | 20 |  |  |
| 25957 | *Quercus* sp. | 329 | 2278 | -25.24 | 2212 | 19 |  |  |
| 25958 | *Quercus* sp. | 327 | 2276 | -24.93 | 2221 | 20 |  |  |
| 25959 | *Quercus* sp. | 325 | 2274 | -25.31 | 2211 | 19 |  |  |
|  |  |  |  |  |  |  |  |  |
| **UCIAMS #** |  | **Tree-Ring  Date BC** | **cal BP** | **N/A** | **^14^C age BP** | **±** |  |  |
| 289050 | *Sequoiadendron giganteum* | 431 | 2380 |  | 2445 | 15 |  |  |
| 289049 | *Sequoiadendron giganteum* | 425 | 2374 |  | 2430 | 15 |  |  |
| 289048 | *Sequoiadendron giganteum* | 421 | 2370 |  | 2405 | 15 |  |  |
| 289047 | *Sequoiadendron giganteum* | 415 | 2364 |  | 2435 | 15 |  |  |
| 289046 | *Sequoiadendron giganteum* | 410 | 2359 |  | 2400 | 15 |  |  |
| 289045 | *Sequoiadendron giganteum* | 405 | 2354 |  | 2445 | 20 |  |  |
| 289044 | *Sequoiadendron giganteum* | 400 | 2349 |  | 2405 | 15 |  |  |
| 289043 | *Sequoiadendron giganteum* | 396 | 2345 |  | 2380 | 20 |  |  |
| 289042 | *Sequoiadendron giganteum* | 390 | 2339 |  | 2340 | 15 |  |  |
| 289041 | *Sequoiadendron giganteum* | 386 | 2335 |  | 2370 | 15 |  |  |
| 289040 | *Sequoiadendron giganteum* | 380 | 2329 |  | 2295 | 15 |  |  |
| 289039 | *Sequoiadendron giganteum* | 375 | 2324 |  | 2290 | 15 |  |  |
| 289038 | *Sequoiadendron giganteum* | 370 | 2319 |  | 2325 | 15 |  |  |
| 289037 | *Sequoiadendron giganteum* | 365 | 2314 |  | 2310 | 15 |  |  |
| 289036 | *Sequoiadendron giganteum* | 362 | 2311 |  | 2280 | 15 |  |  |
| 289035 | *Sequoiadendron giganteum* | 360 | 2309 |  | 2245 | 15 |  |  |
| 289034 | *Sequoiadendron giganteum* | 355 | 2304 |  | 2265 | 15 |  |  |
| 289033 | *Sequoiadendron giganteum* | 351 | 2300 |  | 2240 | 15 |  |  |
| 279400 | *Sequoiadendron giganteum* | 350 | 2299 |  | 2235 | 20 |  |  |
| 279399 | *Sequoiadendron giganteum* | 348 | 2297 |  | 2210 | 15 |  |  |
| 279398 | *Sequoiadendron giganteum* | 346 | 2295 |  | 2220 | 15 |  |  |
| 284204 | *Sequoiadendron giganteum* | 345 | 2294 |  | 2260 | 15 |  |  |
| 279397 | *Sequoiadendron giganteum* | 344 | 2293 |  | 2260 | 15 |  |  |
| 284205 | *Sequoiadendron giganteum* | 343 | 2292 |  | 2225 | 15 |  |  |
| 279396 | *Sequoiadendron giganteum* | 342 | 2291 |  | 2255 | 15 |  |  |
| 284206 | *Sequoiadendron giganteum* | 341 | 2290 |  | 2225 | 15 |  |  |
| 279395 | *Sequoiadendron giganteum* | 340 | 2289 |  | 2235 | 15 |  |  |
| 284207 | *Sequoiadendron giganteum* | 339 | 2288 |  | 2245 | 15 |  |  |
| 279394 | *Sequoiadendron giganteum* | 338 | 2287 |  | 2185 | 20 |  |  |
| 284208 | *Sequoiadendron giganteum* | 337 | 2286 |  | 2230 | 15 |  |  |
| 279393 | *Sequoiadendron giganteum* | 336 | 2285 |  | 2225 | 15 |  |  |
| 284209 | *Sequoiadendron giganteum* | 335 | 2284 |  | 2215 | 15 |  |  |
| 279392 | *Sequoiadendron giganteum* | 334 | 2283 |  | 2190 | 15 |  |  |
| 279391 | *Sequoiadendron giganteum* | 332 | 2281 |  | 2185 | 15 |  |  |
| 279390 | *Sequoiadendron giganteum* | 330 | 2279 |  | 2220 | 15 |  |  |
| 279389 | *Sequoiadendron giganteum* | 328 | 2277 |  | 2190 | 15 |  |  |
| 279388 | *Sequoiadendron giganteum* | 326 | 2275 |  | 2185 | 20 |  |  |
| 279387 | *Sequoiadendron giganteum* | 324 | 2273 |  | 2185 | 15 |  |  |
| 279386 | *Sequoiadendron giganteum* | 322 | 2271 |  | 2210 | 15 |  |  |
| 279385 | *Sequoiadendron giganteum* | 320 | 2269 |  | 2195 | 15 |  |  |
| 279384 | *Sequoiadendron giganteum* | 318 | 2267 |  | 2225 | 20 |  |  |
| 279383 | *Sequoiadendron giganteum* | 316 | 2265 |  | 2220 | 15 |  |  |
| 279382 | *Sequoiadendron giganteum* | 314 | 2263 |  | 2235 | 15 |  |  |
| 279381 | *Sequoiadendron giganteum* | 312 | 2261 |  | 2215 | 15 |  |  |
| 284210 | *Sequoiadendron giganteum* | 311 | 2260 |  | 2235 | 15 |  |  |
| 279380 | *Sequoiadendron giganteum* | 310 | 2259 |  | 2225 | 15 |  |  |
| 284211 | *Sequoiadendron giganteum* | 309 | 2258 |  | 2240 | 15 |  |  |
| 279379 | *Sequoiadendron giganteum* | 308 | 2257 |  | 2260 | 15 |  |  |
| 284212 | *Sequoiadendron giganteum* | 307 | 2256 |  | 2270 | 15 |  |  |
| 278661 | *Sequoiadendron giganteum* | 306 | 2255 |  | 2270 | 15 |  |  |
| 278660 | *Sequoiadendron giganteum* | 304 | 2253 |  | 2260 | 15 |  |  |
| 278659 | *Sequoiadendron giganteum* | 302 | 2251 |  | 2260 | 15 |  |  |
| 278658 | *Sequoiadendron giganteum* | 300 | 2249 |  | 2245 | 15 |  |  |
| 278657 | *Sequoiadendron giganteum* | 298 | 2247 |  | 2250 | 15 |  |  |
| 278656 | *Sequoiadendron giganteum* | 296 | 2245 |  | 2260 | 15 |  |  |
| 278655 | *Sequoiadendron giganteum* | 295 | 2244 |  | 2270 | 15 |  |  |
| 278654 | *Sequoiadendron giganteum* | 294 | 2243 |  | 2250 | 15 |  |  |
| 278653 | *Sequoiadendron giganteum* | 293 | 2242 |  | 2250 | 15 |  |  |
| 278652 | *Sequoiadendron giganteum* | 292 | 2241 |  | 2270 | 15 |  |  |
| 278651 | *Sequoiadendron giganteum* | 291 | 2240 |  | 2245 | 15 |  |  |
| 278650 | *Sequoiadendron giganteum* | 290 | 2239 |  | 2235 | 15 |  |  |
| 278649 | *Sequoiadendron giganteum* | 288 | 2237 |  | 2255 | 15 |  |  |
| 278648 | *Sequoiadendron giganteum* | 286 | 2235 |  | 2270 | 15 |  |  |
| 278647 | *Sequoiadendron giganteum* | 284 | 2233 |  | 2285 | 15 |  |  |
| 278646 | *Sequoiadendron giganteum* | 282 | 2231 |  | 2250 | 15 |  |  |
| 278645 | *Sequoiadendron giganteum* | 280 | 2229 |  | 2260 | 15 |  |  |
| 278644 | *Sequoiadendron giganteum* | 278 | 2227 |  | 2235 | 15 |  |  |
| 278643 | *Sequoiadendron giganteum* | 276 | 2225 |  | 2265 | 15 |  |  |
| 278642 | *Sequoiadendron giganteum* | 274 | 2223 |  | 2270 | 15 |  |  |
| 278641 | *Sequoiadendron giganteum* | 272 | 2221 |  | 2255 | 20 |  |  |
| 278640 | *Sequoiadendron giganteum* | 270 | 2219 |  | 2250 | 15 |  |  |
| 278639 | *Sequoiadendron giganteum* | 268 | 2217 |  | 2240 | 15 |  |  |
| 278638 | *Sequoiadendron giganteum* | 266 | 2215 |  | 2240 | 15 |  |  |
| 278637 | *Sequoiadendron giganteum* | 264 | 2213 |  | 2260 | 15 |  |  |
| 278636 | *Sequoiadendron giganteum* | 262 | 2211 |  | 2240 | 15 |  |  |
| 278635 | *Sequoiadendron giganteum* | 260 | 2209 |  | 2270 | 15 |  |  |
| 278634 | *Sequoiadendron giganteum* | 258 | 2207 |  | 2230 | 15 |  |  |
| 278633 | *Sequoiadendron giganteum* | 256 | 2205 |  | 2235 | 15 |  |  |
| 278632 | *Sequoiadendron giganteum* | 254 | 2203 |  | 2245 | 15 |  |  |
| 278631 | *Sequoiadendron giganteum* | 252 | 2201 |  | 2270 | 15 |  |  |
| 278630 | *Sequoiadendron giganteum* | 250 | 2199 |  | 2270 | 15 |  |  |
