# Supplementary material for: A revised radiocarbon calibration curve 350–250 BCE impacts high-precision dating of the Kyrenia Ship
Source: PLoS One. 2024 Jun 26;19(6):e0302645. doi: 10.1371/journal.pone.0302645 (PMC11207157; doi:10.1371/journal.pone.0302645)
Supplement: S1 File — (DOCX) [file pone.0302645.s002.docx]

**File S1**. **Details on the tree-ring samples dated at UCIAMS and first reported in Table S1 (for details on the tree-ring samples dated at GrM, see [14])**

The UCIAMS calibration samples dated for this project comprise single tree rings from a section of *Sequoiadendron giganteum*. This sample is D22A previously used in earlier radiocarbon calibration work reported by the University of Pennsylvania, labelled as P-SW-ENT-1 ([70] at p.476). This same tree (D22) was also employed in early stable carbon isotope research [71]. It comprises a radial section and came from the Enterprise Mill area of Balch Park, Mountain Home State Forest in California. The section was collected by A.E. Douglass (as his Sequoia no. 22, previously Huntingdon’s no. 195). This section came from part of a trunk, described as a “butt log” from about 20 feet above the forest floor [72] at pp.52-53, plate 7A.

### The tree-ring record across a part of this section (1382 rings) from the total D22A section (labelled as D22SEC) was measured from scans using the CooRecorder software (data listed below measured in 1/100ths of a mm) [73]. This measured section was then crossdated against a set of the regional Sequoia (SEGI) chronologies available from the International Tree Ring Databank (ITRDB): in particular chronologies DSQ411, DSQ421, DSQ431, DSQ451, MHF021, MHF039B, MHF072, MHF075A, MHF075B, MHF091 and MHF301 from the Touchan - Mountain Home combined - SEGI - ITRDB CA717 chronology (from work reported in [74]). Data available from: <https://www.ncei.noaa.gov/access/paleo-search/study/32186>

The crossdating of the D22SEC data was carried out using COFECHA [18] and the results are shown in Figure S1. The Relative Years (RY) 1-1382 chronology from D22SEC is dated as 558 BCE (-558) to 824 CE (824). We note that the SEGI ITRDB CA717 chronology records include a year 0. We have removed this when reporting the calendar ages just stated and as shown in Figure S1 (so that information recorded for year 0 becomes information for 1 BCE and the years BC are thus all -1 year older than those stated in the ITRDB files, e.g., the crossdate for D22SEC RY188 which is returned from the MHF075A chronology as -370 BCE becomes -371 BCE once the year 0 is removed).

D22SEC = D22 Section measured. Relative Years (RY) 1-1382. Tree-ring measurements 1/100ths mm.

D22A 1 Section SEGI

D22A 2 California giant sequoia RY1-1382

MHF 3 Manning

D22SEC 1 100 106 91 76 123 122 114 99 123

D22SEC 10 91 106 106 114 115 129 146 121 108 115

D22SEC 20 114 83 91 129 76 55 121 100 121 123

D22SEC 30 123 135 123 114 99 97 114 138 91 99

D22SEC 40 83 108 91 85 82 85 38 76 106 91

D22SEC 50 76 123 91 99 70 91 114 121 108 114

D22SEC 60 114 97 76 108 97 93 74 70 83 55

D22SEC 70 82 53 70 59 85 99 76 106 106 85

D22SEC 80 114 144 146 97 131 83 46 121 138 138

D22SEC 90 121 108 91 91 106 91 85 85 91 83

D22SEC 100 76 91 85 68 93 76 59 85 76 61

D22SEC 110 83 100 61 121 91 114 114 76 85 82

D22SEC 120 85 76 68 85 100 91 106 91 76 85

D22SEC 130 91 100 83 93 97 91 61 76 91 76

D22SEC 140 108 106 106 114 129 93 76 121 123 98

D22SEC 150 100 85 114 99 85 91 68 91 61 76

D22SEC 160 68 93 59 46 62 68 30 61 99 68

D22SEC 170 76 70 91 61 59 61 99 100 106 91

D22SEC 180 91 61 100 100 76 82 76 100 61 106

D22SEC 190 85 106 76 76 85 68 68 99 91 93

D22SEC 200 76 91 76 76 83 85 61 59 77 53

D22SEC 210 85 61 76 53 76 53 68 93 68 76

D22SEC 220 85 59 61 76 76 91 82 54 64 63

D22SEC 230 77 55 58 64 26 74 78 90 99 87

D22SEC 240 80 80 80 90 90 69 75 67 82 52

D22SEC 250 47 60 63 68 89 75 79 72 73 70

D22SEC 260 74 88 53 62 73 70 86 84 98 115

D22SEC 270 80 87 88 93 85 85 74 64 94 92

D22SEC 280 73 104 112 93 93 92 84 73 85 80

D22SEC 290 101 88 53 81 88 72 84 90 87 78

D22SEC 300 66 62 79 82 73 92 98 100 77 87

D22SEC 310 92 96 79 61 65 88 57 45 45 48

D22SEC 320 56 62 67 63 64 60 53 79 72 73

D22SEC 330 66 70 79 73 76 68 72 74 99 80

D22SEC 340 77 71 48 81 68 93 73 88 107 90

D22SEC 350 79 84 80 92 82 82 60 73 77 71

D22SEC 360 61 69 89 81 87 87 73 74 81 87

D22SEC 370 75 90 94 81 79 80 71 71 72 87

D22SEC 380 73 63 62 72 76 73 60 66 64 75

D22SEC 390 55 101 95 100 74 84 73 81 88 93

D22SEC 400 60 76 64 85 81 62 82 94 67 67

D22SEC 410 86 79 76 71 70 64 67 52 63 70

D22SEC 420 66 67 68 63 73 57 44 47 47 51

D22SEC 430 60 63 56 52 69 69 54 56 47 61

D22SEC 440 61 57 38 47 55 45 61 49 68 64

D22SEC 450 48 49 61 51 46 49 58 64 62 66

D22SEC 460 48 51 44 39 49 44 50 52 58 55

D22SEC 470 46 45 46 39 61 29 57 52 52 36

D22SEC 480 44 70 68 82 71 61 79 69 60 48

D22SEC 490 77 64 73 63 19 43 43 66 55 66

D22SEC 500 54 56 52 46 62 60 43 52 53 53

D22SEC 510 36 50 57 45 66 80 58 66 52 43

D22SEC 520 41 70 52 43 41 53 49 77 56 62

D22SEC 530 58 56 79 70 41 57 60 72 68 41

D22SEC 540 57 75 64 70 68 51 59 72 68 21

D22SEC 550 42 57 59 64 81 64 78 74 76 57

D22SEC 560 72 72 72 57 74 51 76 83 83 72

D22SEC 570 76 72 68 45 64 85 74 68 68 87

D22SEC 580 83 68 87 87 87 66 83 73 73 73

D22SEC 590 68 85 87 82 65 69 59 63 69 80

D22SEC 600 70 66 58 72 54 57 59 48 61 59

D22SEC 610 68 63 65 68 53 59 67 60 48 39

D22SEC 620 60 59 46 47 48 36 58 51 58 54

D22SEC 630 58 52 44 39 41 43 41 34 43 55

D22SEC 640 54 47 47 50 63 55 44 49 61 62

D22SEC 650 77 65 51 51 57 55 40 43 30 50

D22SEC 660 46 57 68 56 70 74 63 35 56 70

D22SEC 670 66 70 70 46 64 73 78 69 60 64

D22SEC 680 70 71 74 74 67 81 64 66 61 63

D22SEC 690 69 60 54 42 73 84 76 70 69 61

D22SEC 700 77 82 78 65 66 59 75 71 54 62

D22SEC 710 56 57 61 47 53 39 35 55 43 62

D22SEC 720 57 60 57 58 53 41 57 48 57 52

D22SEC 730 49 45 55 50 38 53 37 33 45 32

D22SEC 740 46 33 48 35 38 26 32 32 34 43

D22SEC 750 43 39 35 39 33 44 41 35 43 42

D22SEC 760 45 44 53 48 48 42 39 43 40 39

D22SEC 770 39 42 45 38 38 38 29 42 29 38

D22SEC 780 35 30 28 32 29 39 34 36 36 27

D22SEC 790 35 42 42 38 46 45 35 38 36 34

D22SEC 800 41 47 44 62 60 52 60 56 45 49

D22SEC 810 56 50 35 36 42 49 54 56 54 55

D22SEC 820 59 54 53 45 43 44 40 35 39 40

D22SEC 830 25 24 31 49 41 33 44 46 40 37

D22SEC 840 37 35 43 51 48 45 54 41 29 33

D22SEC 850 32 32 43 32 41 49 39 46 48 46

D22SEC 860 42 39 28 30 43 46 46 52 53 39

D22SEC 870 33 42 45 43 46 37 28 27 35 34

D22SEC 880 33 33 34 36 40 40 40 31 32 23

D22SEC 890 29 38 32 31 31 33 27 39 47 42

D22SEC 900 42 39 40 37 44 41 53 62 47 47

D22SEC 910 50 50 42 50 56 60 49 44 43 64

D22SEC 920 58 39 37 50 42 34 31 39 42 44

D22SEC 930 39 42 35 31 33 44 43 39 40 35

D22SEC 940 33 36 27 20 37 38 48 45 39 39

D22SEC 950 44 45 39 37 40 33 37 36 37 37

D22SEC 960 23 26 33 39 47 39 32 28 39 47

D22SEC 970 42 40 38 42 53 43 46 44 45 53

D22SEC 980 47 48 47 61 55 51 58 47 40 42

D22SEC 990 45 38 46 45 39 36 39 39 38 40

D22SEC 1000 40 40 41 38 37 39 32 37 33 38

D22SEC 1010 39 39 50 55 40 38 34 33 35 40

D22SEC 1020 44 40 43 34 35 29 28 37 41 39

D22SEC 1030 38 34 40 27 25 35 45 48 54 54

D22SEC 1040 53 41 46 51 49 52 54 39 48 44

D22SEC 1050 37 46 55 44 36 40 50 42 36 38

D22SEC 1060 51 56 52 42 38 45 43 43 44 41

D22SEC 1070 42 44 40 50 38 35 32 30 40 47

D22SEC 1080 42 42 34 42 39 44 47 46 50 54

D22SEC 1090 37 36 36 37 44 40 38 38 42 35

D22SEC 1100 39 41 51 55 55 34 33 34 33 22

D22SEC 1110 30 33 26 31 51 52 39 36 44 43

D22SEC 1120 42 34 34 35 43 28 24 35 40 39

D22SEC 1130 28 21 24 32 24 27 36 41 34 30

D22SEC 1140 35 49 40 37 46 39 48 47 45 31

D22SEC 1150 31 33 44 23 26 34 28 38 47 45

D22SEC 1160 40 42 44 30 29 43 39 30 27 35

D22SEC 1170 32 35 41 38 35 37 35 34 34 34

D22SEC 1180 38 38 27 28 33 39 38 33 36 42

D22SEC 1190 40 37 41 35 35 46 43 35 36 38

D22SEC 1200 31 45 44 44 37 34 42 46 45 44

D22SEC 1210 40 43 39 25 39 34 39 35 30 39

D22SEC 1220 48 52 40 43 48 40 44 49 51 38

D22SEC 1230 33 27 30 35 46 34 35 26 34 37

D22SEC 1240 37 29 23 33 28 37 42 36 36 35

D22SEC 1250 26 36 34 40 28 29 32 33 40 25

D22SEC 1260 29 32 30 42 40 31 28 33 30 41

D22SEC 1270 41 34 33 40 31 34 40 42 37 33

D22SEC 1280 32 30 42 44 36 37 37 40 48 45

D22SEC 1290 36 36 36 36 28 22 31 33 41 30

D22SEC 1300 30 40 34 36 38 34 36 25 30 30

D22SEC 1310 36 34 23 25 25 11 27 36 34 32

D22SEC 1320 15 27 34 27 25 32 21 36 30 36

D22SEC 1330 15 42 25 47 13 42 40 49 36 32

D22SEC 1340 32 36 30 40 30 38 40 30 17 37

D22SEC 1350 36 30 34 47 40 32 15 32 25 26

D22SEC 1360 17 42 36 23 23 28 27 23 23 13

D22SEC 1370 36 28 49 23 34 32 32 17 19 25

D22SEC 1380 19 19 21 999

**Additional References**

1. Ralph EK, Michael HN. University of Pennsylvania Radiocarbon Dates XII. Radiocarbon 1969; 11:469-481.
2. Craig H. Carbon-13 variations in Sequoia rings and the atmosphere. Science 1954; 119: 141-143.
3. Douglass AE. Climatic cycles and tree-growth: a study of the annual rings of trees in relation to climate and solar activity. Publication no. 289. Washington: Carnegie Institution of Washington; 1919.
4. Maxwell RS, Larsson L-A. 2021. Measuring tree-ring widths using the CooRecorder software application. Dendrochronologia 2021; 67:125841.
5. Touchan R, Black B, Shamir E, Hughes MK, Meko, DM. A multimillennial snow water equivalent reconstruction from giant sequoia tree rings. Climate Dynamics 2021; 56:1507-1518.
