# Supplementary material for: A revised radiocarbon calibration curve 350–250 BCE impacts high-precision dating of the Kyrenia Ship
Source: PLoS One. 2024 Jun 26;19(6):e0302645. doi: 10.1371/journal.pone.0302645 (PMC11207157; doi:10.1371/journal.pone.0302645)
Supplement: S2 File — (DOCX) [file pone.0302645.s003.docx]

**File S2. OxCal runfiles for the models and ‘AMSAdjustedIntCal20’ and ‘AMSAdjustedIntCal20_GrMbased’ calibration data employed 2382-2199 CalBP**

For the Kyrenia samples and data, see Table 1. Those samples not included in the model runs (see explanation in main text) are marked by the lines of code starting with the // indication.

The models using the ‘AMSAdjustedIntCal20’ dataset use a 5-calendar year modelled resolution version of the data (unless otherwise stated) as listed in Table S1 (weighted average [52] where applicable when two measurements by the same laboratory for the same year) inserted into an IntCal20 file for the years 2382 to 2199 CalBP and saved as a curve in OxCal [43] (Figures 2B, 3B, for data file: see below). The data file for the alternative ‘AMSAdjustedIntCal20_GrMbased’ dataset (see main text and Figure 4B) is also listed below.

***Model 1A***

Options()

{

kIterations=3000;

Resolution=5;

Curve="AMSAdjustedIntCal20";

// Exchange the line of code above for the runs reported against IntCal20 with Curve="intcal20.14c";

// Exchange the line of code above for the runs reported against AMSAdjustedIntCal20_GrMbased with Curve="AMSAdjustedIntCal20_GrMbased";

};

Plot()

{

Outlier_Model("General",T(5),U(0,4),"t");

Outlier_Model("SSimple",N(0,2),0,"s");

Sequence ("Kyrenia Ship")

{

Boundary ("Start Kyrenia Ship Wood");

Phase ("Kyrenia Ship Construction Wood/Repairs")

{

D_Sequence("KYR-8 GrM data")

{

Delta_R("Remnant PEG Allowance",50,50);

R_Date("GrM-30706 KYR-8 RY1000-1004 @1002", 2570, 40)

{

Outlier("SSimple",0.05);

};

Gap(5);

R_Date("GrM-30707 KYR-8 RY1005-1009 @1007", 2488, 40)

{

Outlier("SSimple",0.05);

};

Gap(18);

R_Date("GrM-30708 KYR-8 RY1023-1027 @1025", 2520, 20)

{

Outlier("SSimple",0.05);

};

Gap(40);

//R_Date("GrM-30709 KYR-8 RY1043-1047 @1045", 2700, 50)

//{

// Outlier("SSimple",0.05);

//};

//Gap(20);

//Excluded as %C <9%

R_Date("GrM-30711 KYR-8 RY1063-1067 @1065", 2465, 40)

{

Outlier("SSimple",0.05);

};

Gap(20);

R_Date("GrM-30713 KYR-8 RY1083-1087 @1085", 2510, 40)

{

Outlier("SSimple",0.05);

};

Gap(40);

//R_Date("GrM-30714 KYR-8 RY1104-1107 @1105.5", 2330, 80)

//{

// Outlier("SSimple",0.05);

//};

//Gap(19.5);

//Excluded as %C <9% and d13C value anomalous at -26.69 per mille versus all other -21.22 to -22.35 per mille

R_Date("GrM-30716 KYR-8 RY1123-1127 @1125", 2450, 40)

{

Outlier("SSimple",0.05);

};

Gap(9);

Date("Last Extant Tree Ring RY1134",U(-550,-300));

};

Delta_R("No Delta_R",0,0);

V_Sequence ("Ship wood KYR-35 P. nigra")

{

R_Combine ("KYR-35 notional 1001-1005")

{

Outlier("General",0.05);

R_Date ("OxA-31677 KYR-35 1001-1005", 2345,27)

{

Outlier("SSimple",0.05);

};

R_Date ("OxA-31699 KYR-35 1001-1005", 2351,30)

{

Outlier("SSimple",0.05);

};

};

Gap(7,14);

R_Date ("OxA-31700 KYR-35 notional 1006-1014", 2214,21)

{

Outlier("General",0.05);

};

Gap(7,7);

R_Combine ("KYR-35 notional 1015-1019")

{

Outlier("General",0.05);

R_Date("OxA-31840 KYR-35 1015-1019",2200,21)

{

Outlier("SSimple",0.05);

};

R_Date("OxA-31875 KYR-35 1015-1019",2231,21)

{

Outlier("SSimple",0.05);

};

};

Gap(6,6);

R_Combine ("KYR-35 notional RY1021-1025 @RY1023")

{

Outlier("General",0.05);

R_Date("OxA-31841 KYR-35 1021-1025",2215,19)

{

Outlier("SSimple",0.05);

};

R_Date("OxA-31876 KYR-35 1021-1025",2184,33)

{

Outlier("SSimple",0.05);

};

};

Gap(7,7);

Date ("TPQ KYR-35 Last Extant Ring notional RY1030");

};

//R_Date("BM-1639R Wood Pinus halepensis",2780,100)

//{

// Outlier("General",0.05);

//};

R_Date("OxA-31701 Tree Nail C Jodrell 30 KS1 P. brutia/halepensis", 2223,21)

{

Outlier("General",0.05);

};

//R_Date("P-1622 Wood", 2222,43)

//{

// Outlier("General",0.05);

//};

//R_Date ("BM-2294R Pitch", 2390,120)

//{

// Outlier("General",0.05);

//};

};

Boundary ("TPQ");

After("Coin TPQ")

{

C_Date(-334);

};

Sequence()

{

Tau_Boundary("T");

Phase ("Ship contents")

{

R_Date ("OxA-31842 ovis/capra knucklebone W45",2267,19)

{

Outlier("General",0.05);

};

R_Date ("OxA-29333 Prunus dulcis", 2214, 25)

{

Outlier("General",0.05);

};

R_Date ("OxA-29334 Prunus dulcis", 2263, 25)

{

Outlier("General",0.05);

};

R_Date ("OxA-29335 Prunus dulcis", 2274, 26)

{

Outlier("General",0.05);

};

R_Date ("OxA30952 W70-B Prunus dulcis", 2289,31)

{

Outlier("General",0.05);

};

R_Date ("OxA-31033 W70-B Prunus dulcis", 2329,30)

{

Outlier("General",0.05);

};

R_Date ("OxA-31034 W70-B Prunus dulcis", 2291,29)

{

Outlier("General",0.05);

};

//R_Date ("OxA-X-2561-15 Prunus dulcis", 2337, 27)

//{

// Outlier("General",0.05);

//};

//R_Date ("OxA-X-2614-13 Prunus dulcis", 2470, 100)

//{

// Outlier("General",0.05);

//};

R_Date ("OxA-30953 W70-B Prunus dulcis", 2343,29)

{

Outlier("General",0.05);

};

R_Date ("OxA-31032 KS5 USFP Jodrell J.10 Now W69 wood twig", 2307,30)

{

Outlier("General",0.05);

};

//R_Date ("P-1621 Almonds", 2124,60)

//{

// Outlier("General",0.05);

//};

//R_Date ("BM-1588 Almonds", 2210,40)

//{

// Outlier("General",0.05);

//};

//R_Date ("BM-1588A Almonds", 2205,70)

//{

// Outlier("General",0.05);

//};

};

Boundary("LV",U(-325,-270));

};

};

Difference("D","LV","TPQ");

};

***Model 1B***

Options()

{

Resolution=5;

Curve="AMSAdjustedIntCal20";

// Exchange the line of code above for the runs reported against IntCal20 with Curve="intcal20.14c";

// Exchange the line of code above for the runs reported against AMSAdjustedIntCal20_GrMbased with Curve="AMSAdjustedIntCal20_GrMbased";

};

Plot()

{

Outlier_Model("General",T(5),U(0,4),"t");

Outlier_Model("SSimple",N(0,2),0,"s");

Sequence ("Kyrenia Ship")

{

Boundary ("Start Kyrenia Ship Wood");

Phase ("Kyrenia Ship Construction Wood/Repairs")

{

D_Sequence("KYR-8 GrM data")

{

Delta_R("Remnant PEG Allowance",50,50);

R_Date("GrM-30706 KYR-8 RY1000-1004 @1002", 2570, 40)

{

Outlier("SSimple",0.05);

};

Gap(5);

R_Date("GrM-30707 KYR-8 RY1005-1009 @1007", 2488, 40)

{

Outlier("SSimple",0.05);

};

Gap(18);

R_Date("GrM-30708 KYR-8 RY1023-1027 @1025", 2520, 20)

{

Outlier("SSimple",0.05);

};

Gap(40);

//R_Date("GrM-30709 KYR-8 RY1043-1047 @1045", 2700, 50)

//{

// Outlier("SSimple",0.05);

//};

//Gap(20);

//Excluded as %C <9%

R_Date("GrM-30711 KYR-8 RY1063-1067 @1065", 2465, 40)

{

Outlier("SSimple",0.05);

};

Gap(20);

R_Date("GrM-30713 KYR-8 RY1083-1087 @1085", 2510, 40)

{

Outlier("SSimple",0.05);

};

Gap(40);

//R_Date("GrM-30714 KYR-8 RY1104-1107 @1105.5", 2330, 80)

//{

// Outlier("SSimple",0.05);

//};

//Gap(19.5);

//Excluded as %C <9% and d13C value anomalous at -26.69 per mille versus all other -21.22 to -22.35 per mille

R_Date("GrM-30716 KYR-8 RY1123-1127 @1125", 2450, 40)

{

Outlier("SSimple",0.05);

};

Gap(9);

Date("Last Extant Tree Ring RY1134",U(-550,-300));

};

Delta_R("No Delta_R",0,0);

V_Sequence ("Ship wood KYR-35 P. nigra")

{

R_Combine ("KYR-35 notional 1001-1005")

{

Outlier("General",0.05);

R_Date ("OxA-31677 KYR-35 1001-1005", 2345,27)

{

Outlier("SSimple",0.05);

};

R_Date ("OxA-31699 KYR-35 1001-1005", 2351,30)

{

Outlier("SSimple",0.05);

};

};

Gap(7,14);

R_Date ("OxA-31700 KYR-35 notional 1006-1014", 2214,21)

{

Outlier("General",0.05);

};

Gap(7,7);

R_Combine ("KYR-35 notional 1015-1019")

{

Outlier("General",0.05);

R_Date("OxA-31840 KYR-35 1015-1019",2200,21)

{

Outlier("SSimple",0.05);

};

R_Date("OxA-31875 KYR-35 1015-1019",2231,21)

{

Outlier("SSimple",0.05);

};

};

Gap(6,6);

R_Combine ("KYR-35 notional RY1021-1025 @RY1023")

{

Outlier("General",0.05);

R_Date("OxA-31841 KYR-35 1021-1025",2215,19)

{

Outlier("SSimple",0.05);

};

R_Date("OxA-31876 KYR-35 1021-1025",2184,33)

{

Outlier("SSimple",0.05);

};

};

Gap(7,7);

Date ("TPQ KYR-35 Last Extant Ring notional RY1030");

};

//R_Date("BM-1639R Wood Pinus halepensis",2780,100)

//{

// Outlier("General",0.05);

//};

R_Date("OxA-31701 Tree Nail C Jodrell 30 KS1 P. brutia/halepensis", 2223,21)

{

Outlier("General",0.05);

};

//R_Date("P-1622 Wood", 2222,43)

//{

// Outlier("General",0.05);

//};

//R_Date ("BM-2294R Pitch", 2390,120)

//{

// Outlier("General",0.05);

//};

};

Boundary ("TPQ");

After("Coin TPQ")

{

C_Date(-334);

};

Sequence()

{

Boundary("S");

Phase ("Ship contents")

{

R_Date ("OxA-31842 ovis/capra knucklebone W45",2267,19)

{

Outlier("General",0.05);

};

R_Date ("OxA-29333 Prunus dulcis", 2214, 25)

{

Outlier("General",0.05);

};

R_Date ("OxA-29334 Prunus dulcis", 2263, 25)

{

Outlier("General",0.05);

};

R_Date ("OxA-29335 Prunus dulcis", 2274, 26)

{

Outlier("General",0.05);

};

R_Date ("OxA30952 W70-B Prunus dulcis", 2289,31)

{

Outlier("General",0.05);

};

R_Date ("OxA-31033 W70-B Prunus dulcis", 2329,30)

{

Outlier("General",0.05);

};

R_Date ("OxA-31034 W70-B Prunus dulcis", 2291,29)

{

Outlier("General",0.05);

};

//R_Date ("OxA-X-2561-15 Prunus dulcis", 2337, 27)

//{

// Outlier("General",0.05);

//};

//R_Date ("OxA-X-2614-13 Prunus dulcis", 2470, 100)

//{

// Outlier("General",0.05);

//};

R_Date ("OxA-30953 W70-B Prunus dulcis", 2343,29)

{

Outlier("General",0.05);

};

R_Date ("OxA-31032 KS5 USFP Jodrell J.10 Now W69 wood twig", 2307,30)

{

Outlier("General",0.05);

};

//R_Date ("P-1621 Almonds", 2124,60)

//{

// Outlier("General",0.05);

//};

//R_Date ("BM-1588 Almonds", 2210,40)

//{

// Outlier("General",0.05);

//};

//R_Date ("BM-1588A Almonds", 2205,70)

//{

// Outlier("General",0.05);

//};

};

Boundary("LV",U(-325,-270));

};

};

Difference("D","LV","TPQ");

};

***Model 1C***

Options()

{

Resolution=5;

kIterations=3000;

Curve="AMSAdjustedIntCal20";

// Exchange the line of code above for the runs reported against IntCal20 with Curve="intcal20.14c";

// Exchange the line of code above for the runs reported against AMSAdjustedIntCal20_GrMbased with Curve="AMSAdjustedIntCal20_GrMbased";

};

Plot()

{

Outlier_Model("General",T(5),U(0,4),"t");

Outlier_Model("SSimple",N(0,2),0,"s");

Sequence ("Kyrenia Ship")

{

Boundary ("Start Kyrenia Ship Wood");

Phase ("Kyrenia Ship Construction Wood/Repairs")

{

D_Sequence("KYR-8 GrM data")

{

Delta_R("Remnant PEG Allowance",50,50);

R_Date("GrM-30706 KYR-8 RY1000-1004 @1002", 2570, 40)

{

Outlier("SSimple",0.05);

};

Gap(5);

R_Date("GrM-30707 KYR-8 RY1005-1009 @1007", 2488, 40)

{

Outlier("SSimple",0.05);

};

Gap(18);

R_Date("GrM-30708 KYR-8 RY1023-1027 @1025", 2520, 20)

{

Outlier("SSimple",0.05);

};

Gap(40);

//R_Date("GrM-30709 KYR-8 RY1043-1047 @1045", 2700, 50)

//{

// Outlier("SSimple",0.05);

//};

//Gap(20);

//Excluded as %C <9%

R_Date("GrM-30711 KYR-8 RY1063-1067 @1065", 2465, 40)

{

Outlier("SSimple",0.05);

};

Gap(20);

R_Date("GrM-30713 KYR-8 RY1083-1087 @1085", 2510, 40)

{

Outlier("SSimple",0.05);

};

Gap(40);

//R_Date("GrM-30714 KYR-8 RY1104-1107 @1105.5", 2330, 80)

//{

// Outlier("SSimple",0.05);

//};

//Gap(19.5);

//Excluded as %C <9% and d13C value anomalous at -26.69 per mille versus all other -21.22 to -22.35 per mille

R_Date("GrM-30716 KYR-8 RY1123-1127 @1125", 2450, 40)

{

Outlier("SSimple",0.05);

};

Gap(9);

Date("Last Extant Tree Ring RY1134",U(-550,-300));

};

Delta_R("No Delta_R",0,0);

V_Sequence ("Ship wood KYR-35 P. nigra")

{

R_Combine ("KYR-35 notional 1001-1005")

{

Outlier("General",0.05);

R_Date ("OxA-31677 KYR-35 1001-1005", 2345,27)

{

Outlier("SSimple",0.05);

};

R_Date ("OxA-31699 KYR-35 1001-1005", 2351,30)

{

Outlier("SSimple",0.05);

};

};

Gap(7,14);

R_Date ("OxA-31700 KYR-35 notional 1006-1014", 2214,21)

{

Outlier("General",0.05);

};

Gap(7,7);

R_Combine ("KYR-35 notional 1015-1019")

{

Outlier("General",0.05);

R_Date("OxA-31840 KYR-35 1015-1019",2200,21)

{

Outlier("SSimple",0.05);

};

R_Date("OxA-31875 KYR-35 1015-1019",2231,21)

{

Outlier("SSimple",0.05);

};

};

Gap(6,6);

R_Combine ("KYR-35 notional RY1021-1025 @RY1023")

{

Outlier("General",0.05);

R_Date("OxA-31841 KYR-35 1021-1025",2215,19)

{

Outlier("SSimple",0.05);

};

R_Date("OxA-31876 KYR-35 1021-1025",2184,33)

{

Outlier("SSimple",0.05);

};

};

Gap(7,7);

Date ("TPQ KYR-35 Last Extant Ring notional RY1030");

};

//R_Date("BM-1639R Wood Pinus halepensis",2780,100)

//{

// Outlier("General",0.05);

//};

R_Date("OxA-31701 Tree Nail C Jodrell 30 KS1 P. brutia/halepensis", 2223,21)

{

Outlier("General",0.05);

};

//R_Date("P-1622 Wood", 2222,43)

//{

// Outlier("General",0.05);

//};

//R_Date ("BM-2294R Pitch", 2390,120)

//{

// Outlier("General",0.05);

//};

};

Boundary ("TPQ");

After("Coin TPQ")

{

C_Date(-334);

};

Sequence()

{

Tau_Boundary("T");

Phase ("Ship contents")

{

R_Date ("OxA-31842 ovis/capra knucklebone W45",2267,19)

{

Outlier("General",0.05);

};

R_Date ("OxA-29333 Prunus dulcis", 2214, 25)

{

Outlier("General",0.05);

};

R_Date ("OxA-29334 Prunus dulcis", 2263, 25)

{

Outlier("General",0.05);

};

R_Date ("OxA-29335 Prunus dulcis", 2274, 26)

{

Outlier("General",0.05);

};

R_Date ("OxA30952 W70-B Prunus dulcis", 2289,31)

{

Outlier("General",0.05);

};

R_Date ("OxA-31033 W70-B Prunus dulcis", 2329,30)

{

Outlier("General",0.05);

};

R_Date ("OxA-31034 W70-B Prunus dulcis", 2291,29)

{

Outlier("General",0.05);

};

//R_Date ("OxA-X-2561-15 Prunus dulcis", 2337, 27)

//{

// Outlier("General",0.05);

//};

//R_Date ("OxA-X-2614-13 Prunus dulcis", 2470, 100)

//{

// Outlier("General",0.05);

//};

R_Date ("OxA-30953 W70-B Prunus dulcis", 2343,29)

{

Outlier("General",0.05);

};

R_Date ("OxA-31032 KS5 USFP Jodrell J.10 Now W69 wood twig", 2307,30)

{

Outlier("General",0.05);

};

//R_Date ("P-1621 Almonds", 2124,60)

//{

// Outlier("General",0.05);

//};

//R_Date ("BM-1588 Almonds", 2210,40)

//{

// Outlier("General",0.05);

//};

//R_Date ("BM-1588A Almonds", 2205,70)

//{

// Outlier("General",0.05);

//};

};

Boundary("LV",U(-325,-270));

};

};

Tau=(LV-T);

TAU&= U(0,60);

Difference("D","LV","TPQ");

};

***Model 2A***

Options()

{

kIterations=3000;

Resolution=5;

Curve="AMSAdjustedIntCal20";

// Exchange the line of code above for the runs reported against IntCal20 with Curve="intcal20.14c";

// Exchange the line of code above for the runs reported against AMSAdjustedIntCal20_GrMbased with Curve="AMSAdjustedIntCal20_GrMbased";

};

Plot()

{

Outlier_Model("General",T(5),U(0,4),"t");

Outlier_Model("SSimple",N(0,2),0,"s");

Sequence ("Kyrenia Ship")

{

Boundary ("Start Kyrenia Ship Wood");

Phase ("Kyrenia Ship Construction Wood/Repairs")

{

D_Sequence("KYR-8 GrM data")

{

Delta_R("Remnant PEG Allowance",50,50);

R_Date("GrM-30706 KYR-8 RY1000-1004 @1002", 2570, 40)

{

Outlier("SSimple",0.05);

};

Gap(5);

R_Date("GrM-30707 KYR-8 RY1005-1009 @1007", 2488, 40)

{

Outlier("SSimple",0.05);

};

Gap(18);

R_Date("GrM-30708 KYR-8 RY1023-1027 @1025", 2520, 20)

{

Outlier("SSimple",0.05);

};

Gap(40);

//R_Date("GrM-30709 KYR-8 RY1043-1047 @1045", 2700, 50)

//{

// Outlier("SSimple",0.05);

//};

//Gap(20);

//Excluded as %C <9%

R_Date("GrM-30711 KYR-8 RY1063-1067 @1065", 2465, 40)

{

Outlier("SSimple",0.05);

};

Gap(20);

R_Date("GrM-30713 KYR-8 RY1083-1087 @1085", 2510, 40)

{

Outlier("SSimple",0.05);

};

Gap(40);

//R_Date("GrM-30714 KYR-8 RY1104-1107 @1105.5", 2330, 80)

//{

// Outlier("SSimple",0.05);

//};

//Gap(19.5);

//Excluded as %C <9% and d13C value anomalous at -26.69 per mille versus all other -21.22 to -22.35 per mille

R_Date("GrM-30716 KYR-8 RY1123-1127 @1125", 2450, 40)

{

Outlier("SSimple",0.05);

};

Gap(9);

Date("Last Extant Tree Ring RY1134",U(-550,-300));

};

Delta_R("No Delta_R",0,0);

V_Sequence ("Ship wood KYR-35 P. nigra")

{

R_Combine ("KYR-35 notional 1001-1005")

{

Outlier("General",0.05);

R_Date ("OxA-31677 KYR-35 1001-1005", 2345,27)

{

Outlier("SSimple",0.05);

};

R_Date ("OxA-31699 KYR-35 1001-1005", 2351,30)

{

Outlier("SSimple",0.05);

};

};

Gap(7,14);

R_Date ("OxA-31700 KYR-35 notional 1006-1014", 2214,21)

{

Outlier("General",0.05);

};

Gap(7,7);

R_Combine ("KYR-35 notional 1015-1019")

{

Outlier("General",0.05);

R_Date("OxA-31840 KYR-35 1015-1019",2200,21)

{

Outlier("SSimple",0.05);

};

R_Date("OxA-31875 KYR-35 1015-1019",2231,21)

{

Outlier("SSimple",0.05);

};

};

Gap(6,6);

R_Combine ("KYR-35 notional RY1021-1025 @RY1023")

{

Outlier("General",0.05);

R_Date("OxA-31841 KYR-35 1021-1025",2215,19)

{

Outlier("SSimple",0.05);

};

R_Date("OxA-31876 KYR-35 1021-1025",2184,33)

{

Outlier("SSimple",0.05);

};

};

Gap(7,7);

Date ("TPQ KYR-35 Last Extant Ring notional RY1030");

};

//R_Date("BM-1639R Wood Pinus halepensis",2780,100)

//{

// Outlier("General",0.05);

//};

R_Date("OxA-31701 Tree Nail C Jodrell 30 KS1 P. brutia/halepensis", 2223,21)

{

Outlier("General",0.05);

};

//R_Date("P-1622 Wood", 2222,43)

//{

// Outlier("General",0.05);

//};

//R_Date ("BM-2294R Pitch", 2390,120)

//{

// Outlier("General",0.05);

//};

};

After("Coin TPQ")

{

C_Date(-334);

};

Boundary ("LV");

};

Sequence()

{

Tau_Boundary("T");

Phase ("Ship contents")

{

R_Date ("OxA-31842 ovis/capra knucklebone W45",2267,19)

{

Outlier("General",0.05);

};

R_Date ("OxA-29333 Prunus dulcis", 2214, 25)

{

Outlier("General",0.05);

};

R_Date ("OxA-29334 Prunus dulcis", 2263, 25)

{

Outlier("General",0.05);

};

R_Date ("OxA-29335 Prunus dulcis", 2274, 26)

{

Outlier("General",0.05);

};

R_Date ("OxA30952 W70-B Prunus dulcis", 2289,31)

{

Outlier("General",0.05);

};

R_Date ("OxA-31033 W70-B Prunus dulcis", 2329,30)

{

Outlier("General",0.05);

};

R_Date ("OxA-31034 W70-B Prunus dulcis", 2291,29)

{

Outlier("General",0.05);

};

//R_Date ("OxA-X-2561-15 Prunus dulcis", 2337, 27)

//{

// Outlier("General",0.05);

//};

//R_Date ("OxA-X-2614-13 Prunus dulcis", 2470, 100)

//{

// Outlier("General",0.05);

//};

R_Date ("OxA-30953 W70-B Prunus dulcis", 2343,29)

{

Outlier("General",0.05);

};

R_Date ("OxA-31032 KS5 USFP Jodrell J.10 Now W69 wood twig", 2307,30)

{

Outlier("General",0.05);

};

//R_Date ("P-1621 Almonds", 2124,60)

//{

// Outlier("General",0.05);

//};

//R_Date ("BM-1588 Almonds", 2210,40)

//{

// Outlier("General",0.05);

//};

//R_Date ("BM-1588A Almonds", 2205,70)

//{

// Outlier("General",0.05);

//};

};

Boundary("=LV",U(-325,-270));

};

};

***Model 2B***

Options()

{

kIterations=3000;

Resolution=5;

Curve="AMSAdjustedIntCal20";

// Exchange the line of code above for the runs reported against IntCal20 with Curve="intcal20.14c";

// Exchange the line of code above for the runs reported against AMSAdjustedIntCal20_GrMbased with Curve="AMSAdjustedIntCal20_GrMbased";

};

Plot()

{

Outlier_Model("General",T(5),U(0,4),"t");

Outlier_Model("SSimple",N(0,2),0,"s");

Sequence ("Kyrenia Ship")

{

Boundary ("Start Kyrenia Ship Wood");

Phase ("Kyrenia Ship Construction Wood/Repairs")

{

D_Sequence("KYR-8 GrM data")

{

Delta_R("Remnant PEG Allowance",50,50);

R_Date("GrM-30706 KYR-8 RY1000-1004 @1002", 2570, 40)

{

Outlier("SSimple",0.05);

};

Gap(5);

R_Date("GrM-30707 KYR-8 RY1005-1009 @1007", 2488, 40)

{

Outlier("SSimple",0.05);

};

Gap(18);

R_Date("GrM-30708 KYR-8 RY1023-1027 @1025", 2520, 20)

{

Outlier("SSimple",0.05);

};

Gap(40);

//R_Date("GrM-30709 KYR-8 RY1043-1047 @1045", 2700, 50)

//{

// Outlier("SSimple",0.05);

//};

//Gap(20);

//Excluded as %C <9%

R_Date("GrM-30711 KYR-8 RY1063-1067 @1065", 2465, 40)

{

Outlier("SSimple",0.05);

};

Gap(20);

R_Date("GrM-30713 KYR-8 RY1083-1087 @1085", 2510, 40)

{

Outlier("SSimple",0.05);

};

Gap(40);

//R_Date("GrM-30714 KYR-8 RY1104-1107 @1105.5", 2330, 80)

//{

// Outlier("SSimple",0.05);

//};

//Gap(19.5);

//Excluded as %C <9% and d13C value anomalous at -26.69 per mille versus all other -21.22 to -22.35 per mille

R_Date("GrM-30716 KYR-8 RY1123-1127 @1125", 2450, 40)

{

Outlier("SSimple",0.05);

};

Gap(9);

Date("Last Extant Tree Ring RY1134",U(-550,-300));

};

Delta_R("No Delta_R",0,0);

V_Sequence ("Ship wood KYR-35 P. nigra")

{

R_Combine ("KYR-35 notional 1001-1005")

{

Outlier("General",0.05);

R_Date ("OxA-31677 KYR-35 1001-1005", 2345,27)

{

Outlier("SSimple",0.05);

};

R_Date ("OxA-31699 KYR-35 1001-1005", 2351,30)

{

Outlier("SSimple",0.05);

};

};

Gap(7,14);

R_Date ("OxA-31700 KYR-35 notional 1006-1014", 2214,21)

{

Outlier("General",0.05);

};

Gap(7,7);

R_Combine ("KYR-35 notional 1015-1019")

{

Outlier("General",0.05);

R_Date("OxA-31840 KYR-35 1015-1019",2200,21)

{

Outlier("SSimple",0.05);

};

R_Date("OxA-31875 KYR-35 1015-1019",2231,21)

{

Outlier("SSimple",0.05);

};

};

Gap(6,6);

R_Combine ("KYR-35 notional RY1021-1025 @RY1023")

{

Outlier("General",0.05);

R_Date("OxA-31841 KYR-35 1021-1025",2215,19)

{

Outlier("SSimple",0.05);

};

R_Date("OxA-31876 KYR-35 1021-1025",2184,33)

{

Outlier("SSimple",0.05);

};

};

Gap(7,7);

Date ("TPQ KYR-35 Last Extant Ring notional RY1030");

};

//R_Date("BM-1639R Wood Pinus halepensis",2780,100)

//{

// Outlier("General",0.05);

//};

R_Date("OxA-31701 Tree Nail C Jodrell 30 KS1 P. brutia/halepensis", 2223,21)

{

Outlier("General",0.05);

};

//R_Date("P-1622 Wood", 2222,43)

//{

// Outlier("General",0.05);

//};

//R_Date ("BM-2294R Pitch", 2390,120)

//{

// Outlier("General",0.05);

//};

};

After("Coin TPQ")

{

C_Date(-334);

};

Boundary ("LV");

};

Sequence()

{

Boundary("S");

Phase ("Ship contents")

{

R_Date ("OxA-31842 ovis/capra knucklebone W45",2267,19)

{

Outlier("General",0.05);

};

R_Date ("OxA-29333 Prunus dulcis", 2214, 25)

{

Outlier("General",0.05);

};

R_Date ("OxA-29334 Prunus dulcis", 2263, 25)

{

Outlier("General",0.05);

};

R_Date ("OxA-29335 Prunus dulcis", 2274, 26)

{

Outlier("General",0.05);

};

R_Date ("OxA30952 W70-B Prunus dulcis", 2289,31)

{

Outlier("General",0.05);

};

R_Date ("OxA-31033 W70-B Prunus dulcis", 2329,30)

{

Outlier("General",0.05);

};

R_Date ("OxA-31034 W70-B Prunus dulcis", 2291,29)

{

Outlier("General",0.05);

};

//R_Date ("OxA-X-2561-15 Prunus dulcis", 2337, 27)

//{

// Outlier("General",0.05);

//};

//R_Date ("OxA-X-2614-13 Prunus dulcis", 2470, 100)

//{

// Outlier("General",0.05);

//};

R_Date ("OxA-30953 W70-B Prunus dulcis", 2343,29)

{

Outlier("General",0.05);

};

R_Date ("OxA-31032 KS5 USFP Jodrell J.10 Now W69 wood twig", 2307,30)

{

Outlier("General",0.05);

};

//R_Date ("P-1621 Almonds", 2124,60)

//{

// Outlier("General",0.05);

//};

//R_Date ("BM-1588 Almonds", 2210,40)

//{

// Outlier("General",0.05);

//};

//R_Date ("BM-1588A Almonds", 2205,70)

//{

// Outlier("General",0.05);

//};

};

Boundary("=LV",U(-325,-270));

};

};

**Model 2C**

Options()

{

kIterations=3000;

Resolution=5;

Curve="AMSAdjustedIntCal20";

// Exchange the line of code above for the runs reported against IntCal20 with Curve="intcal20.14c";

// Exchange the line of code above for the runs reported against AMSAdjustedIntCal20_GrMbased with Curve="AMSAdjustedIntCal20_GrMbased";

};

Plot()

{

Outlier_Model("General",T(5),U(0,4),"t");

Outlier_Model("SSimple",N(0,2),0,"s");

Sequence ("Kyrenia Ship")

{

Boundary ("Start Kyrenia Ship Wood");

Phase ("Kyrenia Ship Construction Wood/Repairs")

{

D_Sequence("KYR-8 GrM data")

{

Delta_R("Remnant PEG Allowance",50,50);

R_Date("GrM-30706 KYR-8 RY1000-1004 @1002", 2570, 40)

{

Outlier("SSimple",0.05);

};

Gap(5);

R_Date("GrM-30707 KYR-8 RY1005-1009 @1007", 2488, 40)

{

Outlier("SSimple",0.05);

};

Gap(18);

R_Date("GrM-30708 KYR-8 RY1023-1027 @1025", 2520, 20)

{

Outlier("SSimple",0.05);

};

Gap(40);

//R_Date("GrM-30709 KYR-8 RY1043-1047 @1045", 2700, 50)

//{

// Outlier("SSimple",0.05);

//};

//Gap(20);

//Excluded as %C <9%

R_Date("GrM-30711 KYR-8 RY1063-1067 @1065", 2465, 40)

{

Outlier("SSimple",0.05);

};

Gap(20);

R_Date("GrM-30713 KYR-8 RY1083-1087 @1085", 2510, 40)

{

Outlier("SSimple",0.05);

};

Gap(40);

//R_Date("GrM-30714 KYR-8 RY1104-1107 @1105.5", 2330, 80)

//{

// Outlier("SSimple",0.05);

//};

//Gap(19.5);

//Excluded as %C <9% and d13C value anomalous at -26.69 per mille versus all other -21.22 to -22.35 per mille

R_Date("GrM-30716 KYR-8 RY1123-1127 @1125", 2450, 40)

{

Outlier("SSimple",0.05);

};

Gap(9);

Date("Last Extant Tree Ring RY1134",U(-550,-300));

};

Delta_R("No Delta_R",0,0);

V_Sequence ("Ship wood KYR-35 P. nigra")

{

R_Combine ("KYR-35 notional 1001-1005")

{

Outlier("General",0.05);

R_Date ("OxA-31677 KYR-35 1001-1005", 2345,27)

{

Outlier("SSimple",0.05);

};

R_Date ("OxA-31699 KYR-35 1001-1005", 2351,30)

{

Outlier("SSimple",0.05);

};

};

Gap(7,14);

R_Date ("OxA-31700 KYR-35 notional 1006-1014", 2214,21)

{

Outlier("General",0.05);

};

Gap(7,7);

R_Combine ("KYR-35 notional 1015-1019")

{

Outlier("General",0.05);

R_Date("OxA-31840 KYR-35 1015-1019",2200,21)

{

Outlier("SSimple",0.05);

};

R_Date("OxA-31875 KYR-35 1015-1019",2231,21)

{

Outlier("SSimple",0.05);

};

};

Gap(6,6);

R_Combine ("KYR-35 notional RY1021-1025 @RY1023")

{

Outlier("General",0.05);

R_Date("OxA-31841 KYR-35 1021-1025",2215,19)

{

Outlier("SSimple",0.05);

};

R_Date("OxA-31876 KYR-35 1021-1025",2184,33)

{

Outlier("SSimple",0.05);

};

};

Gap(7,7);

Date ("TPQ KYR-35 Last Extant Ring notional RY1030");

};

//R_Date("BM-1639R Wood Pinus halepensis",2780,100)

//{

// Outlier("General",0.05);

//};

R_Date("OxA-31701 Tree Nail C Jodrell 30 KS1 P. brutia/halepensis", 2223,21)

{

Outlier("General",0.05);

};

//R_Date("P-1622 Wood", 2222,43)

//{

// Outlier("General",0.05);

//};

//R_Date ("BM-2294R Pitch", 2390,120)

//{

// Outlier("General",0.05);

//};

};

After("Coin TPQ")

{

C_Date(-334);

};

Boundary ("LV");

};

Sequence()

{

Tau_Boundary("T");

Phase ("Ship contents")

{

R_Date ("OxA-31842 ovis/capra knucklebone W45",2267,19)

{

Outlier("General",0.05);

};

R_Date ("OxA-29333 Prunus dulcis", 2214, 25)

{

Outlier("General",0.05);

};

R_Date ("OxA-29334 Prunus dulcis", 2263, 25)

{

Outlier("General",0.05);

};

R_Date ("OxA-29335 Prunus dulcis", 2274, 26)

{

Outlier("General",0.05);

};

R_Date ("OxA30952 W70-B Prunus dulcis", 2289,31)

{

Outlier("General",0.05);

};

R_Date ("OxA-31033 W70-B Prunus dulcis", 2329,30)

{

Outlier("General",0.05);

};

R_Date ("OxA-31034 W70-B Prunus dulcis", 2291,29)

{

Outlier("General",0.05);

};

//R_Date ("OxA-X-2561-15 Prunus dulcis", 2337, 27)

//{

// Outlier("General",0.05);

//};

//R_Date ("OxA-X-2614-13 Prunus dulcis", 2470, 100)

//{

// Outlier("General",0.05);

//};

R_Date ("OxA-30953 W70-B Prunus dulcis", 2343,29)

{

Outlier("General",0.05);

};

R_Date ("OxA-31032 KS5 USFP Jodrell J.10 Now W69 wood twig", 2307,30)

{

Outlier("General",0.05);

};

//R_Date ("P-1621 Almonds", 2124,60)

//{

// Outlier("General",0.05);

//};

//R_Date ("BM-1588 Almonds", 2210,40)

//{

// Outlier("General",0.05);

//};

//R_Date ("BM-1588A Almonds", 2205,70)

//{

// Outlier("General",0.05);

//};

};

Boundary("=LV",U(-325,-270));

};

Tau=(LV-T);

TAU&= U(0,60);

};

**‘AMSAdjustedIntCal20’ calibration curve**

The following data are inserted into, and replace, the IntCal20 data for CalBP 2382 to 2199:

2382,2422,13

2381,2442,14

2380,2445,15

2375,2437,21

2374,2430,15

2371,2419,21

2370,2405,15

2367,2448,20.3

2364,2435,15

2363,2453,21

2362,2438,15

2361,2414,20.3

2360,2425,21

2359,2400,15

2359,2414,20.3

2358,2413,20.3

2357,2389,15

2356,2436,23.1

2355,2386,21

2354,2445,20

2354,2385,20.3

2353,2365,20.3

2352,2393,20.3

2351,2375,15

2350,2378,21

2349,2405,15

2349,2390,20.3

2348,2361,21

2347,2404,20.3

2346,2363,15

2345,2380,20

2345,2345,21

2344,2368,21

2343,2356,21

2339,2340,15

2339,2316,20.3

2335,2370,15

2335,2327,21.7

2331,2314,21

2329,2295,15

2324,2290,15

2324,2316,19.6

2322,2294,19.6

2320,2280,19.6

2319,2325,15

2316,2250,20.3

2314,2310,15

2314,2268,19.6

2312,2246,18.9

2311,2280,15

2310,2232,19.6

2309,2245,15

2308,2226,19.6

2306,2242,19.6

2304,2265,15

2304,2248,19.6

2302,2233,18.9

2300,2240,15

2300,2207,19.6

2299,2235,20

2298,2200,19.6

2297,2210,15

2296,2230,19.6

2295,2220,15

2294,2260,15

2294,2223,18.9

2293,2260,15

2292,2225,15

2292,2223,18.9

2291,2255,15

2290,2225,15

2290,2202,19.6

2289,2235,15

2288,2245,15

2288,2201,19.6

2287,2185,20

2286,2230,15

2286,2214,19.6

2285,2225,15

2284,2215,15

2284,2208,20.3

2283,2190,15

2282,2209,14

2281,2185,15

2280,2189,19.6

2279,2220,15

2278,2212,18.9

2277,2190,15

2276,2221,19.6

2275,2185,20

2274,2211,18.9

2273,2185,15

2271,2210,15

2269,2195,15

2267,2225,20

2265,2220,15

2263,2235,15

2261,2215,15

2260,2235,15

2259,2225,15

2258,2240,15

2257,2260,15

2256,2270,15

2255,2270,15

2253,2260,15

2251,2260,15

2249,2245,15

2247,2250,15

2245,2260,15

2244,2270,15

2243,2250,15

2242,2250,15

2241,2270,15

2240,2245,15

2239,2235,15

2237,2255,15

2235,2270,15

2233,2285,15

2231,2250,15

2229,2260,15

2227,2235,15

2225,2265,15

2223,2270,15

2221,2255,20

2219,2250,15

2217,2240,15

2215,2240,15

2213,2260,15

2211,2240,15

2209,2270,15

2207,2230,15

2205,2235,15

2203,2245,15

2201,2270,15

2199,2270,15

**‘AMSAdjustedIntCal20_GrMbased’ calibration curve**

The following data are inserted into, and replace, the IntCal20 data for CalBP 2382 to 2199:

2382,2446.0,20.0

2380,2423.6,16.3

2375,2437.0,21.0

2374,2408.6,16.3

2372,2457.0,19.0

2371,2419.0,21.0

2370,2383.6,16.3

2367,2448.0,20.3

2364,2413.6,16.3

2363,2453.0,21.0

2362,2438.0,15.0

2361,2414.0,20.3

2360,2425.0,21.0

2359,2378.6,16.3

2359,2393.0,13.0

2358,2413.0,20.3

2357,2389.0,15.0

2356,2436.0,23.1

2355,2386.0,21.0

2354,2423.6,21.0

2354,2404.0,15.0

2353,2365.0,20.3

2352,2393.0,20.3

2351,2375.0,15.0

2350,2378.0,21.0

2349,2383.6,16.3

2349,2386.0,13.0

2348,2361.0,21.0

2347,2404.0,20.3

2346,2363.0,15.0

2345,2358.6,21.0

2345,2352.0,15.0

2344,2368.0,21.0

2343,2356.0,21.0

2339,2318.0,13.0

2339,2341.0,14.0

2335,2348.6,16.3

2335,2327.0,21.7

2331,2314.0,21.0

2329,2288.0,13.0

2324,2268.6,16.3

2324,2316.0,19.6

2322,2294.0,19.6

2320,2280.0,19.6

2319,2280.0,13.0

2316,2250.0,20.3

2314,2288.6,16.3

2314,2268.0,19.6

2312,2246.0,18.9

2311,2258.6,16.3

2310,2232.0,19.6

2309,2245.0,13.0

2308,2226.0,19.6

2306,2214.0,13.0

2304,2243.6,16.3

2304,2248.0,19.6

2302,2233.0,18.9

2300,2218.6,16.3

2300,2207.0,19.6

2299,2232.0,13.0

2298,2200.0,19.6

2297,2212.0,13.0

2296,2230.0,19.6

2295,2203.0,13.0

2294,2238.6,16.3

2294,2214.0,13.0

2293,2238.6,16.3

2292,2211.0,13.0

2292,2223.0,18.9

2291,2199.0,13.0

2290,2203.6,16.3

2290,2202.0,19.6

2289,2213.6,16.3

2288,2223.6,16.3

2288,2201.0,19.6

2287,2163.6,21.0

2286,2208.6,16.3

2286,2214.0,19.6

2285,2203.6,16.3

2284,2193.6,16.3

2284,2208.0,20.3

2283,2168.6,16.3

2282,2209.0,14.0

2281,2163.6,16.3

2280,2189.0,19.6

2279,2198.6,16.3

2278,2212.0,18.9

2277,2168.6,16.3

2276,2221.0,19.6

2275,2163.6,21.0

2274,2211.0,18.9

2273,2163.6,16.3

2271,2188.6,16.3

2269,2173.6,16.3

2267,2203.6,21.0

2265,2198.6,16.3

2263,2213.6,16.3

2261,2193.6,16.3

2260,2213.6,16.3

2259,2203.6,16.3

2258,2218.6,16.3

2257,2238.6,16.3

2256,2248.6,16.3

2255,2248.6,16.3

2253,2238.6,16.3

2251,2238.6,16.3

2249,2223.6,16.3

2247,2228.6,16.3

2245,2238.6,16.3

2244,2248.6,16.3

2243,2228.6,16.3

2242,2228.6,16.3

2241,2248.6,16.3

2240,2223.6,16.3

2239,2213.6,16.3

2237,2233.6,16.3

2235,2248.6,16.3

2233,2263.6,16.3

2231,2228.6,16.3

2229,2238.6,16.3

2227,2213.6,16.3

2225,2243.6,16.3

2223,2248.6,16.3

2221,2233.6,21.0

2219,2228.6,16.3

2217,2218.6,16.3

2215,2218.6,16.3

2213,2238.6,16.3

2211,2218.6,16.3

2209,2248.6,16.3

2207,2208.6,16.3

2205,2213.6,16.3

2203,2223.6,16.3

2201,2248.6,16.3

2199,2248.6,16.3
