# Supplementary figures and images for: A revised radiocarbon calibration curve 350–250 BCE impacts high-precision dating of the Kyrenia Ship
Source: PLoS One. 2024 Jun 26;19(6):e0302645. doi: 10.1371/journal.pone.0302645 (PMC11207157; doi:10.1371/journal.pone.0302645)

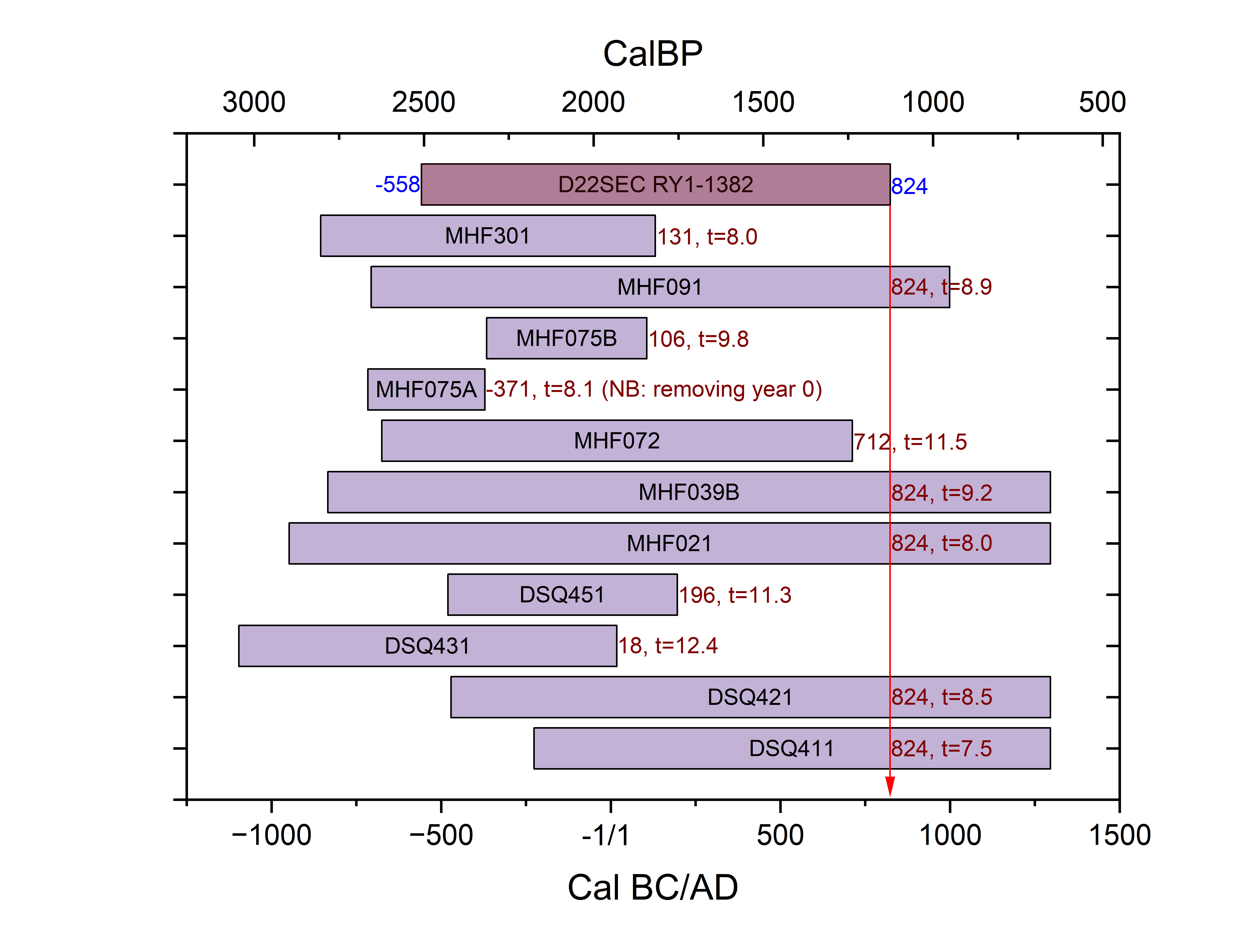

Supplement: S1 Fig — The last year of overlap is indicated and the t value is reported. The D22SEC series is placed (removing a year 0 from the Mountain Home combined chronology) from -558 BCE to 824 CE. (JPG) [file pone.0302645.s005.jpg]

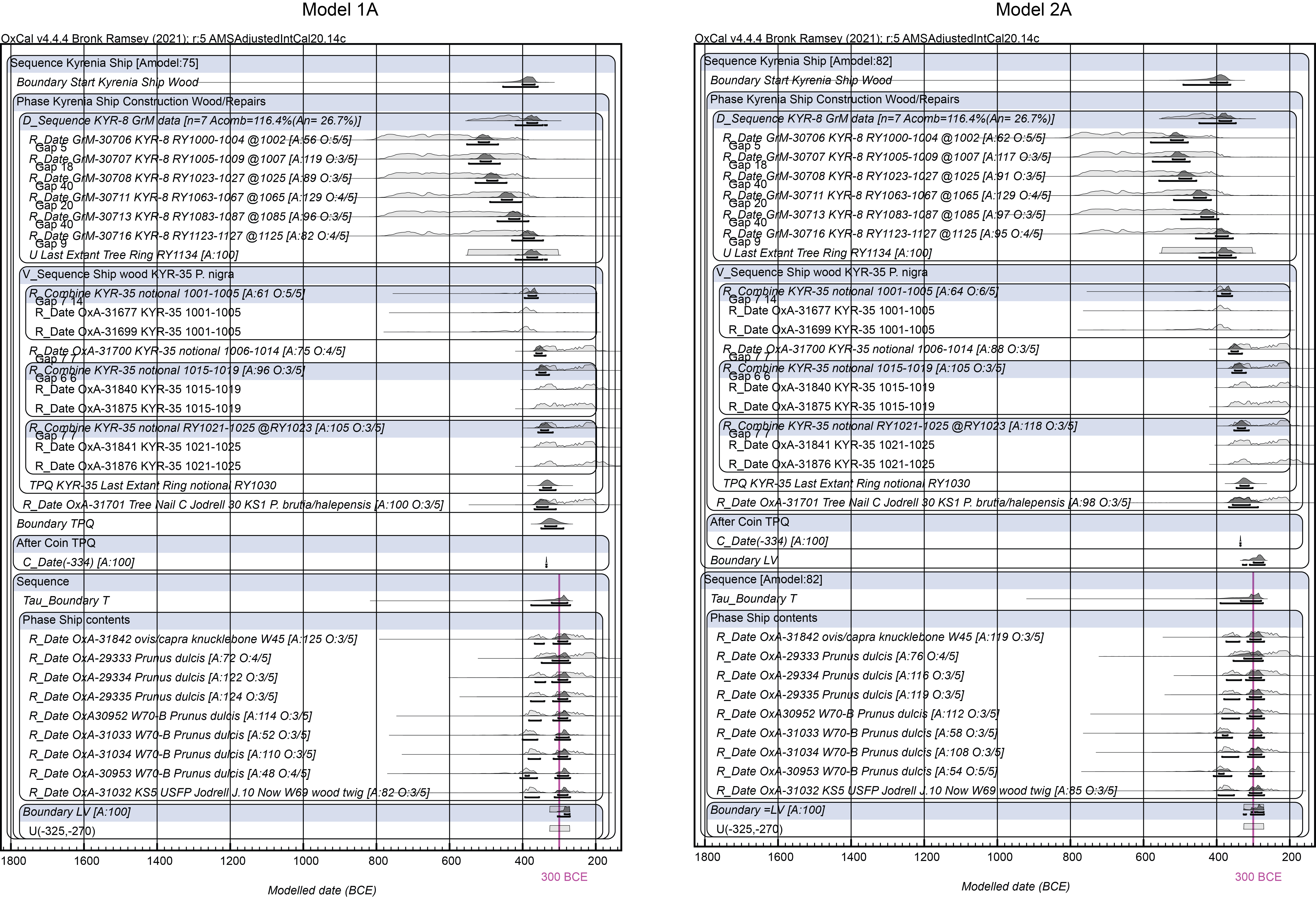

Supplement: S2 Fig — Both these models use an exponential probability Phase for the short-lived materials from the ship (compared with the uniform probability Phase assumption in the B models). The upper and lower lines under the distributions show respectively the 68.3% and 95.4% hpd calendar age ranges. Data from OxCal [43, 45] version 4.4.4. (JPG) [file pone.0302645.s006.jpg]

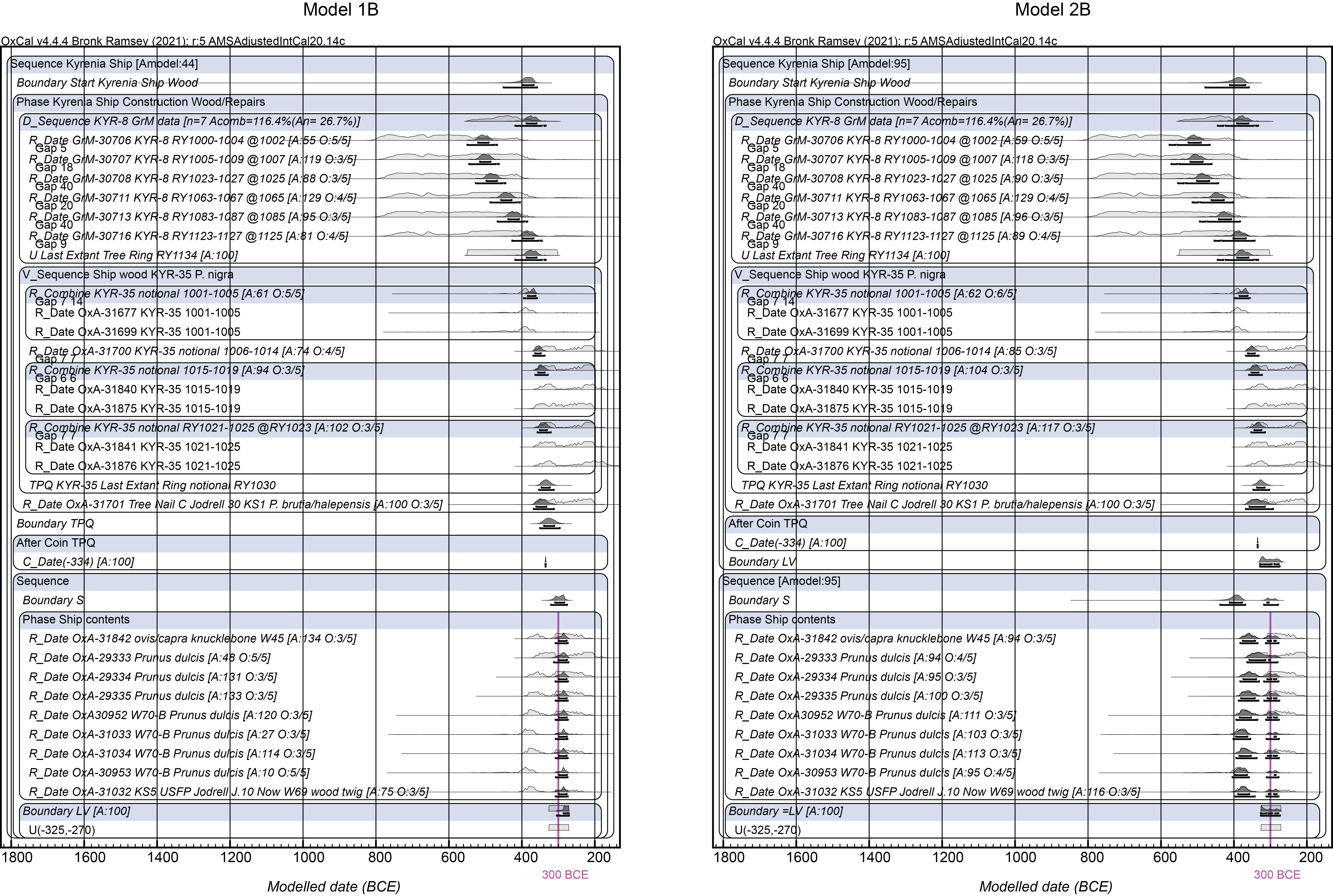

Supplement: S3 Fig — Both these models use a uniform probability Phase for the short-lived materials from the ship (compared with the exponential Phase assumption in the A and C models). The upper and lower lines under the distributions show respectively the 68.3% and 95.4% hpd calendar age ranges. Data from OxCal [43, 45] version 4.4.4. (JPG) [file pone.0302645.s007.jpg]

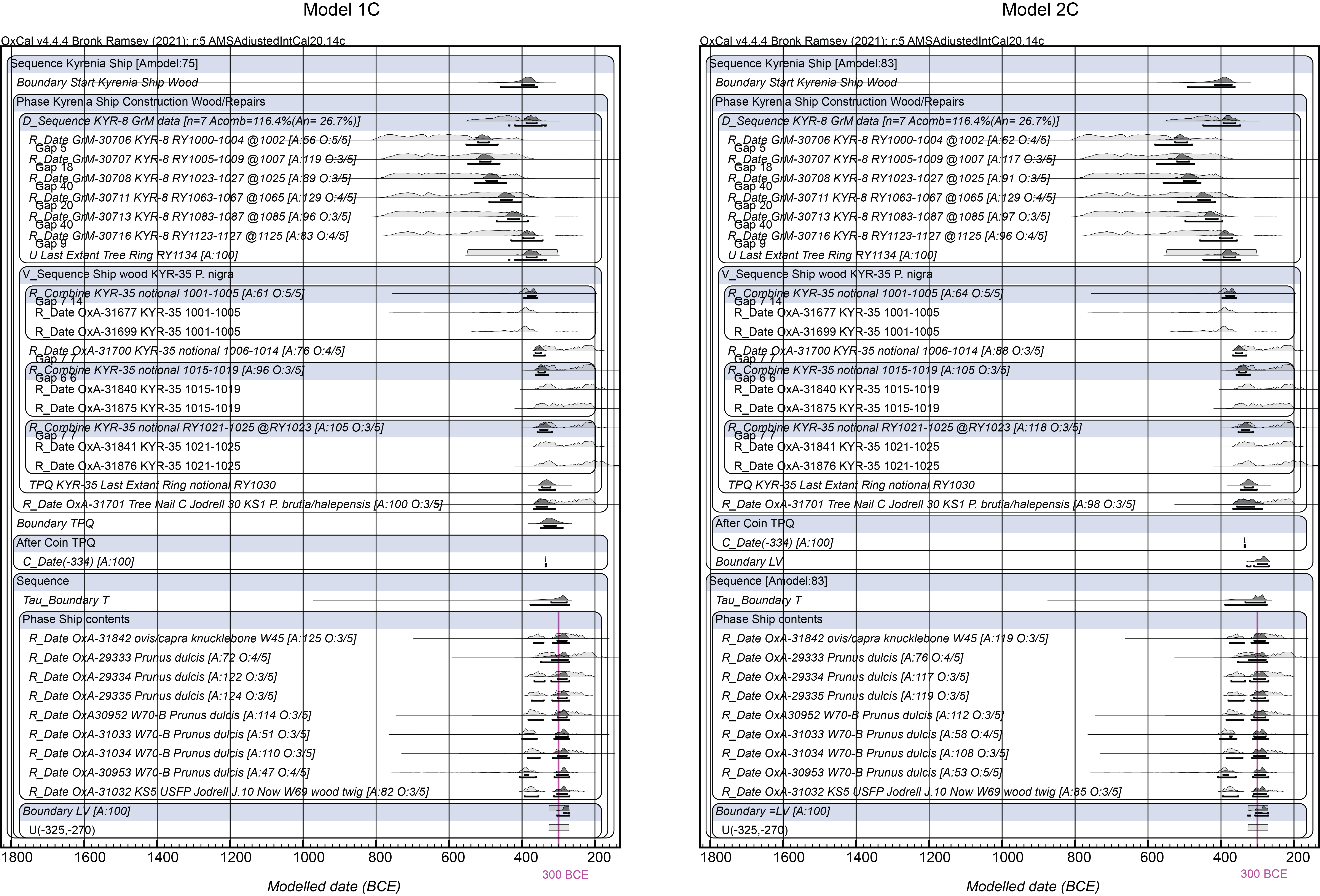

Supplement: S4 Fig — Both these models use an exponential probability Phase for the short-lived materials from the ship like Models 1A and 2A, but now with a time constant, Tau, defining the exponential distribution which has a uniform prior assigned between 0 and 60 calendar years. The upper and lower lines under the distributions show respectively the 68.3% and 95.4% hpd calendar age ranges. Data from OxCal [43, 45] version 4.4.4. (JPG) [file pone.0302645.s008.jpg]

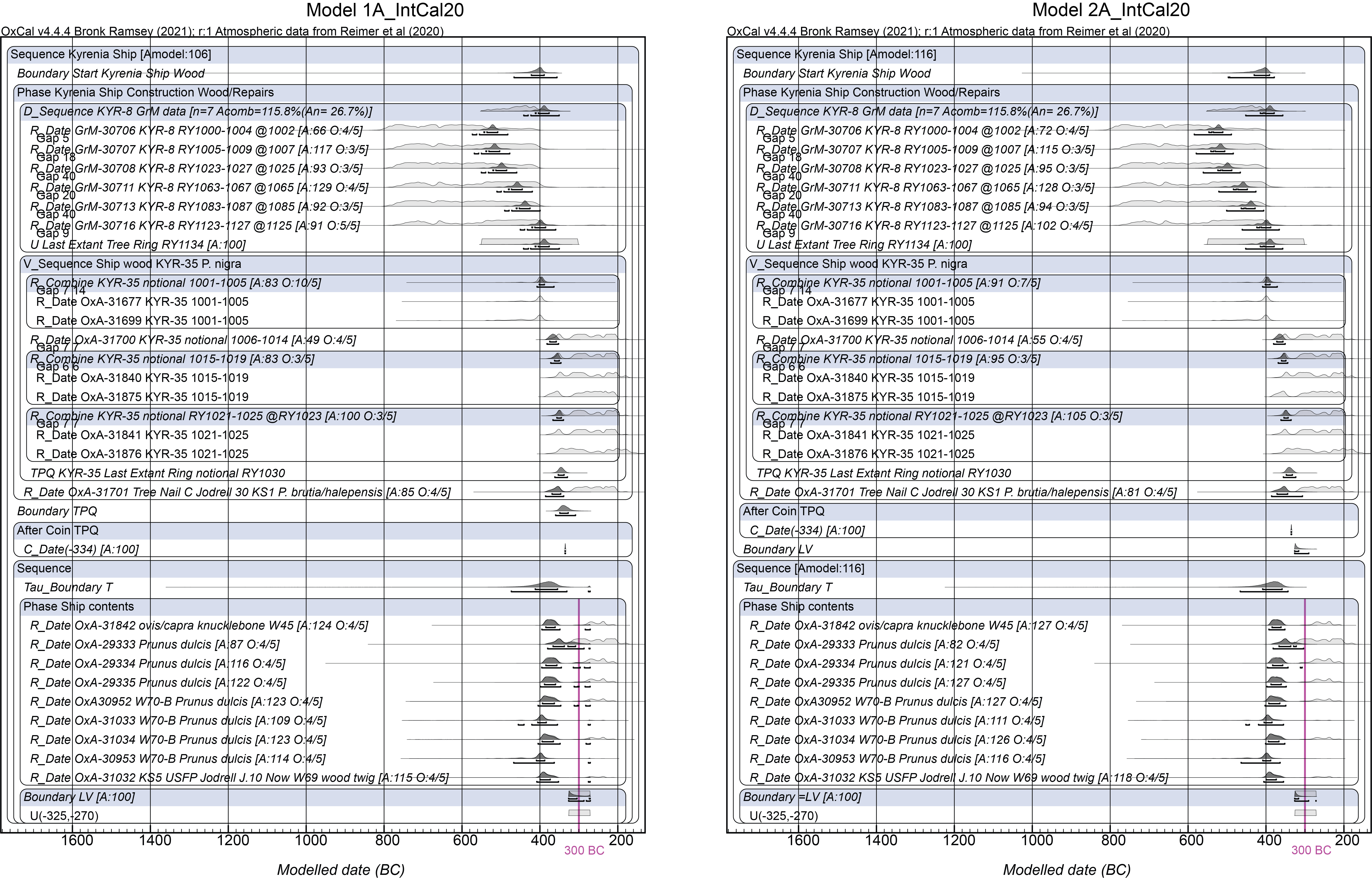

Supplement: S5 Fig — Both these models use an exponential probability Phase for the short-lived materials from the ship (compared with the uniform probability Phase assumption in the B models). The upper and lower lines under the distributions show respectively the 68.3% and 95.4% hpd calendar age ranges. Data from OxCal [43, 45] version 4.4.4. (JPG) [file pone.0302645.s009.jpg]

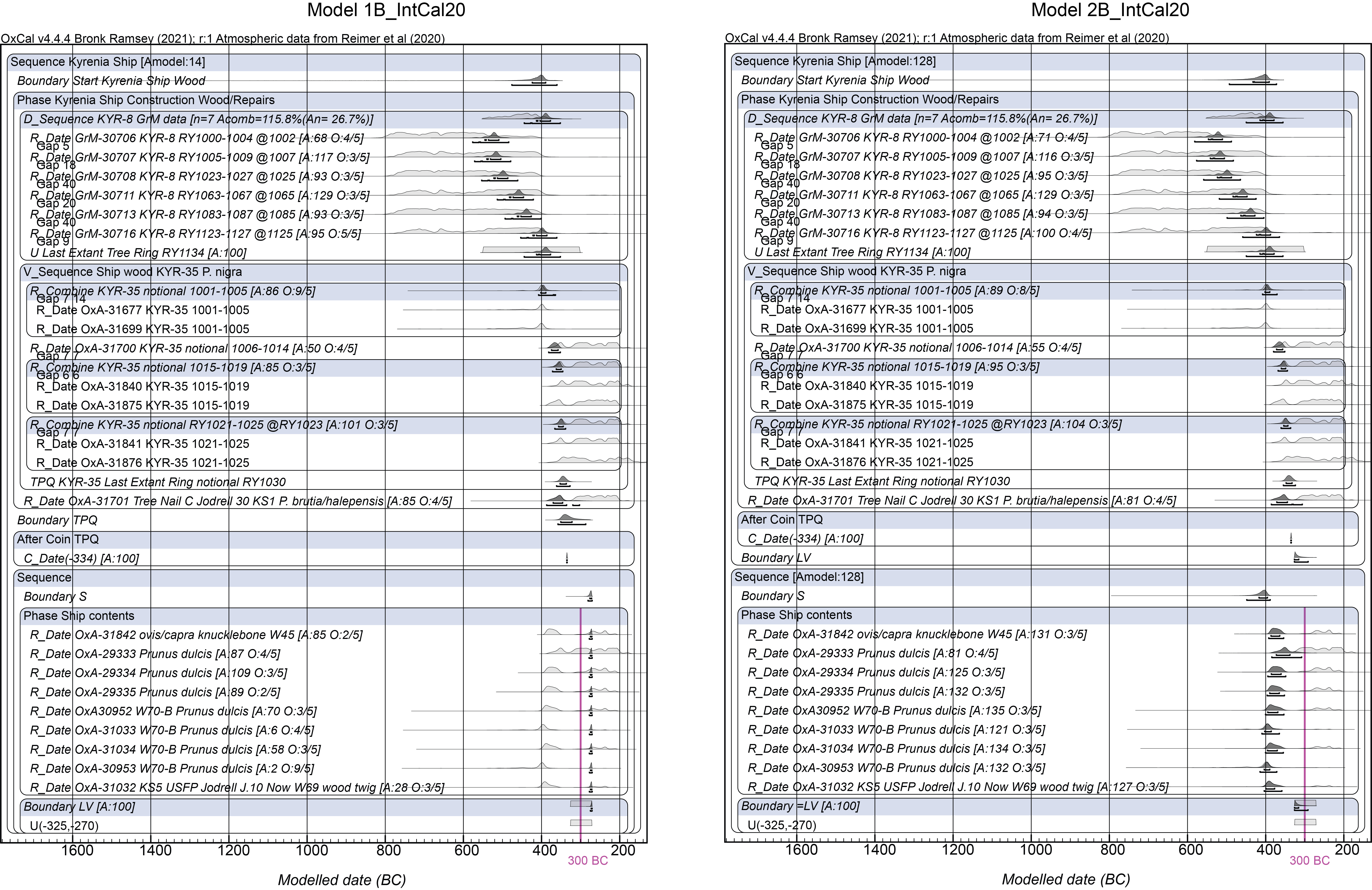

Supplement: S6 Fig — Both these models use a uniform probability Phase for the short-lived materials from the ship (compared with the exponential Phase assumption in the A and C models). The upper and lower lines under the distributions show respectively the 68.3% and 95.4% hpd calendar age ranges. Data from OxCal [43, 45] version 4.4.4. (JPG) [file pone.0302645.s010.jpg]

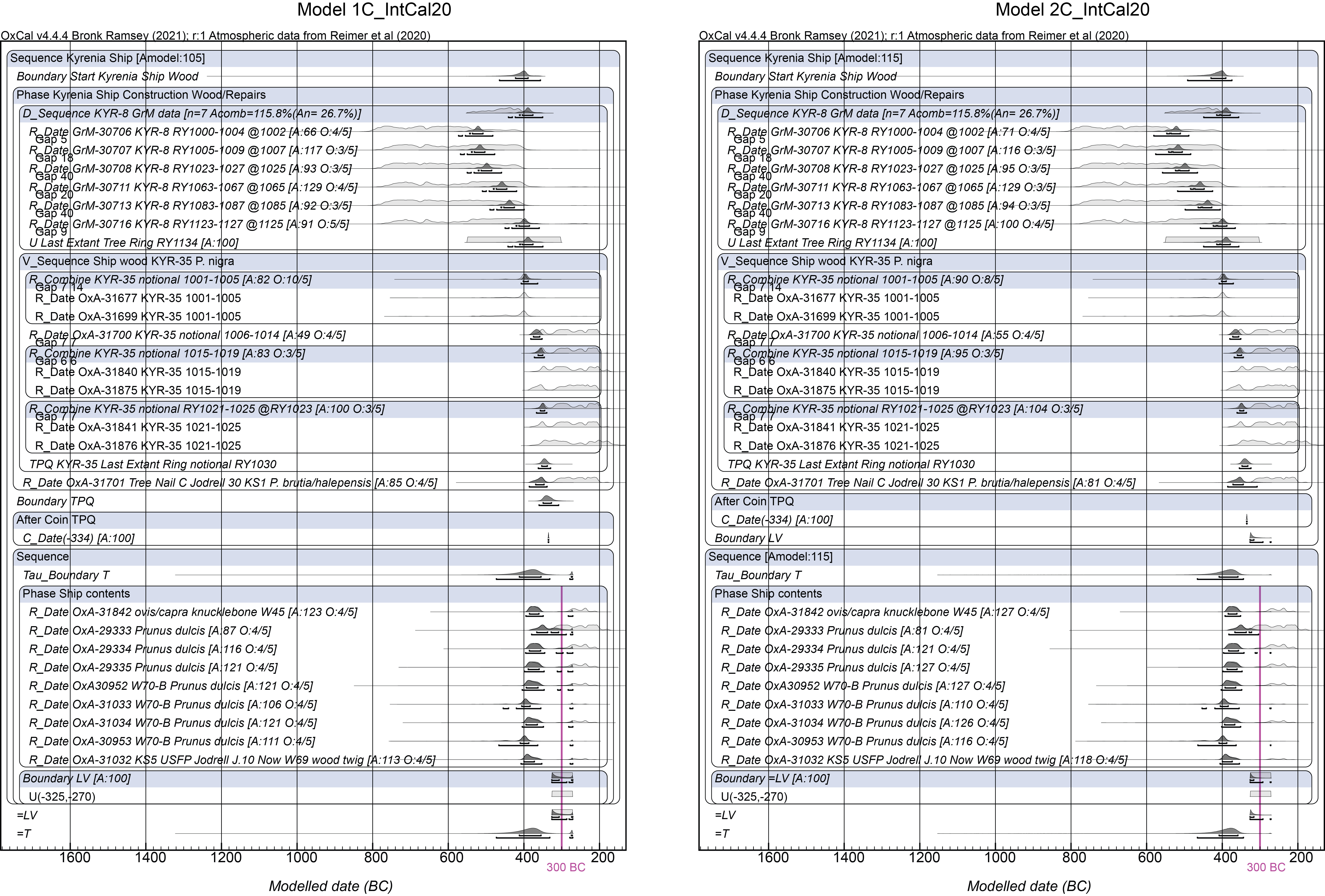

Supplement: S7 Fig — Both these models use an exponential probability Phase for the short-lived materials from the ship like Models 1A and 2A, but now with a time constant, Tau, defining the exponential distribution which has a uniform prior assigned between 0 and 60 calendar years. The upper and lower lines under the distributions show respectively the 68.3% and 95.4% hpd calendar age ranges. Data from OxCal [43, 45] version 4.4.4. (JPG) [file pone.0302645.s011.jpg]

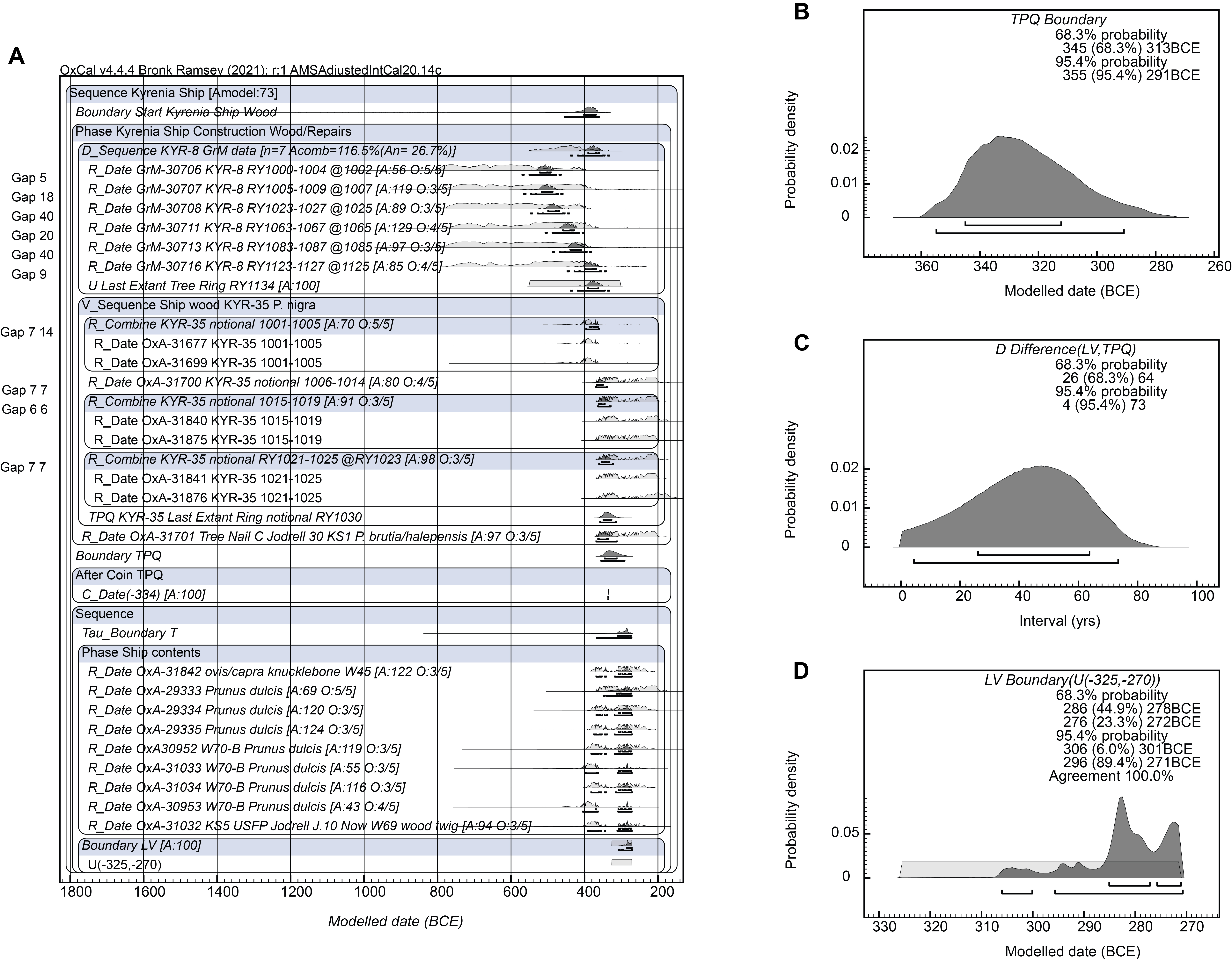

Supplement: S8 Fig — A. whole model. The OxCal keywords, numerical values, and outlining indicate the structure of the model exactly. The light shaded distributions are the non-modelled calibrated calendar probabilities; the smaller dark histograms indicate the modelled probability with the lines under these indicating the 68.3% and 95.4% hpd calendar age ranges. B. Detail of the TPQ Boundary. C. Detail of the LV Boundary. D. Detail of the Difference query (time interval between the TPQ and the LV). Data from OxCal [43, 45] version 4.4.4. (JPG) [file pone.0302645.s012.jpg]

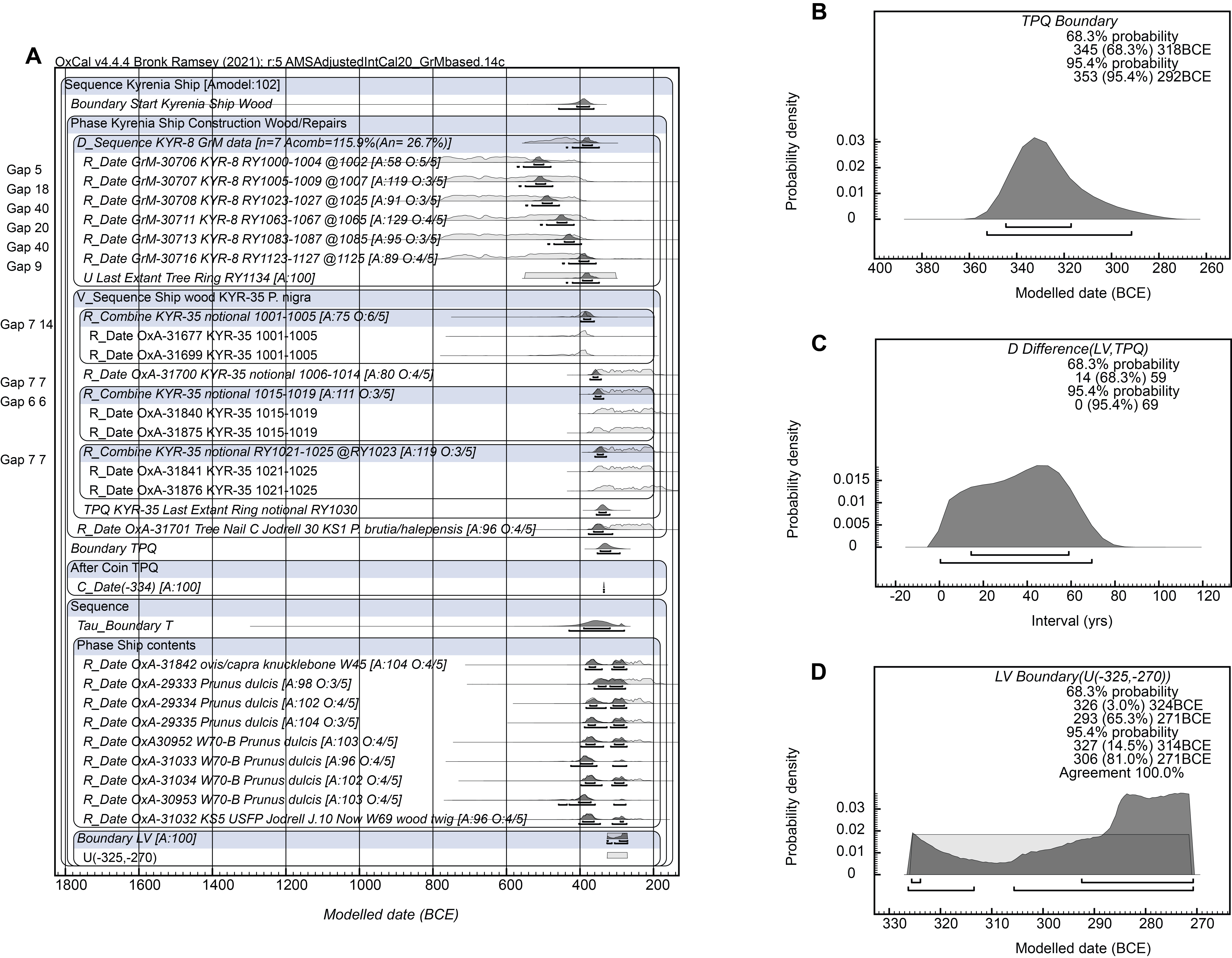

Supplement: S9 Fig — A. whole model. The OxCal keywords, numerical values, and outlining indicate the structure of the model exactly. The light shaded distributions are the non-modelled calibrated calendar probabilities; the smaller dark histograms indicate the modelled probability with the lines under these indicating the 68.3% and 95.4% hpd calendar age ranges. B. Detail of the TPQ Boundary. C. Detail of the LV Boundary. D. Detail of the Difference query (time interval between the TPQ and the LV). Data from OxCal [43, 45] version 4.4.4. (JPG) [file pone.0302645.s013.jpg]
